# Supplementary material for: Gene-specific nonsense-mediated mRNA decay targeting for cystic fibrosis therapy
Source: Nat Commun. 2022 May 27;13:2978. doi: 10.1038/s41467-022-30668-y (PMC9142507; doi:10.1038/s41467-022-30668-y)
Supplement: Supplementary file 1 — Supplementary Information [file 41467_2022_30668_MOESM1_ESM.pdf]

Supplementary Information for Gene-Specific Nonsense-Mediated mRNA Decay Targeting for Cystic Fibrosis Therapy

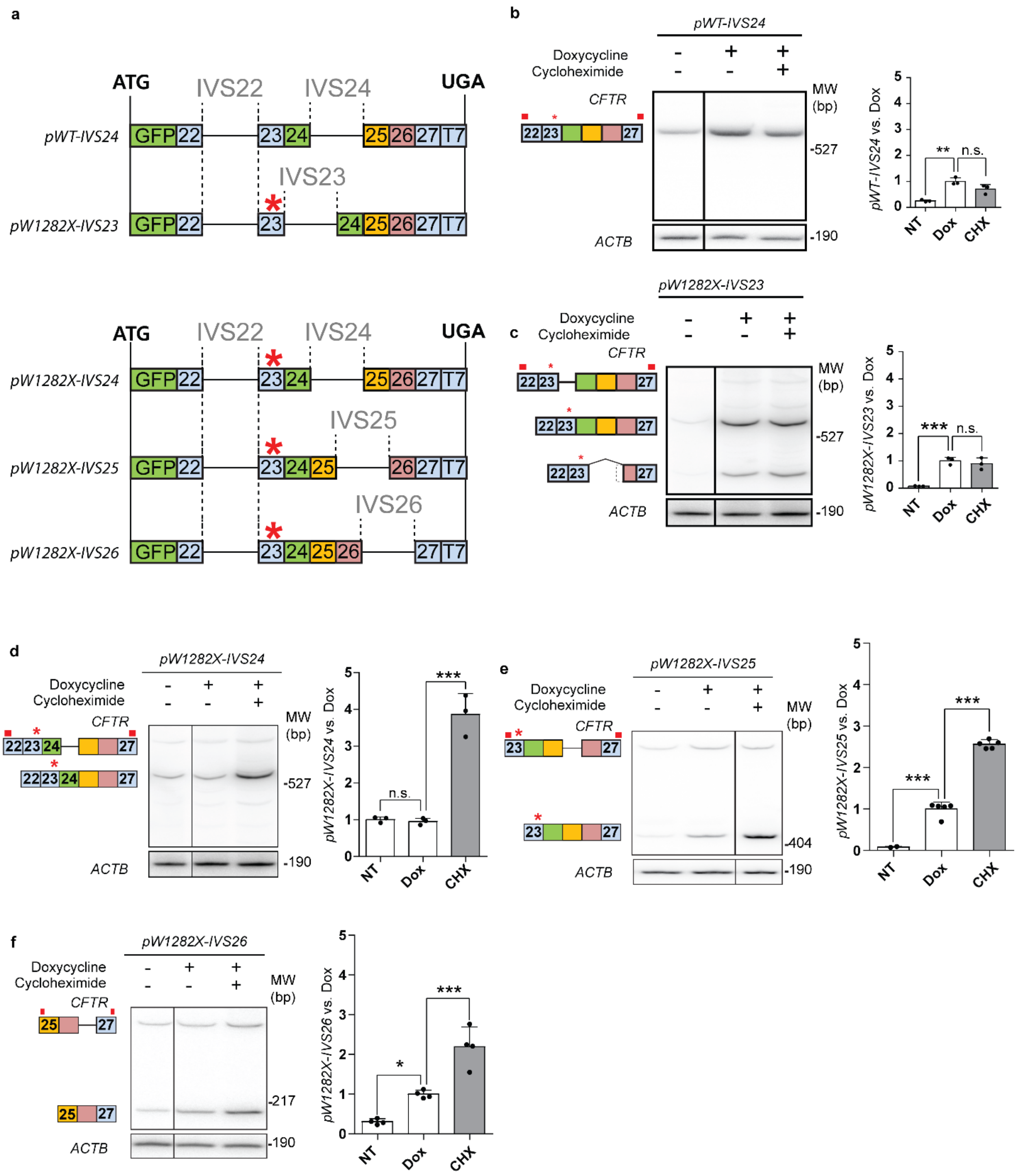

Supplementary Figure 1. Characterization of *CFTR* NMD reporter

a. Schematic of NMD reporters. The numbers in the boxes show the *CFTR* exons present in the NMD reporters. The red asterisk (*) indicates the location of the W1282X mutation. **b-f.** The radioactively labeled RT-PCR images show representative mRNA levels of NMD reporters (b) *pWT-IVS24,* (c) *pW1282X-IVS23,* (d) *pW1282X-IVS24,* (e) *pW1282X-IVS25,* and (f) *pW1282X-IVS26*. *ACTB* mRNA served as an internal reference. The results have been replicated in at least 3 independent biological samples. In panels b-f, all samples were run on the same gel, but lanes were reordered for clarity. Data are represented as mean values ± SD. All data points represent independent biological replicates. The reporter mRNA levels normalized to Dox are shown on the graph on the right side in each panel (n=3 independent measurements in panel b-d, n=2 for NT and n=5 for Dox and CHX in panel e, n=4 in panel f; n.s. $P>0.05$, *$P<0.05$, **$P<0.01$, ***$P<0.001$, one-way ANOVA with Tukey's post-test). NT: No-treatment; Dox: doxycycline 1 μg/mL; CHX: 100 μg/mL cycloheximide for 1 hr. Source data are provided as a Source Data file.

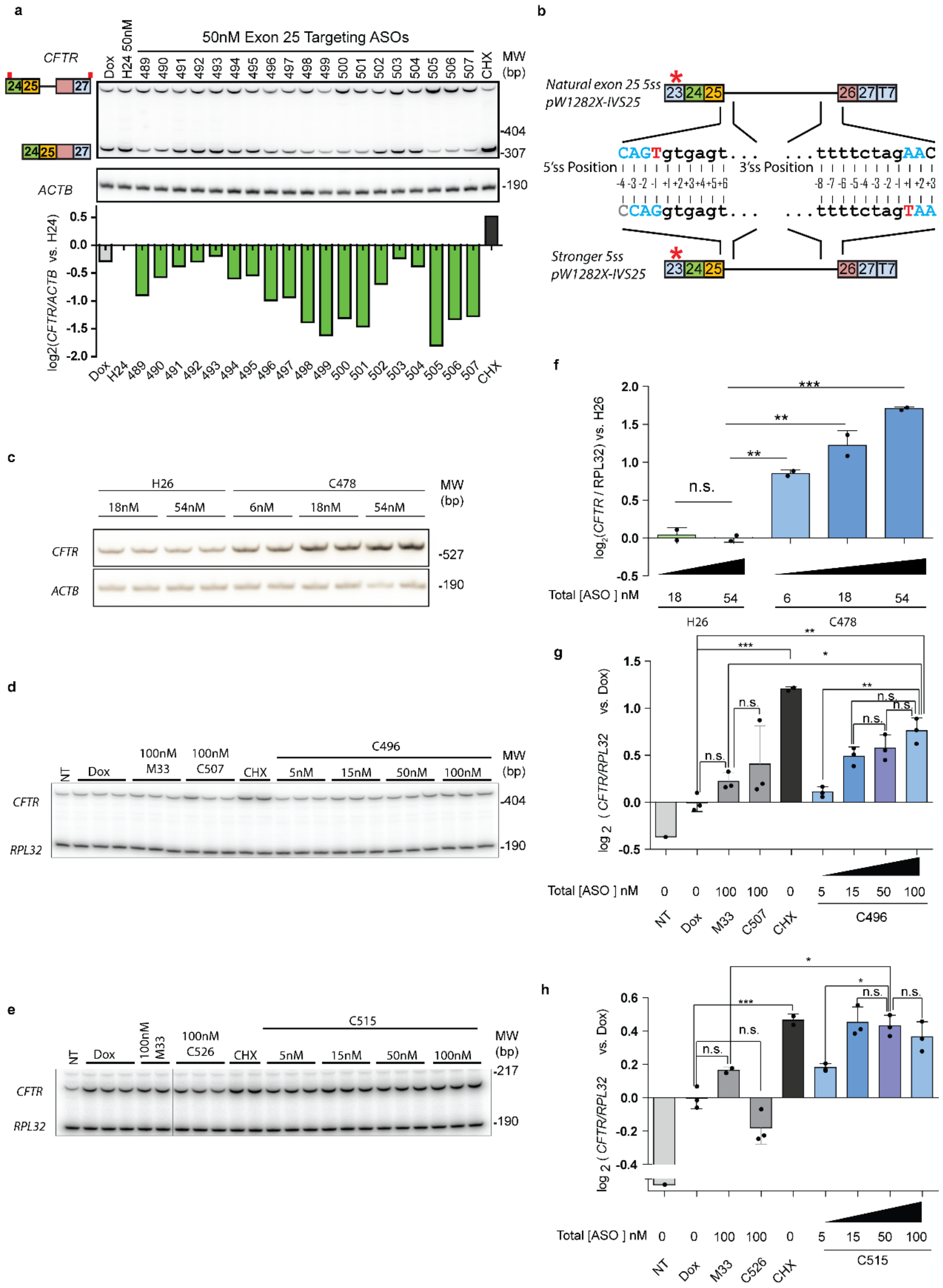
a
50nM Exon 25 Targeting ASOs
CFTR
Dox
H24 50nM
489 490 491 492 493 494 495 496 497 498 499 500 501 502 503 504 505 506 507
CHX
MW (bp)
24 25 27
-404
-307
ACTB
-190
log2(CFTR/ACTB vs. H24)
0.5 0.0 -0.5 -1.0 -1.5 -2.0
b
Natural exon 25 5ss pW1282X-IVS25
23 24 25 26 27 T7
CAGTgtgagt...
...ttttctagAAC
5'ss Position
-4 -3 -2 -1 +1 +2 +3 +4 +5 +6
3'ss Position
-8 -7 -6 -5 -4 -3 -2 -1 +1 +2 +3
CCAGgtgagt...
...ttttctagTAA
Stronger 5ss pW1282X-IVS25
c
H26
C478
18nM 54nM 6nM 18nM 54nM
MW (bp)
CFTR
-527
ACTB
-190
d
NT
Dox
100nM M33
100nM C507
CHX
C496
5nM 15nM 50nM 100nM
MW (bp)
CFTR
-404
RPL32
-190
e
NT
Dox
100nM M33
100nM C526
CHX
C515
5nM 15nM 50nM 100nM
MW (bp)
-217
CFTR
RPL32
-190
f
log2(CFTR / RPL32) vs. H26
2.0 1.5 1.0 0.5 0.0 -0.5
n.s.
**
**
***
Total [ASO] nM
18 54 6 18 54
H26
C478
g
log 2 (CFTR/RPL32 vs. Dox)
1.5 1.0 0.5 0.0 -0.5
***
**
*
n.s
n.s.
**
n.s.
n.s.
n.s.
Total [ASO] nM
0 0 100 100 0 5 15 50 100
NT Dox M33 C507 CHX
C496
h
log 2 (CFTR/RPL32 vs. Dox)
0.6 0.4 0.2 0.0 -0.2 -0.4
*
***
*
n.s.
n.s.
n.s.
n.s.
Total [ASO] nM
0 0 100 100 0 5 15 50 100
NT Dox M33 C526 CHX
C515

Supplementary Figure 2. ASO screen with a weak splice-site reporter, and dose-dependent increase in NMD reporter mRNAs upon candidate-ASO transfection.

a. U2OS cells stably expressing *pW1282X-IVS25* with weak splice sites were transfected with 50 nM negative-control ASOs (H24) or ASOs targeting EJC-binding regions on *CFTR* exon 25, and the log2 fold changes of the reporter levels relative to the control ASO were measured by RT-PCR. *ACTB* served as the internal reference. **b.** The 5'ss of *IVS25* was strengthened by deleting the T at -1, and a T was inserted at +1 of the 3'ss to preserve the same encoded protein sequence *in pW1282X-IVS25.* Upper case = exon sequence; lower case = intron sequence. **c-e.** The lead ASO candidates targeting *CFTR* (c) exon 24, (d) exon 25, or (e) exon 26 or control ASOs (H26 or M33) were tested by transfection into U2OS cells stably expressing the NMD reporters *pW1282X-IVS24, pW1282X-IVS25,* or *pW1282X-IVS26,* respectively. For panel e, all samples were run on the same gel, but lanes were reordered for clarity. **f-h.** The log2 fold changes in NMD reporters in (c-e) were normalized to negative-control ASO transfection. The reporter levels were quantified by RT-PCR; *CFTR* mRNA levels were measured using forward and reverse primers targeting exon 22 and exon 23, respectively. *RPL32* or *ACTB* levels served as internal controls. Data are represented as mean values ± SD. All data points represent independent biological replicates. Panel a (n=1). Panel f (n=2). Panel g and h (n=3). (* $P<0.05$, ** $P<0.01$, and *** $P<0.001$, one-way ANOVA with Tukey's post-test). Abbreviations are as in Supplemental Figure 1. H24, H26, M33: negative-control ASOs. Source data are provided as a Source Data file.

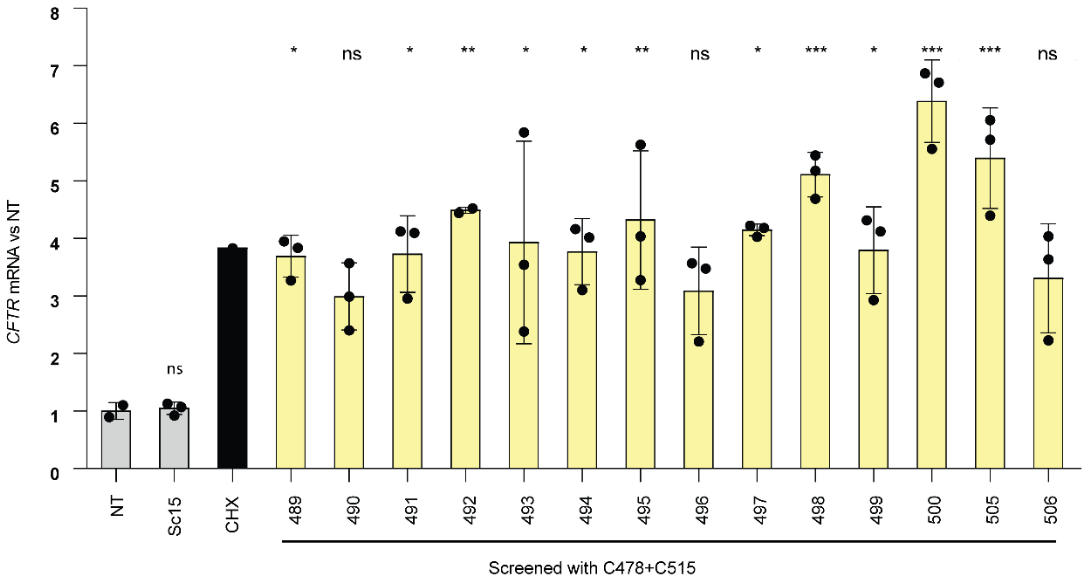

Supplementary Figure 3. Effect of the ASO cocktail on DLD1-W1282X cells.

Various exon-25-targeting ASOs were combined with C478 and C515, and transfected into DLD1-W1282X cells. The identity of the ASOs and nominal concentrations used for transfection are indicated. (n=3 independent treatments and transfections, n.s. P>0.05, *P<0.05, **P<0.01, ***P<0.001 versus NT, one-way ANOVA with Tukey's post-test). All mRNA levels were measured by RT-qPCR; *CFTR* mRNA levels were measured using forward and reverse primers targeting exon 22 and exon 23, respectively. *RPL32* mRNA served as an internal reference. Data are represented as mean values ± SD. All data points represent independent biological replicates. Source data are provided as a Source Data file.

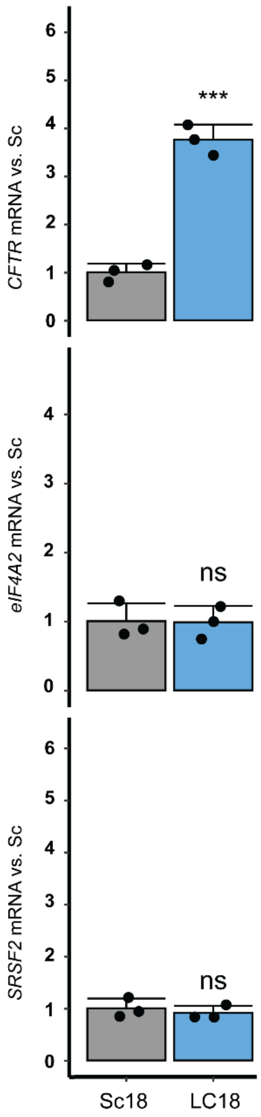

Supplementary Figure 4. Gene-specific NMD inhibition by the 18mer ASO cocktail.

Endogenous NMD-sensitive mRNA levels in 16HBE-W1282X cells treated with 120 nM scramble ASO (Sc18) or 120 nM lead ASO cocktail C24/25/26 (LC18) (n=3 independent treatments, n.s. P>0.05, ***P<0.001 versus NT, two-tailed Student's t-test). mRNA levels were quantified by RT-qPCR; *CFTR* mRNA levels were measured using forward and reverse primers targeting exon 22 and exon 23, respectively. *RPL32* served as an internal control. Data are represented as mean values ± SD. All data points represent independent biological replicates.

Sc18=18-mer Scramble ASO; LC18=Lead ASO cocktail C24/25/26. Source data are provided as a Source Data file.

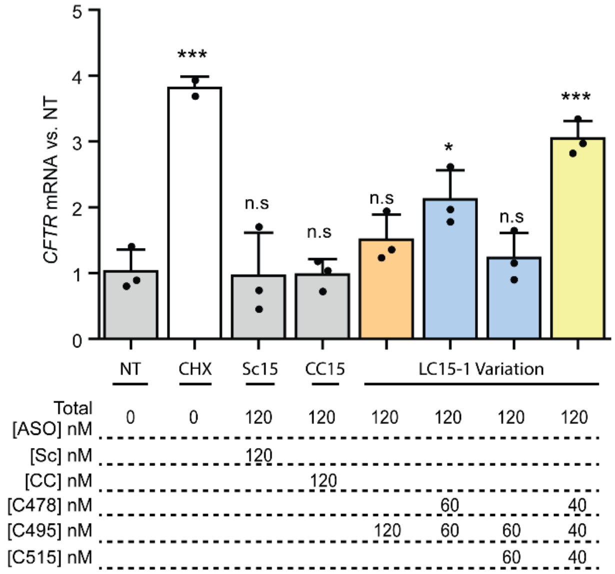

Supplementary Figure 5. Effect of targeting different numbers of EJCs on NMD of *CFTR-W1282X* mRNA in DLD1-W1282X cells.

The number of required EJCs targeted by the lead ASOs (C478, C495, and C515) was assessed by transfecting DLD1-W1282X cells with one, two, or three EJC-targeting ASOs at the same total nominal concentration. *CFTR* mRNA levels were measured by RT-qPCR using forward and reverse primers targeting exon 22 and exon 23, respectively. Data are represented as mean values ± SD. All data points represent independent biological replicates. (n=3 independent treatments and transfections; n.s. P>0.05, *P<0.05, **P<0.01, ***P<0.001 versus NT, one-way ANOVA with Dunnett's post-test). Source data are provided as a Source Data file.

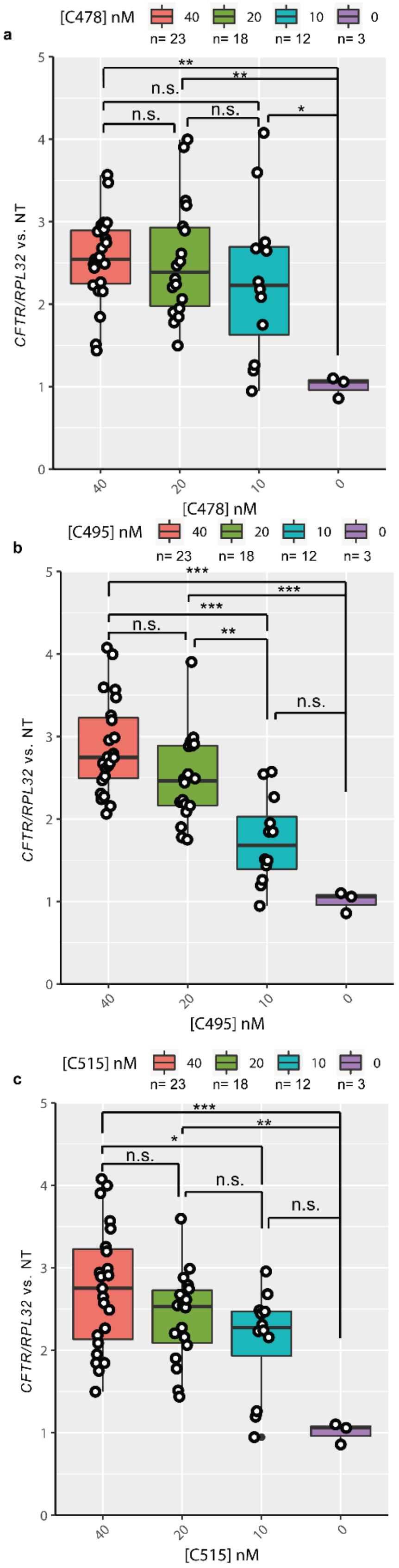
a
[C478] nM 40 20 10 0
n= 23 n= 18 n= 12 n= 3
CFTR/RPL32 vs. NT
**
**
n.s.
n.s.
n.s.
*
[C478] nM
b
[C495] nM 40 20 10 0
n= 23 n= 18 n= 12 n= 3
CFTR/RPL32 vs. NT
***
***
***
n.s.
**
n.s.
[C495] nM
c
[C515] nM 40 20 10 0
n= 23 n= 18 n= 12 n= 3
CFTR/RPL32 vs. NT
***
**
*
n.s.
n.s.
n.s.
[C515] nM

Supplementary Figure 6. Effect of limiting ASO concentration on NMD inhibition.

a-c. The effect of limiting the dose of each ASO on *CFTR* mRNA levels in 16HBE-W1282X cells was quantified by categorizing the three-ASO combinations tested in Figure 2G, according to the variable concentration of (a) C478, (b) C495, and (c) C515 ASO. The distribution of *CFTR* mRNA levels for each ASO concentration is shown as boxplots (Box = first to third quartile; horizontal line through the box = median; Vertical lines below and above the boxes = minimum to the first quartile and third quartile to maximum, respectively; individual data points plotted as open circles). Each dot represents an independent transfection of an ASO cocktail. The n number for each ASO concentration group is indicated in the figure. All mRNA levels were measured by RT-qPCR; *CFTR* mRNA levels were measured using forward and reverse primers targeting exon 22 and exon 23, respectively. *RPL32* mRNA served as internal reference. (n.s. $P>0.05$, $^{*}P<0.05$, $^{**}P<0.01$, $^{***}P<0.001$, one-way ANOVA with Tukey's post-test). Source data are provided as a Source Data file.

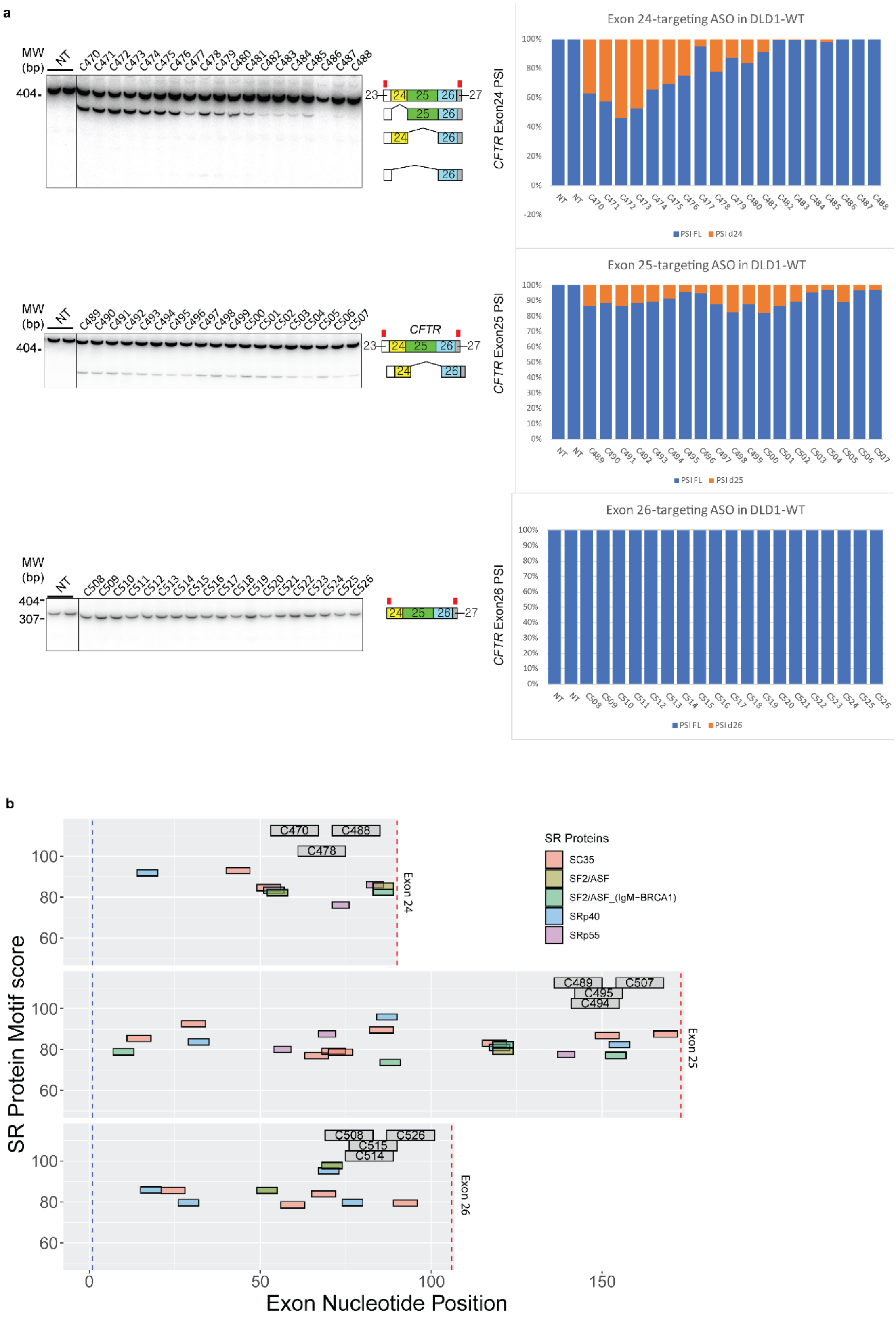

Supplementary Figure 7. Effects of single 15mer ASOs on splicing of *CFTR-WT* mRNA.

a. All 57 candidate ASOs (19 ASOs per exon) were individually transfected at a nominal concentration of 50 nM into DLD1-WT cells. *CFTR* mRNA exon 24, 25, and 26 percent-spliced-in (PSI) values were calculated from the RT-PCR data. Primers targeting the exons 23 and 27 were used to measure PSI of exons 24 and 25; primers targeting the exons 24 and 27 were used to measure PSI of exon 26. All samples in each image were run on the same gel, but lanes were reordered for clarity. n=1 for all treatments. **b.** SR protein motif analysis by ESEfinder. (Original SR protein names; new names: SC35 = SRSF2, SF2/ASF = SRSF1, SRp40 = SRSF5, SRp55 = SRSF6). The small grey boxes show the target sites of the indicated lead ASOs. NT=No treatment. Source data are provided as a Source Data file.

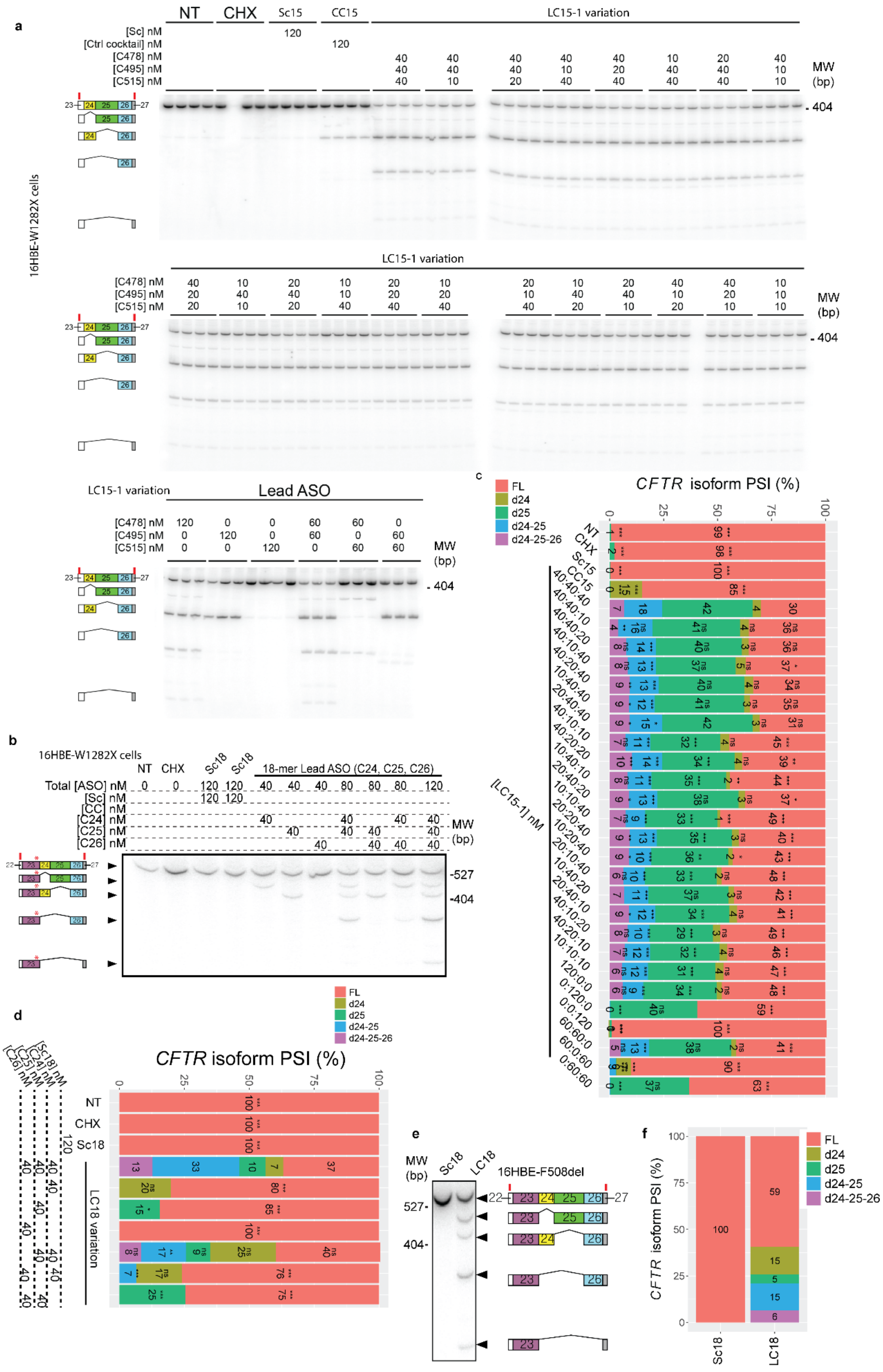

a
NT
CHX
Sc15
CC15
LC15-1 variation
[Sc] nM
[Ctrl cocktail] nM
[C478] nM
[C495] nM
[C515] nM
MW (bp)
404
16HBE-W1282X cells
Lead ASO
b
16HBE-W1282X cells
18-mer Lead ASO (C24, C25, C26)
Total [ASO] nM
[Sc] nM
[CC] nM
[C24] nM
[C25] nM
[C26] nM
527
404
c
CFTR isoform PSI (%)
FL
d24
d25
d24-25
d24-25-26
[LC15-1] nM
d
CFTR isoform PSI (%)
LC18 variation
[Sc18] nM
[C24] nM
[C25] nM
[C26] nM
e
16HBE-F508del
Sc18
LC18
f
CFTR isoform PSI (%)

Supplementary Figure 8. Effects of 15-mer or 18-mer ASO cocktails on *CFTR* splicing in 16HBE-W1282X cells; related to Figure 2h.

a. Complete RT-PCR results of *CFTR* mRNA splicing patterns in 16HBE-W1282X cells transfected with various combinations of C478, C495, and C515 ASOs. **b.** Representative RT-PCR results of *CFTR* mRNA splicing patterns in 16HBE-W1282X cells transfected with various combinations of 18mer ASOs C24, C25, and C26. Primers targeting the exons 23 and 27 were used to measure PSI. **c-d.** Mean PSI of each *CFTR* isoform in 16HBE-W1282X cells in (c) Panel a and (d) Panel b. **e.** Representative RT-PCR results of *CFTR* mRNA splicing patterns in 16HBE-F508del cells transfected with the lead 18mer ASO cocktail. **f.** Mean PSI of each *CFTR* isoform in 16HBE-F508del cells in Panel e. Data are represented as mean values of independent biological replicates. The results have been replicated in at least 3 independent biological samples for panels b and e. For Panel c-d, n=4 and 3, respectively. In Panels c-d, n.s. P>0.05, *P<0.05, **P<0.01, ***P<0.001, one-way ANOVA with Dunnett's post-test, versus each isoform in (Panel c) LC15-1=40:40:40 nM or (Panel d) LC18=40:40:40 nM. Abbreviations as in Figure 1 and 2. Source data are provided as a Source Data file.

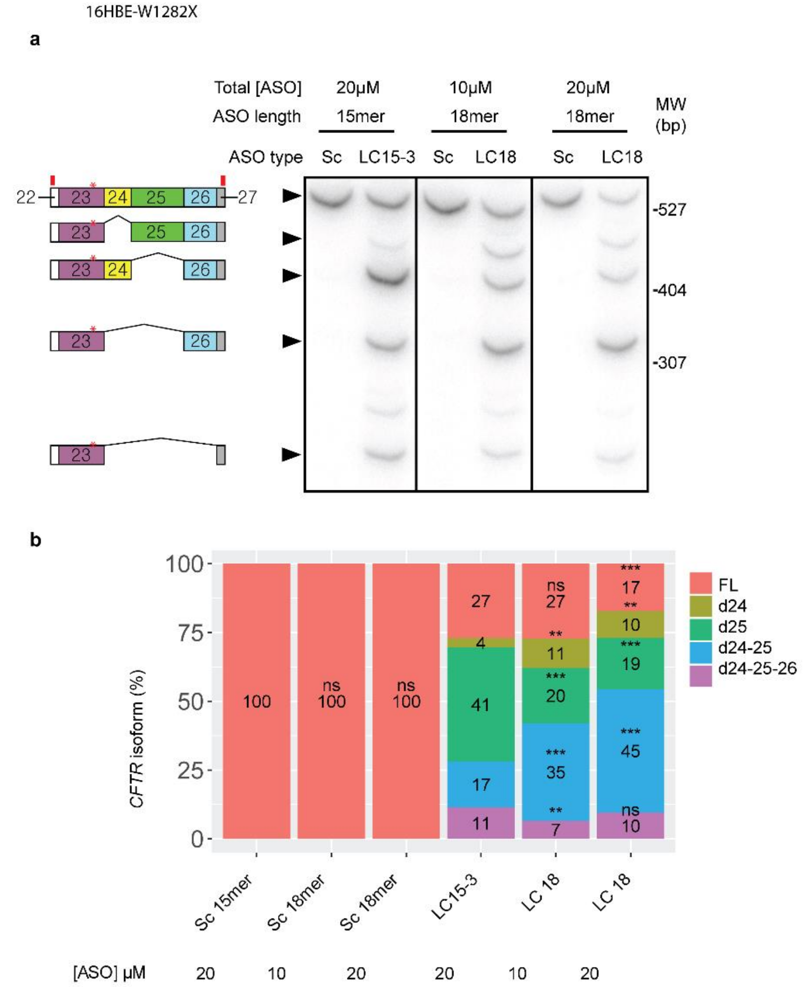

Supplementary Figure 9. *CFTR* splicing in 16HBE-W1282X cells treated with 15mer or 18mer ASO cocktails by free-uptake.

a. Representative RT-PCR results of *CFTR* mRNA splicing patterns in 16HBE-W1282X cells treated with LC15-3 or LC18. Primers targeting the exons 22 and 27 were used to measure PSI. The results have been replicated in at least 3 independent biological samples. All samples were run on the same gel, but lanes were reordered for clarity. **b.** Mean PSI of each *CFTR* isoform in (a) (n=3 independent transfections, n.s. $P>0.05$, **$P<0.01$, ***$P<0.001$, one-way ANOVA with Dunnett's post-test, versus each isoform in Sc15 or LC15-3). Abbreviations as in Figure 1 and 2. Data are represented as mean values of independent biological replicates. Source data are

provided as a Source Data file.

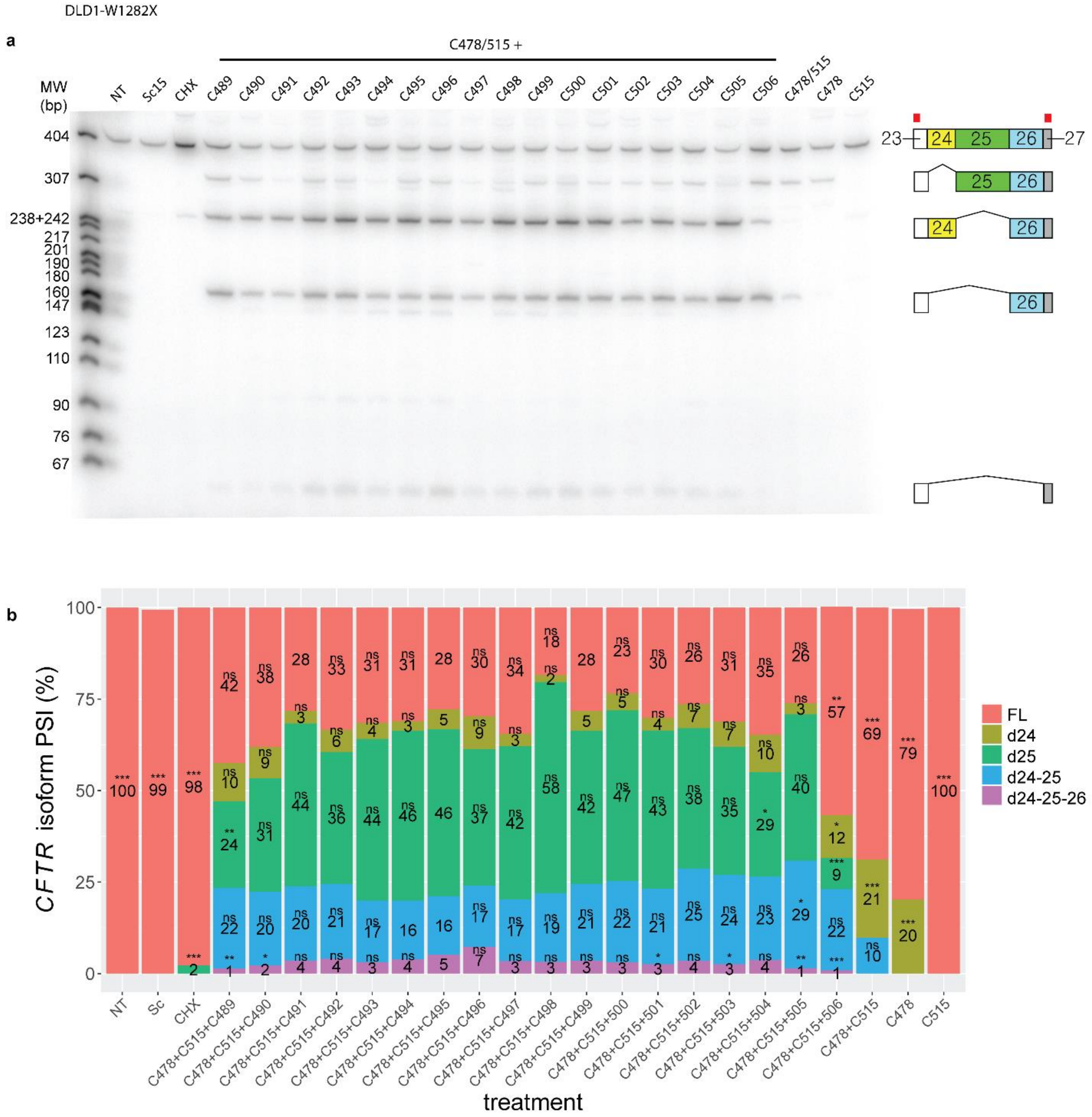

Supplementary Figure 10. ***CFTR*** **splicing in DLD1-W1282X cells transfected with 15mer ASO cocktails.**

a. Representative RT-PCR results of *CFTR* mRNA splicing patterns in DLD1-W1282X cells treated with various 15mer lead cocktails. Primers targeting the exons 23 and 27 were used to measure PSI. The results have been replicated in at least 3 independent biological samples. **b.**

Mean PSI of each *CFTR* isoform in DLD1-W1282X cells in (b) (n=3 independent transfections, n.s. P>0.05, *P<0.05, **P<0.01, ***P<0.001, one-way ANOVA with Dunnett's post-test, versus each isoform in C478:C495:C515=40:40:40 nM). Data are represented as mean values of independent biological replicates. Abbreviations as in Figures 1 and 2. Source data are provided as a Source Data file.

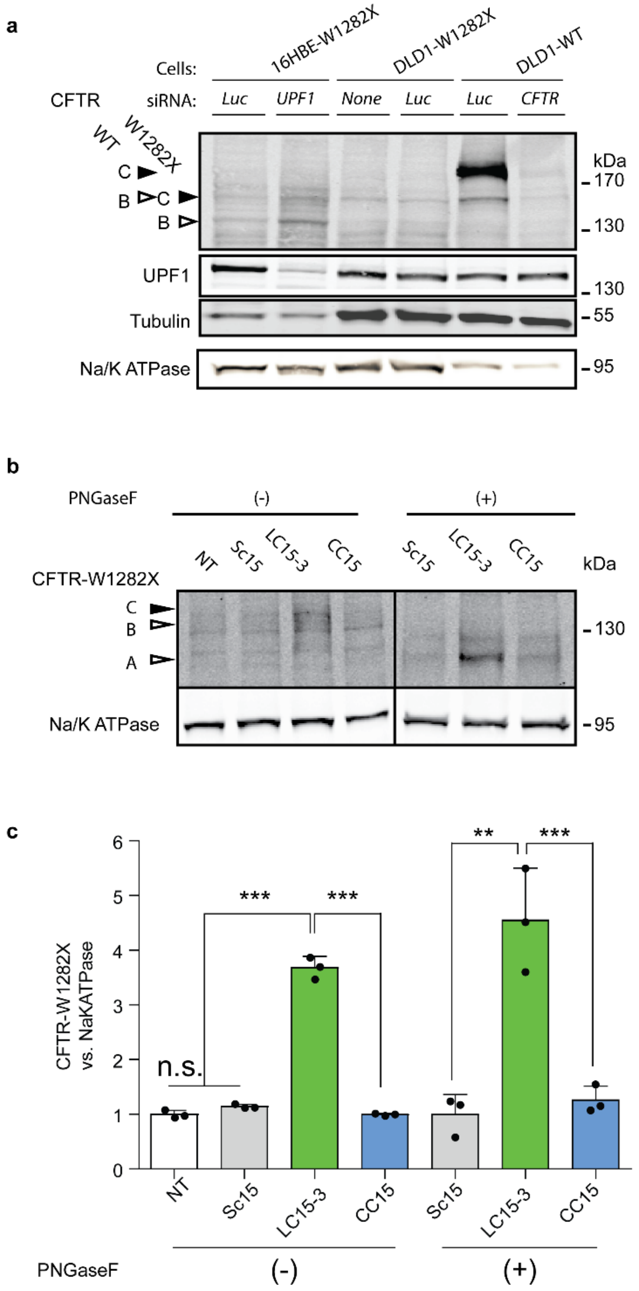

Supplementary Figure 11. The EJC-targeting ASO cocktail increases CFTR-W1282X protein.

a. CFTR antibody detects C and B bands of CFTR-WT and CFTR-W1282X. **b.** Western blot of complex glycosylated (C band), core-glycosylated (B-band), and non-glycosylated (A-band) of CFTR in 16HBE-W1282X cells transfected with 120 nM lead ASO cocktail LC15-3 (C478-494-

C515). PNGase F was used to deglycosylate proteins in the cell extracts. Sc15 and CC15 were used as negative controls. All samples were run on the same gel, but lanes were reordered for clarity. **c.** Quantification of total CFTR and deglycosylated CFTR protein in (b). Data are represented as mean values ± SD. All data points represent independent biological replicates. (With or without PNGaseF treatment: n=3 independent transfections, n.s. $P>0.05$, **$P<0.05$, ***$P<0.001$, one-way ANOVA with Tukey's post-test). Na^+/K^+ ATPase served as an internal reference. Western blot images are cropped images from the same SDS-PAGE gel. Source data are provided as a Source Data file.

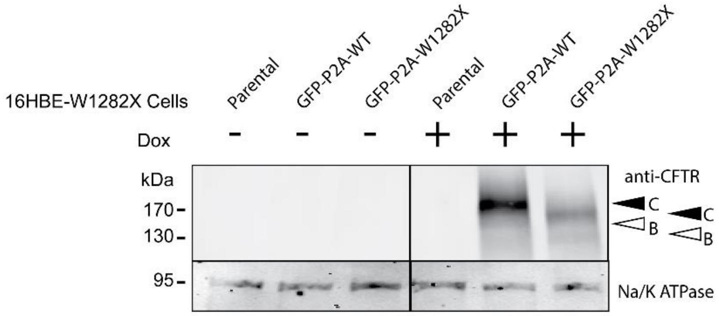

Supplementary Figure 12. Induction of GFP-P2A-CFTR expression by doxycycline.

Western blot of CFTR in parental 16HBEge-W1282X,16HBEge-GFP-P2A-WT, and 16HBEge-GFP-P2A-W1282X cells with or without 2μg/mL doxycycline treatment. All samples were run on the same gel, but lanes were reordered for clarity. The results were reproduced using at least one other independent biological replicate. Closed arrowhead: C-band. Open arrowhead: B-band. Dox=Doxycycline. Images are cropped from the same Western blot membrane. Source data are provided as a Source Data file.

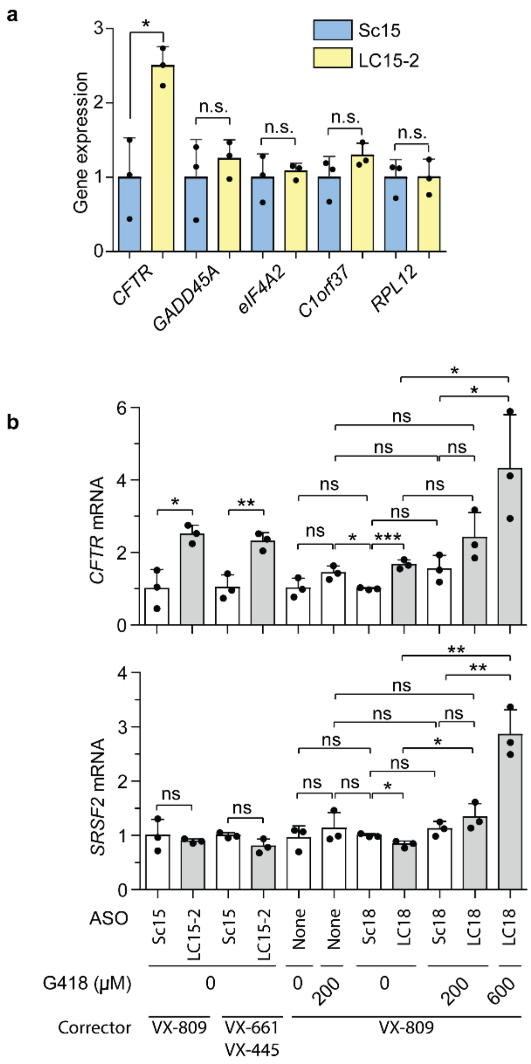

Supplementary Figure 13. NMD-targeted mRNA in 16HBE-W1282X cells after Ussing chamber assay.

a. The levels of *CFTR-W1282X* and endogenous NMD-sensitive mRNAs in 16HBE-W1282X cells that had undergone Ussing chamber assay after Sc15 or LC15-2 and VX-809 treatment in Figure 5a. **b.** The levels of *CFTR-W1282X* and NMD-sensitive *SRSF2* mRNA in 16HBE-W1282X cells in Figure 5a. *CFTR* mRNA levels were measured using forward and reverse primers targeting exon 22 and 23, respectively. *RPL32* was used as an internal reference. Data

are represented as mean values ± SD. All data points represent independent biological replicates. $n=3$, n.s. $P>0.05$, *$P<0.05$, **$P<0.01$, ***$P<0.001$, two-tailed Student’s t-test. Source data are provided as a Source Data file.

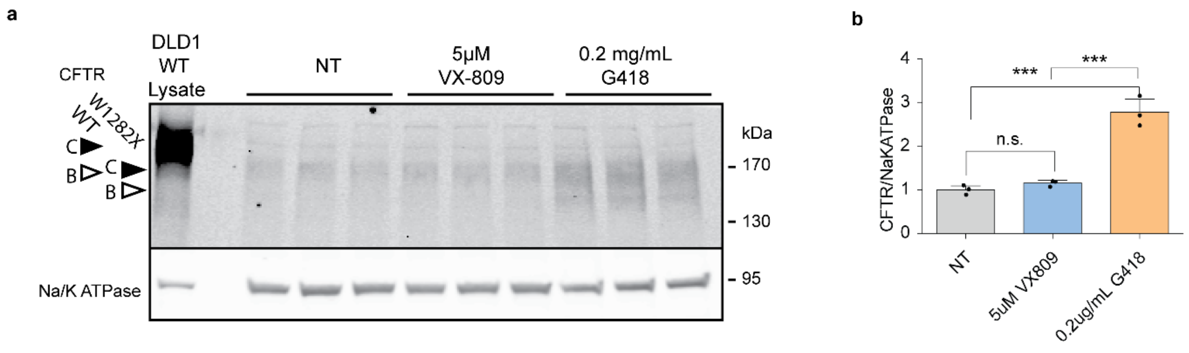

Supplementary Figure 14. G418 increases CFTR-W1282X protein in 16HBE-W1282X cells.

a. Western blot of 16HBE-W1282X cells treated with VX-809 or G418. **b.** Quantification of CFTR-W1282X proteins in (a). Data are represented as mean values ± SD. All data points represent independent biological replicates. (n=3 independent treatments, n.s. P>0.05, ***P<0.001, versus lead ASO cocktail, one-way ANOVA with Tukey's post-test). Na^+/K^+ ATPase served as an internal reference. Western blot images are cropped images from the same SDS-PAGE gel. Source data are provided as a Source Data file.

Supplementary Tables

Supplementary Table 1. ASOs

Cells highlighted in yellow show ASOs used for the lead ASO cocktails.		
ASO type	ASO Name	Sequence
EJC-targeting candidate	C470	CTTGATCACTCCACT
EJC-targeting candidate	C471	TCTTGATCACTCCAC
EJC-targeting candidate	C472	TTCTTGATCACTCCA
EJC-targeting candidate	C473	TTTCTTGATCACTCC
EJC-targeting candidate	C474	ATTTCTTGATCACTC
EJC-targeting candidate	C475	TATTTCTTGATCACT
EJC-targeting candidate	C476	ATATTTCTTGATCAC
EJC-targeting candidate	C477	CATATTTCTTGATCA
EJC-targeting candidate	C478	CCATATTTCTTGATC
EJC-targeting candidate	C479	TCCATATTTCTTGAT
EJC-targeting candidate	C480	TTCCATATTTCTTGA
EJC-targeting candidate	C481	TTTCCATATTTCTTG
EJC-targeting candidate	C482	CTTTCCATATTTCTT

EJC-targeting candidate	C483	ACTTTCCATATTTCT
EJC-targeting candidate	C484	AACTTTCCATATTTC
EJC-targeting candidate	C485	CAACTTTCCATATTT
EJC-targeting candidate	C486	GCAACTTTCCATATT
EJC-targeting candidate	C487	TGCAACTTTCCATAT
EJC-targeting candidate	C488	CTGCAACTTTCCATA
EJC-targeting candidate	C489	TTCATCAAGCAGCAA
EJC-targeting candidate	C490	GTTCATCAAGCAGCA
EJC-targeting candidate	C491	GGTTCATCAAGCAGC
EJC-targeting candidate	C492	GGGTTCATCAAGCAG
EJC-targeting candidate	C493	TGGGTTCATCAAGCA
EJC-targeting candidate	C494	CTGGGTTCATCAAGC
EJC-targeting candidate	C495	ACTGGGTTCATCAAG
EJC-targeting candidate	C496	CACTGGGTTCATCAA
EJC-targeting candidate	C497	GCACTGGGTTCATCA
EJC-targeting candidate	C498	AGCACTGGGTTCATC
EJC-targeting candidate	C499	GAGCACTGGGTTCAT

EJC-targeting candidate	C500	TGAGCACTGGGTTCA
EJC-targeting candidate	C501	ATGAGCACTGGGTTC
EJC-targeting candidate	C502	AATGAGCACTGGGTT
EJC-targeting candidate	C503	AAATGAGCACTGGGT
EJC-targeting candidate	C504	CAAATGAGCACTGGG
EJC-targeting candidate	C505	CCAAATGAGCACTGG
EJC-targeting candidate	C506	TCCAAATGAGCACTG
EJC-targeting candidate	C507	ATCCAAATGAGCACT
EJC-targeting candidate	C508	TTGCTTCTATCCTGT
EJC-targeting candidate	C509	ATTGCTTCTATCCTG
EJC-targeting candidate	C510	CATTGCTTCTATCCT
EJC-targeting candidate	C511	GCATTGCTTCTATCC
EJC-targeting candidate	C512	AGCATTGCTTCTATC
EJC-targeting candidate	C513	CAGCATTGCTTCTAT
EJC-targeting candidate	C514	CCAGCATTGCTTCTA
EJC-targeting candidate	C515	TCCAGCATTGCTTCT
EJC-targeting candidate	C516	TTCCAGCATTGCTTC

EJC-targeting candidate	C517	ATTCCAGCATTGCTT
EJC-targeting candidate	C518	CATTCCAGCATTGCT
EJC-targeting candidate	C519	GCATTCCAGCATTGC
EJC-targeting candidate	C520	GGCATTCCAGCATTG
EJC-targeting candidate	C521	TGGCATTCCAGCATT
EJC-targeting candidate	C522	TTGGCATTCCAGCAT
EJC-targeting candidate	C523	GTTGGCATTCCAGCA
EJC-targeting candidate	C524	TGTTGGCATTCCAGC
EJC-targeting candidate	C525	TTGTTGGCATTCCAG
EJC-targeting candidate	C526	ATTGTTGGCATTCCA
Scramble 15mer	Scramble control based on C494	CACGCTACTTGATGC
EJC 18mer	C24-18m	TTCCATATTTCTTGATCA
EJC 18mer	C25-18m	ACTGGGTTCATCAAGCAG
EJC 18mer	C26-18m	TTCCAGCATTGCTTCTAT
Scramble 18mer	Scramble control based on C25	ACAGGCTTCTTCATGCAC
Control	H24	CTCAGGATCCACGTG
Control	H26	CAGGATCCACGTGCA

Control	H27	AGGATCCACGTGCAG

Supplementary Table 2. ASO cocktails

Lead cocktail 15mer 1 (LC15-1)	C478, C495, C515
Lead cocktail 15mer 2 (LC15-2)	C478, C494, C514
Lead cocktail 15mer 3 (LC15-3)	C478, C494, C515
Lead cocktail 18mer (LC18)	C24-18m, C25-18m, C26-18m
Control Cocktail (CC)	C488, C506, C526

Supplementary Table 3. sgRNA and W1282X **repair template sequences**

CFTR sgRNA Sense strand	5'-CACCGCAATAACTTTGCAACAGTGG-3'
CFTR sgRNA antisense strand	5'-AAACCCACTGTTGCAAAGTTATTGC-3'
W1282X repair template	5’-AACACTGAAGGAGAAATCCAGATCGATGGTGTGTCTTGGGATTCAATAACTTTGCAACAGTGAAGAAAAGCCTTTGGAGTGATACCACAGGTGAGCAAAAGGACTTAGCCAGAAAAAAGG-3’

Supplementary Table 4. siRNA

Source	Target gene	Sequence	Source
Custom-sense	UPF1	GAUGCAGUUCCGCUCCAUU	Sigma
Custom-antisense	UPF1	AAUGGAGCGGAACUGCAUC	Sigma
Predesigned	CFTR	SASI_Hs02_00302648	Sigma

Supplementary Table 5. Primers for RT-PCR and RT-qPCR

Target gene	primer	seq	source
NMD-sensitive GADD45A	GADD45A_NMD_F	GAGCTCCTGCTCTTGGAGAC	*Mabin et al., 2018*
NMD-sensitive GADD45A	GADD45A_NMD_R	GCAGGATCCTTCCATTGAGA	*Mabin et al., 2018*
NMD-sensitive eIF4A2	eIF4A2_NMD_F	AGGGTCAAGTCGTGTTCTGATC	*Mabin et al., 2018*
NMD-sensitive eIF4A2	eIF4A2_NMD_R	ACCAACTGCTGCTATCGACTC	*Mabin et al., 2018*
NMD-sensitive SF3B1	SF3B1_NMD_F	AATTTCCCCAGAGCGTCTTG	*Mabin et al., 2018*
NMD-sensitive SF3B1	SF3B1_NMD_R	TTCGTGCCTTTGTCTCCATC	*Mabin et al., 2018*
NMD-sensitive C1orf37	C1orf37_NMD_F	TTGCTGCTCGAATCTCCAAG	*Mabin et al., 2018*
NMD-sensitive C1orf37	C1orf37_NMD_R	ACTTCTGCTGCCATCACAAC	*Mabin et al., 2018*

NMD-sensitive SRSF2	SRSF2_NMD_F	CCTCTTAAGAAAATGCTGCGGTCTC	Lareau et al., 2007
NMD-sensitive SRSF2	SRSF2_NMD_R	ATCAGCCAAATCAGTTAAAATCTGC	Lareau et al., 2007
NMD-sensitive SRSF4	SRSF4_NMD_F	GGATCTGAAGAACGGTCTGTTATGT	Lareau et al., 2007
NMD-sensitive SRSF4	SRSF4_NMD_R	TCACTCGTCTTTTGGTTCCCATTAG	Lareau et al., 2007
NMD-sensitive RPL12	RPL12_NMD_F	CTGGGCCTTAGCTTCTTCAC	Lareau et al., 2007
NMD-sensitive RPL12	RPL12_NMD_R	AAGTGGCACCGACTTCACCT	*Mabin et al., 2018*
CFTR-exon22	CFTR22F	CAATAAGTCCTGGCCAGAGG	
CFTR-exon25	CFTR25F	GAAGATCTTGCTGCTTGATG	
CFTR-exon26	CFTR26F	GCACAGTAATTCTCTGTGAACACAGG	
CFTR-exon27	CFTR27R	TCCTCTCGTTCAGCAGTTTCTGG	
CFTR-exon23	CFTR23F	TTGCAACAGtgaAGGAAAGCC	
CFTR-exon23	CFTR23R	AAGGCTTTCCTtcaCTGTTGC	
CFTR-exon22-splicing	CFTR-22F-splice	GCGATCTGTGAGCCGAGTC	

CFTR-exon24-splicing	CFTR-24R-splice	CTTGATCACTCCACTGTTCATAGGGATC	
ACTB	ACTB_F	AGAGCTACGAGCTGCCTGAC	
ACTB	ACTB_R	AGCACTGTGTTGGCGTACAG	
RPL32	RPL32_F	AGAGGCATTGACAACAGGGTT	
RPL32	RPL32_R	GTGAGCGATCTCGGCACAG	
HPRT	HPRT_F	TGACCAGTCAACAGGGGACA	
HPRT	HPRT_R	TGCCTGACCAAGGAAAGCAA	
