# Supplementary material for: Gene-specific nonsense-mediated mRNA decay targeting for cystic fibrosis therapy
Source: Nat Commun. 2022 May 27;13:2978. doi: 10.1038/s41467-022-30668-y (PMC9142507; doi:10.1038/s41467-022-30668-y)
Supplement: Supplementary file 3 — Description of Additional Supplementary Files [file 41467_2022_30668_MOESM3_ESM.pdf]

Description of Additional Supplementary Files

File Name: Supplementary Data 1
Description: Exact p values in the figures.
