# Supplementary material for: Gene-specific nonsense-mediated mRNA decay targeting for cystic fibrosis therapy
Source: Nat Commun. 2022 May 27;13:2978. doi: 10.1038/s41467-022-30668-y (PMC9142507; doi:10.1038/s41467-022-30668-y)
Supplement: Supplementary file 5 — Reporting Summary [file 41467_2022_30668_MOESM5_ESM.pdf]

nature portfolio

Corresponding author(s): Adrian R. Krainer

Last updated by author(s): Apr 12, 2022

Reporting Summary

Nature Portfolio wishes to improve the reproducibility of the work that we publish. This form provides structure for consistency and transparency in reporting. For further information on Nature Portfolio policies, see our Editorial Policies and the Editorial Policy Checklist.

Statistics

For all statistical analyses, confirm that the following items are present in the figure legend, table legend, main text, or Methods section.

n/a	Confirmed	
☐	☒	The exact sample size (*n*) for each experimental group/condition, given as a discrete number and unit of measurement
☐	☒	A statement on whether measurements were taken from distinct samples or whether the same sample was measured repeatedly
☐	☒	The statistical test(s) used AND whether they are one- or two-sided<br>*Only common tests should be described solely by name; describe more complex techniques in the Methods section.*
☐	☒	A description of all covariates tested
☐	☒	A description of any assumptions or corrections, such as tests of normality and adjustment for multiple comparisons
☐	☒	A full description of the statistical parameters including central tendency (e.g. means) or other basic estimates (e.g. regression coefficient) AND variation (e.g. standard deviation) or associated estimates of uncertainty (e.g. confidence intervals)
☐	☒	For null hypothesis testing, the test statistic (e.g. *F*, *t*, *r*) with confidence intervals, effect sizes, degrees of freedom and *P* value noted<br>*Give P values as exact values whenever suitable.*
☒	☐	For Bayesian analysis, information on the choice of priors and Markov chain Monte Carlo settings
☒	☐	For hierarchical and complex designs, identification of the appropriate level for tests and full reporting of outcomes
☒	☐	Estimates of effect sizes (e.g. Cohen's *d*, Pearson's *r*), indicating how they were calculated

Our web collection on statistics for biologists contains articles on many of the points above.

Software and code

Policy information about availability of computer code

Data collection	No codes were used to collect data in this project.
Data analysis	GraphPad Prism 5 was used for all statistical analysis.<br>Ussing chamber assay data acquisition performed using ACQUIRE & ANALYZE Revision II (Physiologic Instruments).<br>Western blots were imaged and quantified using an Odyssey Infrared Imaging System (LI-COR).<br>Radioactive RT-PCR data was collected by Typhoon FLA7000 phosphorimager and quantitated using MultiGauge v2.3 software (Fujifilm).<br>qRT-PCR data was collected by QuantStudio Real-Time PCR Software v1.3.<br>A Revolve microscope was used for imaging, and the images were acquired by ECHO Pro v6.0.1 software (Revolve, ECHO, San Diego, CA, United States).<br>GFP-intensity analysis from the images was performed with ImageJ2 software (version 2.2.0).

For manuscripts utilizing custom algorithms or software that are central to the research but not yet described in published literature, software must be made available to editors and reviewers. We strongly encourage code deposition in a community repository (e.g. GitHub). See the Nature Portfolio guidelines for submitting code & software for further information.

Data

Policy information about availability of data

All manuscripts must include a data availability statement. This statement should provide the following information, where applicable:

- Accession codes, unique identifiers, or web links for publicly available datasets
- A description of any restrictions on data availability
- For clinical datasets or third party data, please ensure that the statement adheres to our policy

All data generated or analyzed during this study are included in this published article (and its supplementary information files). Source data are provided with this paper.

Field-specific reporting

Please select the one below that is the best fit for your research. If you are not sure, read the appropriate sections before making your selection.

☒ Life sciences ☐ Behavioural & social sciences ☐ Ecological, evolutionary & environmental sciences

For a reference copy of the document with all sections, see nature.com/documents/nr-reporting-summary-flat.pdf

Life sciences study design

All studies must disclose on these points even when the disclosure is negative.

Sample size	Sample size was determined based on Authors' experience and preliminary experiments as well as similar experiments in the published literature. The number of replicates for each experiment is provided in the manuscript. At least two biological replicates were used to ensure reproducibility. The conclusions were made based on multiple complementary approaches.<br>For quantitation and analysis of GFP signal in 16HBEge-GFP-P2A-WT/W1282X cells, more than 200 cells were analyzed in each experiment.
Data exclusions	No data was excluded from the analysis
Replication	Each experiment was performed with at least two independent biological replicates.
Randomization	Randomization was not performed as it is not applicable in in vitro experiments with immortalized cell lines, where all samples within the biological experiments are derived from the same batch of cells.
Blinding	Blinding was not used except for quantitation and analysis of GFP signals in 16HBEge-GFP-P2A-WT/W1282X via fluorescent microscopy. For the quantitation and analysis of 16HBEge-GFP-P2A-WT/W1282X cells, cells were counted and rated in a blinded way by independent, blinded investigators. Data was acquired using imaging setups that performed the measurement independent of the observer.

Reporting for specific materials, systems and methods

We require information from authors about some types of materials, experimental systems and methods used in many studies. Here, indicate whether each material, system or method listed is relevant to your study. If you are not sure if a list item applies to your research, read the appropriate section before selecting a response.

Materials & experimental systems

n/a	Involved in the study	
☐	☒	Antibodies
☐	☒	Eukaryotic cell lines
☒	☐	Palaeontology and archaeology
☒	☐	Animals and other organisms
☒	☐	Human research participants
☒	☐	Clinical data
☒	☐	Dual use research of concern

Methods

n/a	Involved in the study	
☒	☐	ChIP-seq
☒	☐	Flow cytometry
☒	☐	MRI-based neuroimaging

Antibodies

Antibodies used	Primary antibody:<br>anti-CFTR antibody UNC-596 (J. Riordan lab, University of North Carolina, Chapel Hill, NC), dilution 1:1000<br>anti-Na/K-ATPase antibody clone H-3 (Santa Cruz, sc-48345), dilution 1:10000<br>anti-UPF1 antibody D15G6 (Cell Signaling Technology #12040S), dilution 1:1000<br>anti-beta-Tubulin antibody (GenScript, Cat# A01203), dilution 1:3000

secondary antibody:
IRDye 800CW Goat anti-Mouse IgG Secondary Antibody (LI-COR, 926-32210), dilution 1:10000
IRDye 680RD Goat anti-Mouse IgG Secondary Antibody (LI-COR, 926-68070), dilution 1:10000

Validation

anti-CFTR antibody UNC-596 (J. Riordan lab, University of North Carolina, Chapel Hill, NC).
- authenticated and validated by cystic fibrosis foundation (https://cftrantibodies.web.unc.edu/).
-Antibody The specificity of the antibody was confirmed by knocking down CFTR in WT DLD1 cells (Supp Fig. 11A).
- LOT# 596TJ08252014

anti-Na/K-ATPase antibody (Santa Cruz, sc-48345)
-"Anti-ATP1A1/ATP1A2/ATP1A3 Antibody (H-3) is a mouse monoclonal IgG2b κ ATP1A1/ATP1A2/ATP1A3 antibody, cited in 59 publications, provided at 200 µg/ml" (https://www.scbt.com/p/na-k-atpase-alpha-antibody-h-3)
- LOT # G14115

UPF1 antibody D15G6 (Cell Signaling Technology #12040S)
- "Upf1 (D15G6) Rabbit mAb recognizes endogenous levels of total Upf1 protein" (https://www.cellsignal.com/datasheet.jsp?productId=12040&images=0&protocol=0)
- LOT # 1

anti-beta-Tubulin antibody (GenScript, Cat# A01203)
- "GenScript Rabbit Anti-β-Tubulin III Polyclonal Antibody detects endogenous levels of human, rat and mouse β-tubulin III protein." (https://www.genscript.com/product/documents?cat_no=A01203&catalogtype=Document-PROTOCOL)
-LOT #A117812

Eukaryotic cell lines

Policy information about cell lines

Cell line source(s)

T-REx-U2OS (life technologies, Cat #R712-07).
U2OS cells stably expressing NMD reporters - derived from parental T-REx-U2OS cells as described in the methods.
DLD1 WT (ATCC, CCL-221)
DLD1 W1282X - Derived from parental DLD1 WT, Genome edited by Kim et al.,
all 16HBEge cell lines CFF-16HBEge CFTR W1282X, F508del, G551D, G542X, or R1162X - cystic fibrosis foundation.

Authentication

T-REx-U2OS cells and DLD1 cells were obtained from ATCC, a certified commercial vendor, which authenticates these cell lines.
U2OS cells stably expressing the NMD reporters were authenticated by sequencing the plasmids used for transfection and RT-PCR of the NMD reporter after induction.
The DLD1-W1282X cells were authenticated by targeted sequencing of CFTR exon 23.
16HBEge cell lines authenticated by genome sequencing by cystic fibrosis foundation.

Mycoplasma contamination

Luminescence-based Mycoplasma detection kit (MycoAlert, Lonza, LT07-318)

Commonly misidentified lines
(See ICLAC register)

None
