# Supplementary figures and images for: Gene-specific nonsense-mediated mRNA decay targeting for cystic fibrosis therapy (part 2 of 2)
Source: Nat Commun. 2022 May 27;13:2978. doi: 10.1038/s41467-022-30668-y (PMC9142507; doi:10.1038/s41467-022-30668-y)

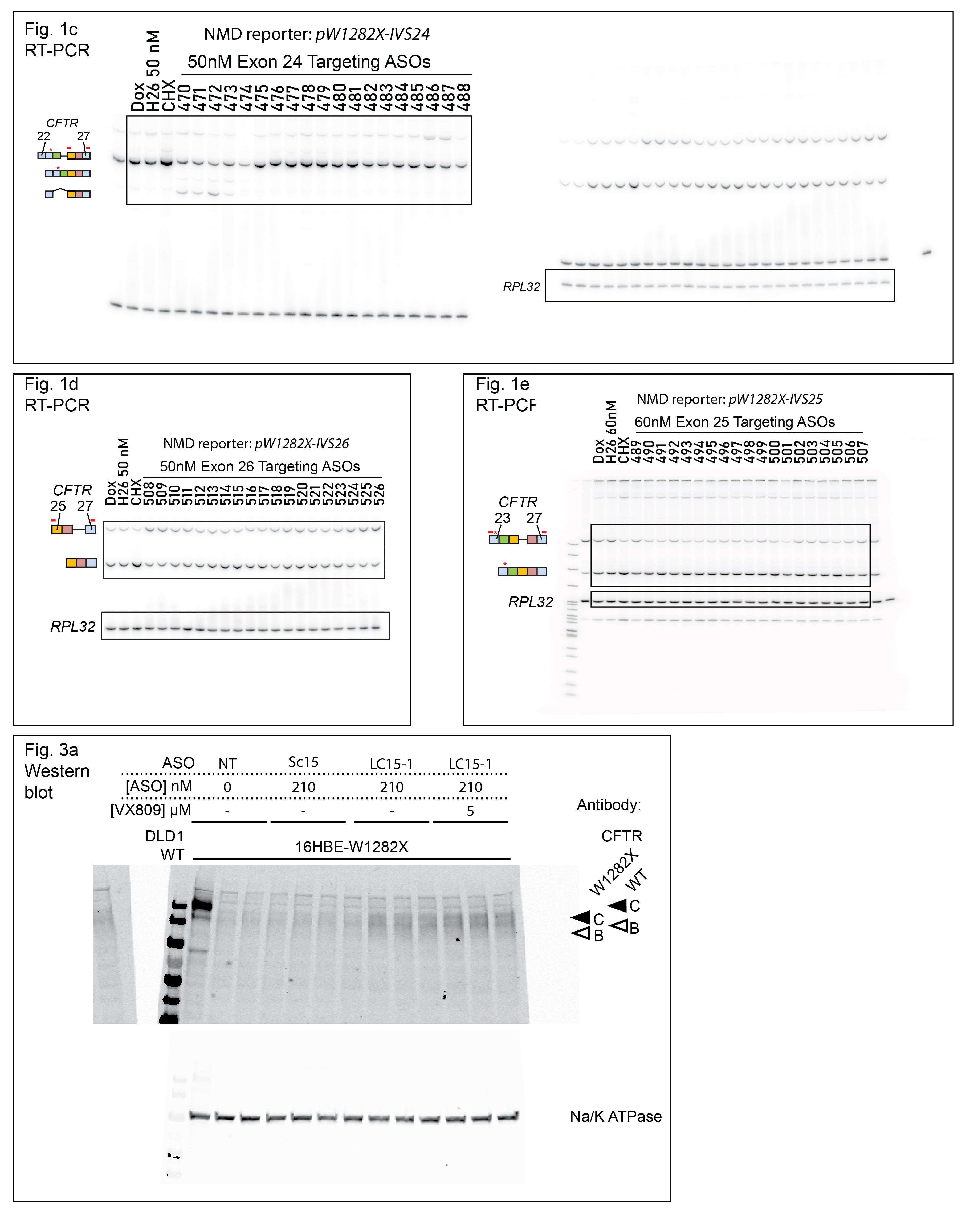

Supplement: Supplementary file 6 — Source Data [file 41467_2022_30668_MOESM6_ESM.zip › Source data, excel/original image summary_main fig.jpg]
