# Supplementary figures and images for: Gene-specific nonsense-mediated mRNA decay targeting for cystic fibrosis therapy (part 1 of 2)
Source: Nat Commun. 2022 May 27;13:2978. doi: 10.1038/s41467-022-30668-y (PMC9142507; doi:10.1038/s41467-022-30668-y)

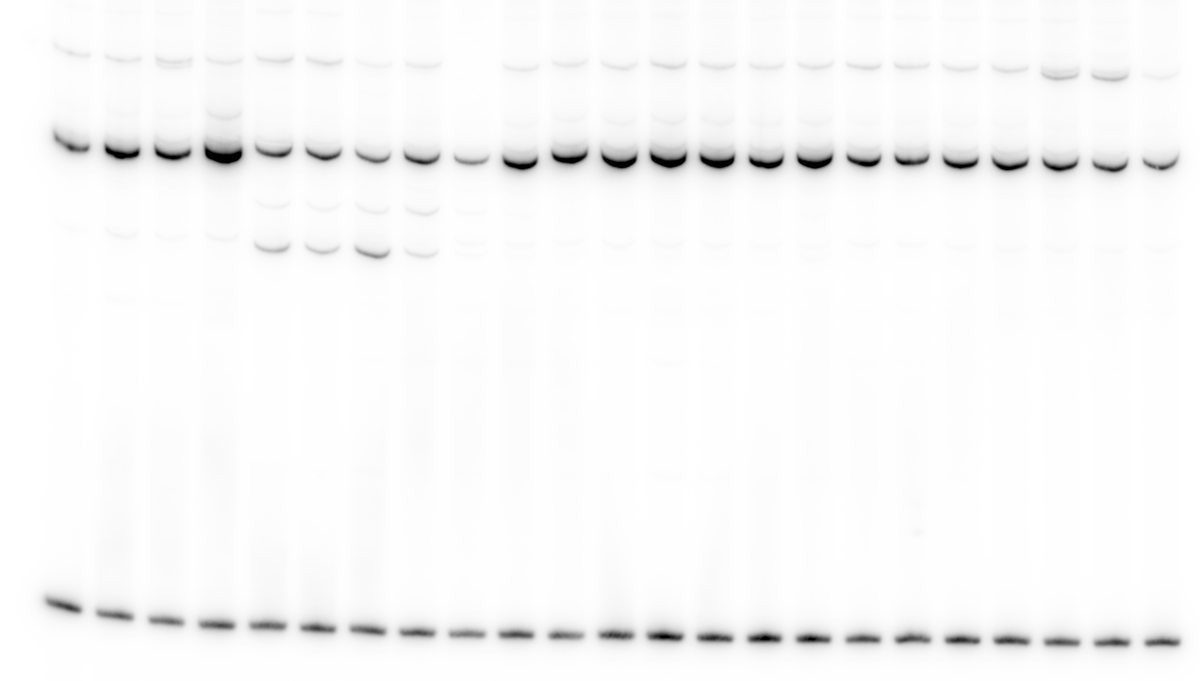

Supplement: Supplementary file 6 — Source Data [file 41467_2022_30668_MOESM6_ESM.zip › uncropped images/resized/fig.1c.tif]

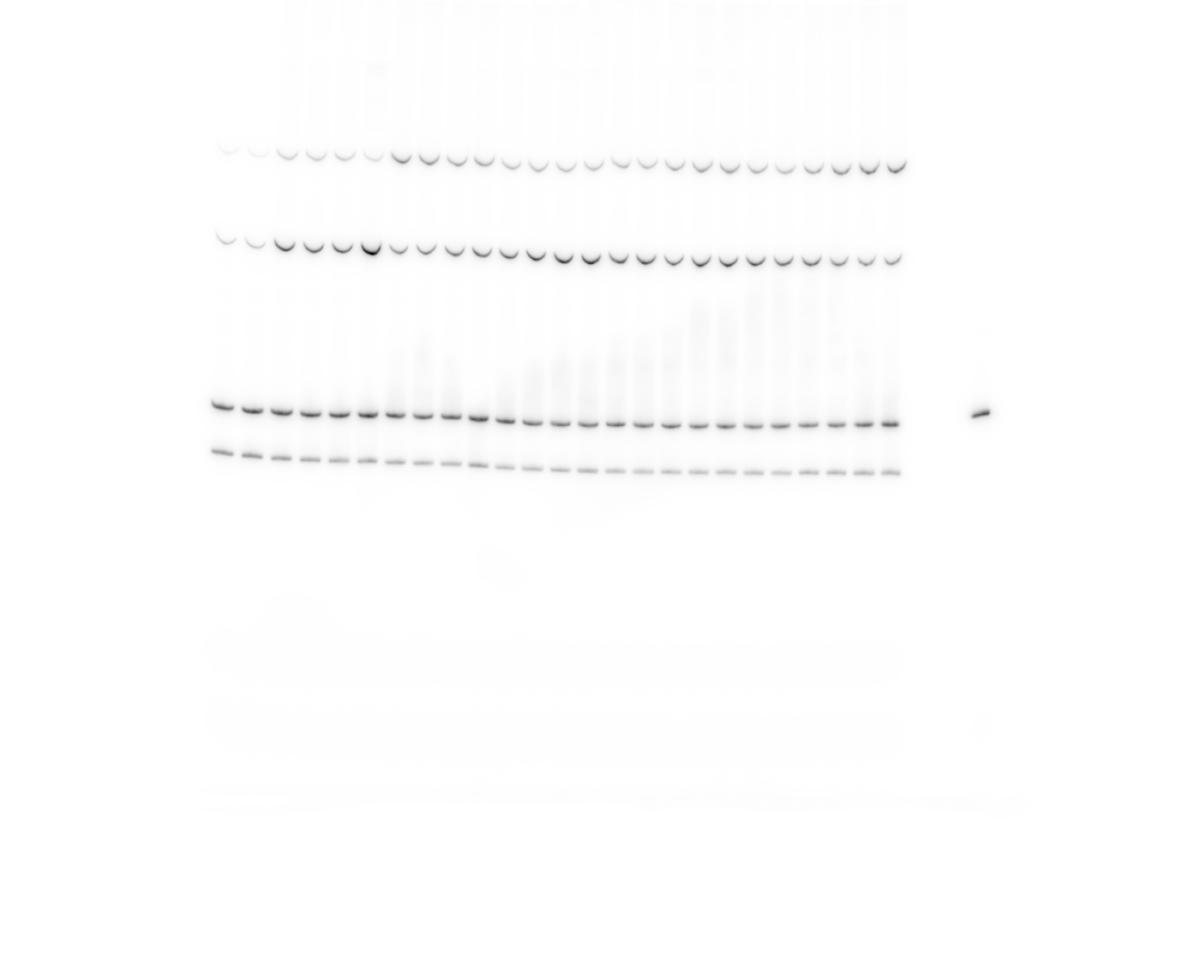

Supplement: Supplementary file 6 — Source Data [file 41467_2022_30668_MOESM6_ESM.zip › uncropped images/resized/fig.1d.tif]

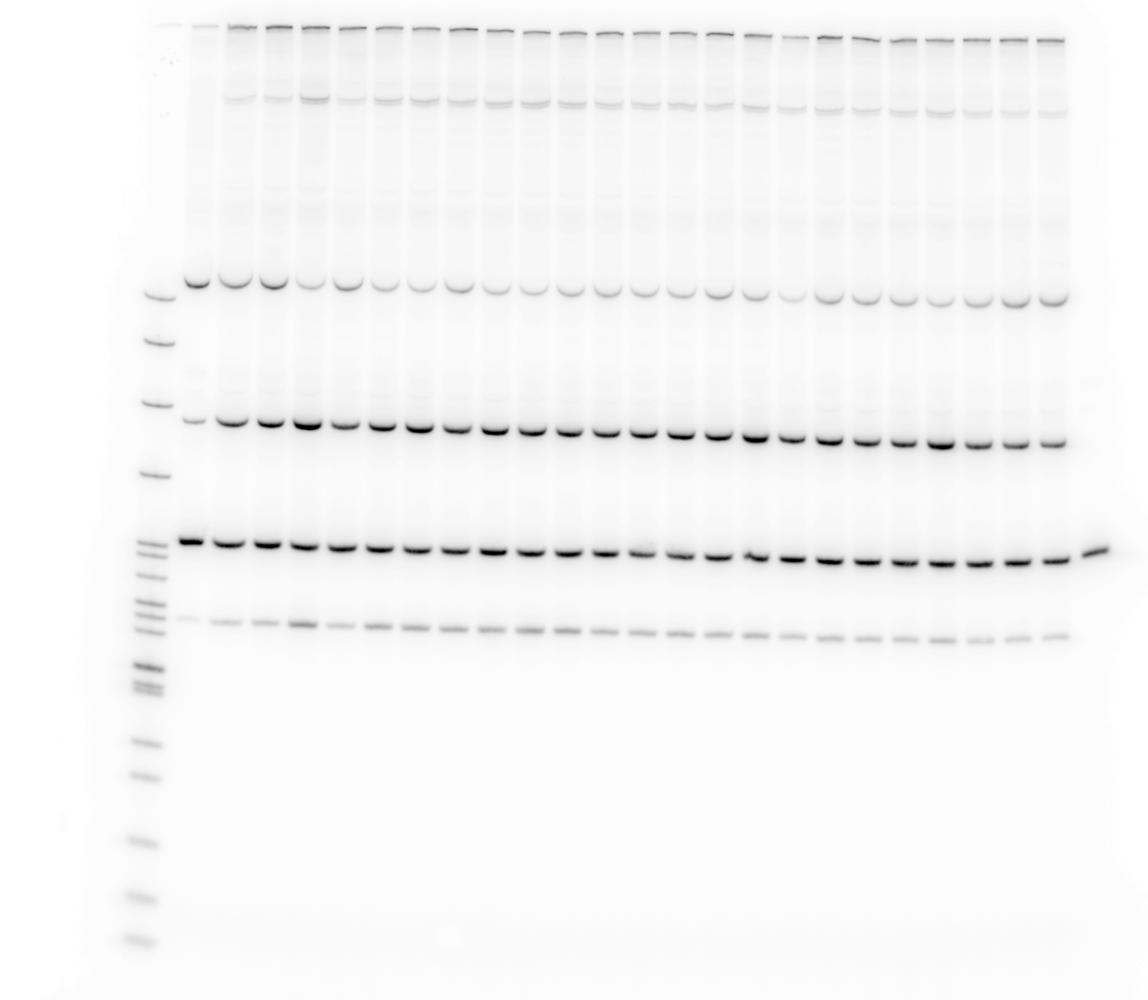

Supplement: Supplementary file 6 — Source Data [file 41467_2022_30668_MOESM6_ESM.zip › uncropped images/resized/fig.1e.tif]

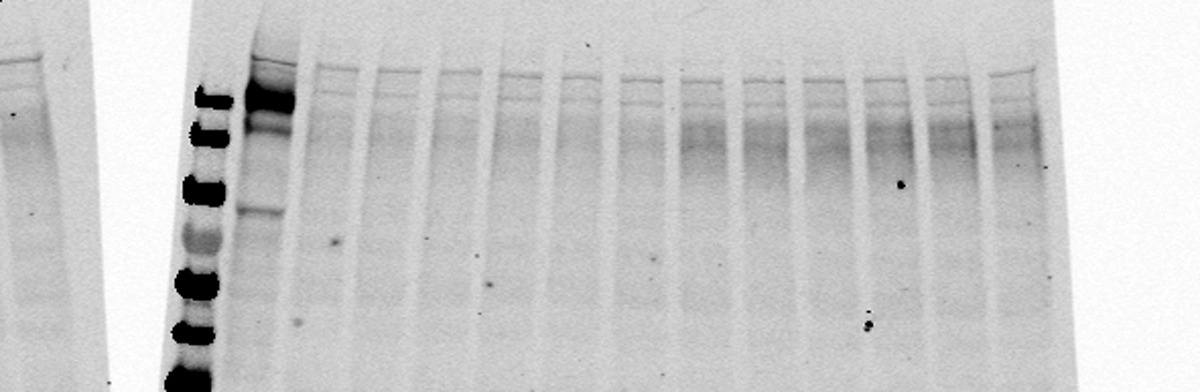

Supplement: Supplementary file 6 — Source Data [file 41467_2022_30668_MOESM6_ESM.zip › uncropped images/resized/fig.3a CFTR.tif]

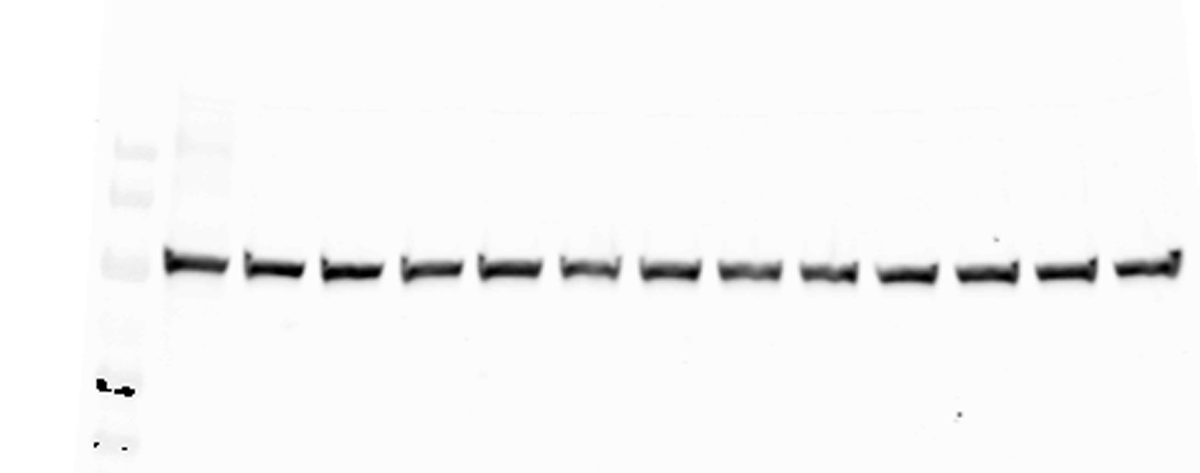

Supplement: Supplementary file 6 — Source Data [file 41467_2022_30668_MOESM6_ESM.zip › uncropped images/resized/fig.3a NAKATPase.tif]

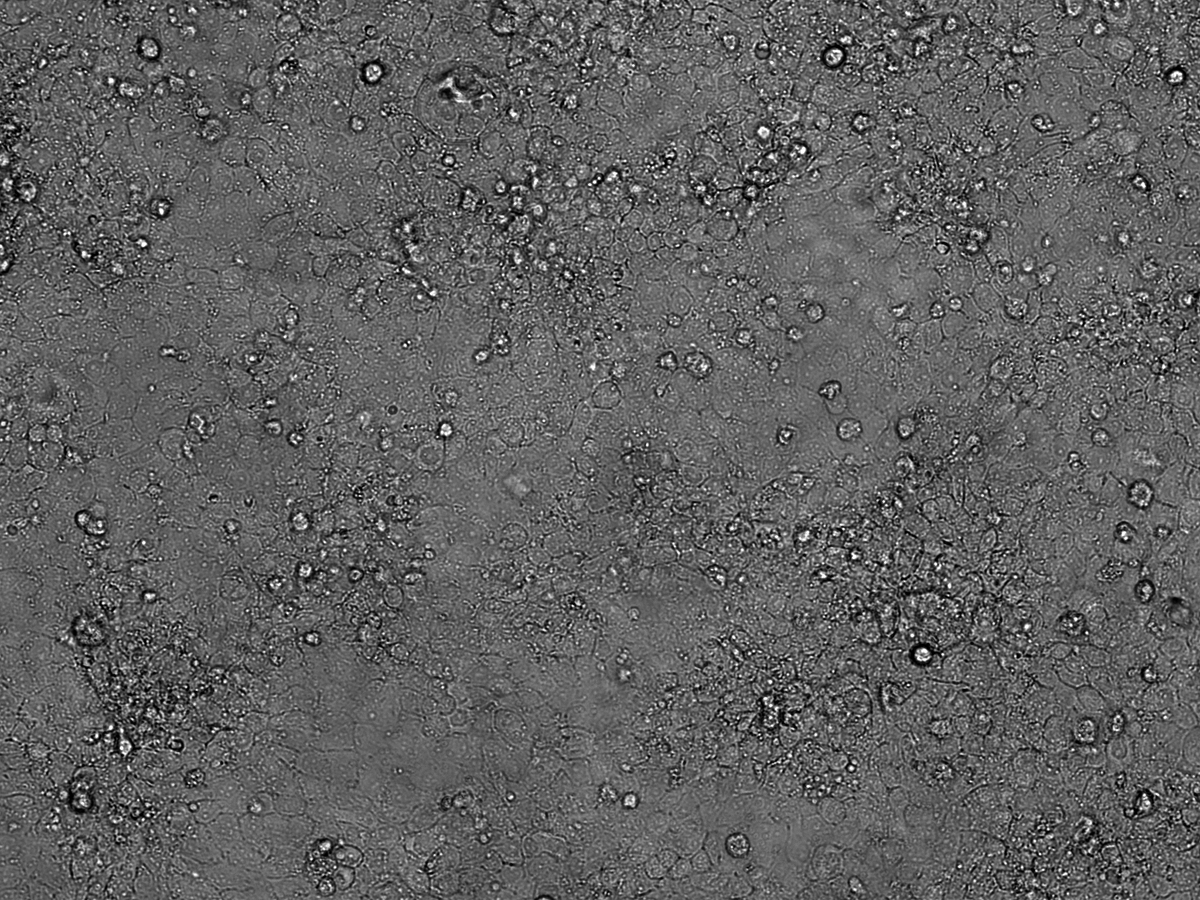

Supplement: Supplementary file 6 — Source Data [file 41467_2022_30668_MOESM6_ESM.zip › uncropped images/resized/fig.4b p2a-w1282x-dox brightfield.tif]

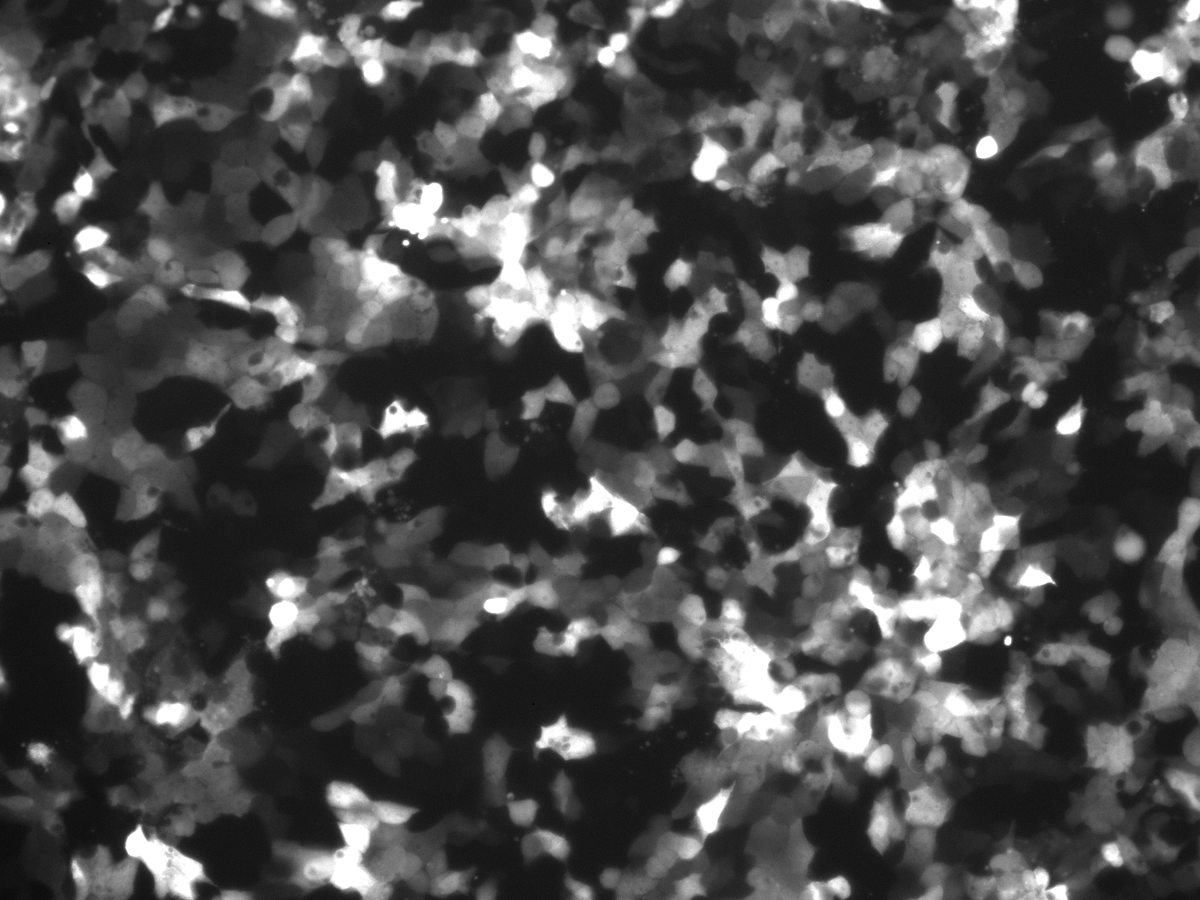

Supplement: Supplementary file 6 — Source Data [file 41467_2022_30668_MOESM6_ESM.zip › uncropped images/resized/fig.4b p2a-w1282x-dox gfp.tif]

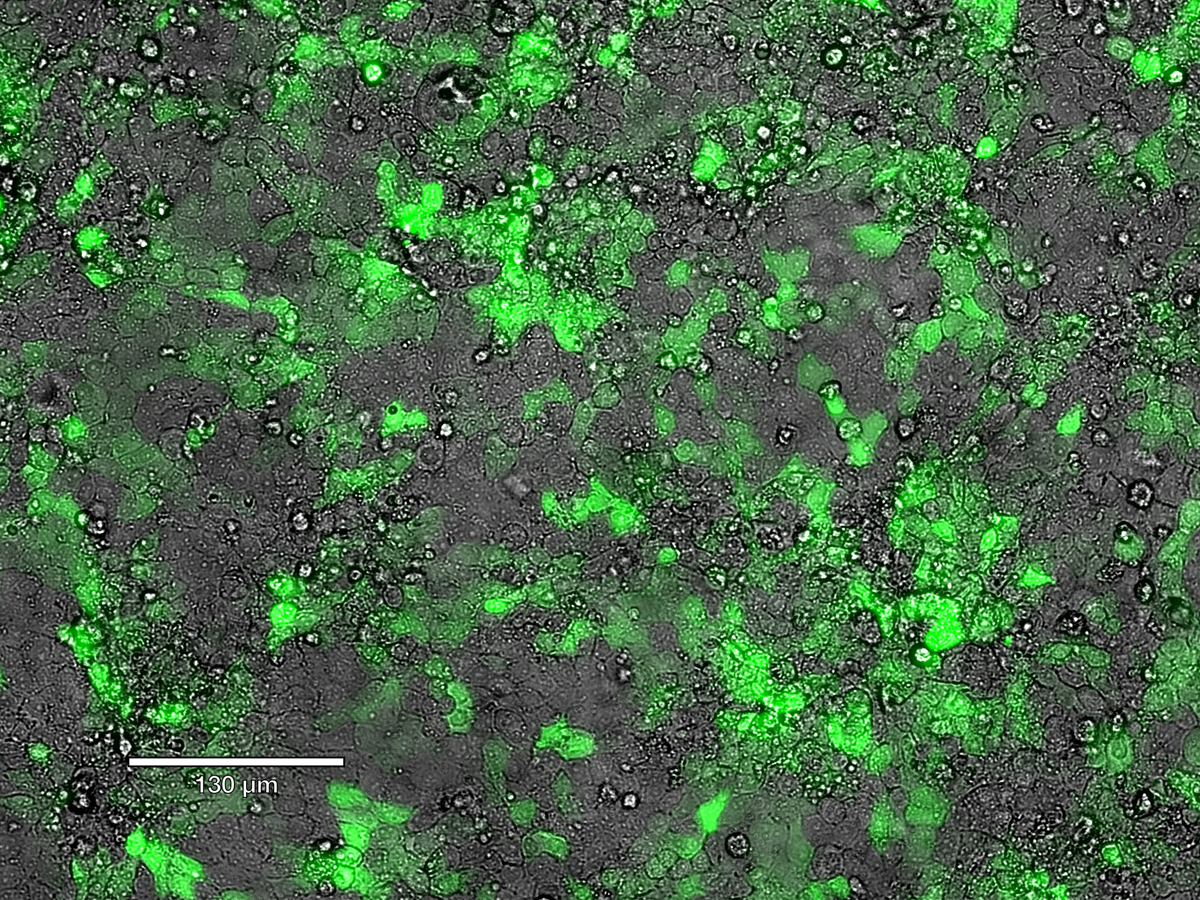

Supplement: Supplementary file 6 — Source Data [file 41467_2022_30668_MOESM6_ESM.zip › uncropped images/resized/fig.4b p2a-w1282x-dox merge.tif]

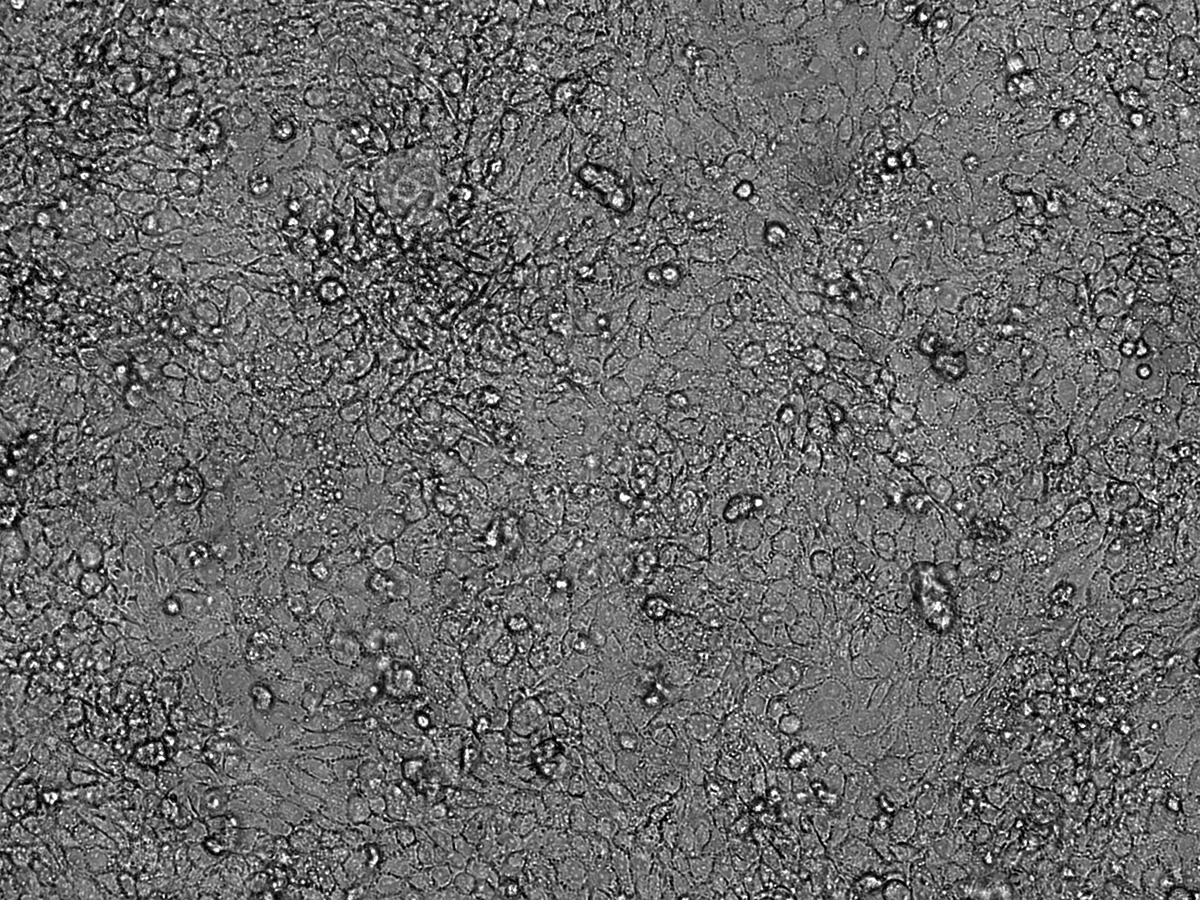

Supplement: Supplementary file 6 — Source Data [file 41467_2022_30668_MOESM6_ESM.zip › uncropped images/resized/fig.4b p2a-w1282x-nodox brightfield.tif]

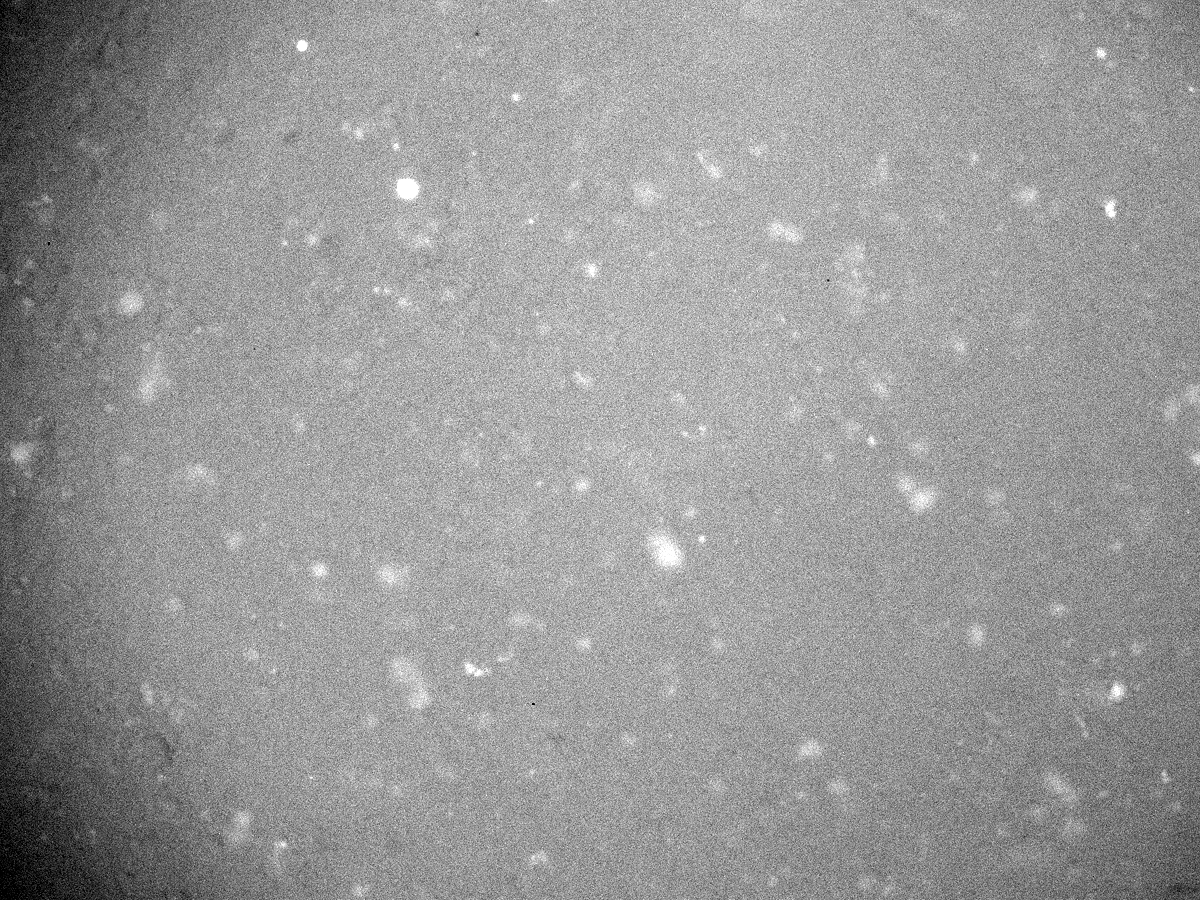

Supplement: Supplementary file 6 — Source Data [file 41467_2022_30668_MOESM6_ESM.zip › uncropped images/resized/fig.4b p2a-w1282x-nodox gfp.tif]

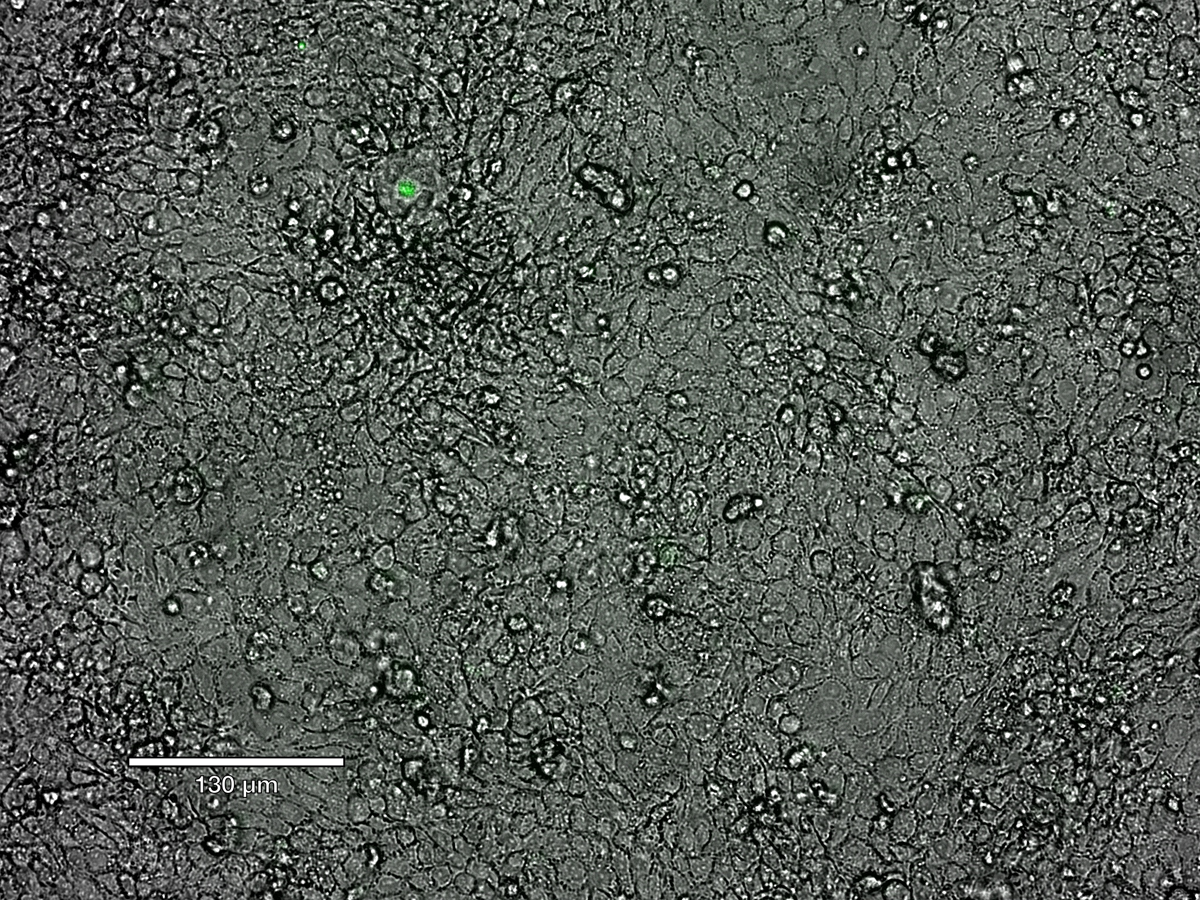

Supplement: Supplementary file 6 — Source Data [file 41467_2022_30668_MOESM6_ESM.zip › uncropped images/resized/fig.4b p2a-w1282x-nodox merge.tif]

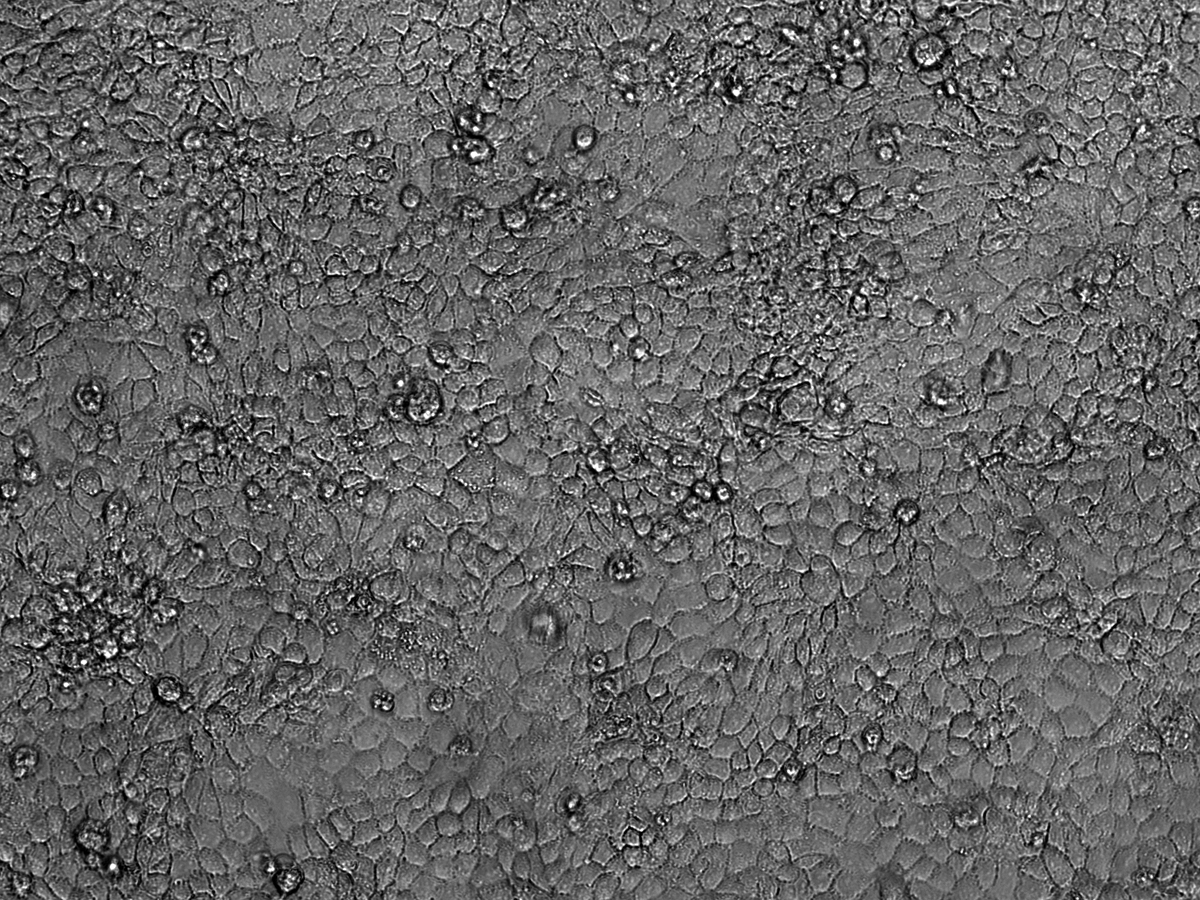

Supplement: Supplementary file 6 — Source Data [file 41467_2022_30668_MOESM6_ESM.zip › uncropped images/resized/fig.4b p2a-wt-dox brightfield.tif]

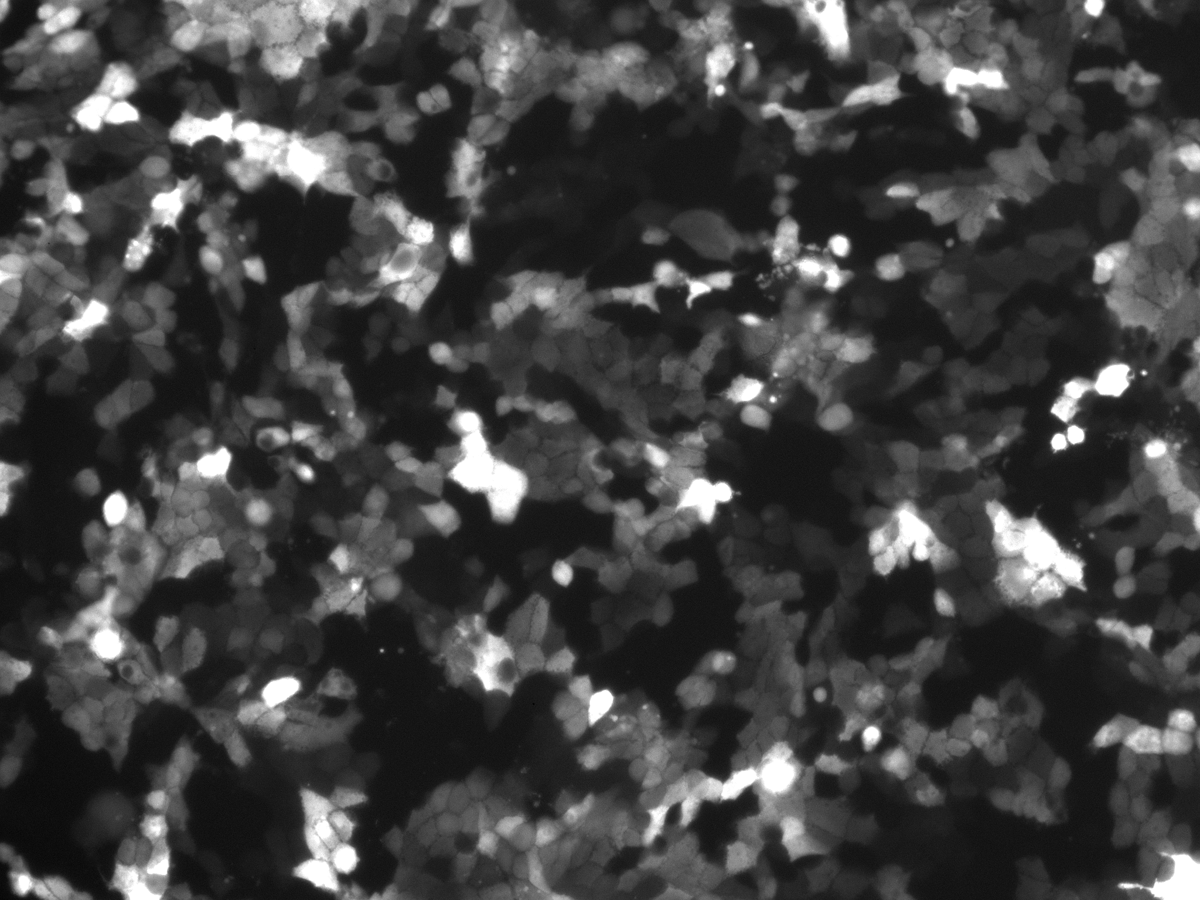

Supplement: Supplementary file 6 — Source Data [file 41467_2022_30668_MOESM6_ESM.zip › uncropped images/resized/fig.4b p2a-wt-dox gfp.tif]

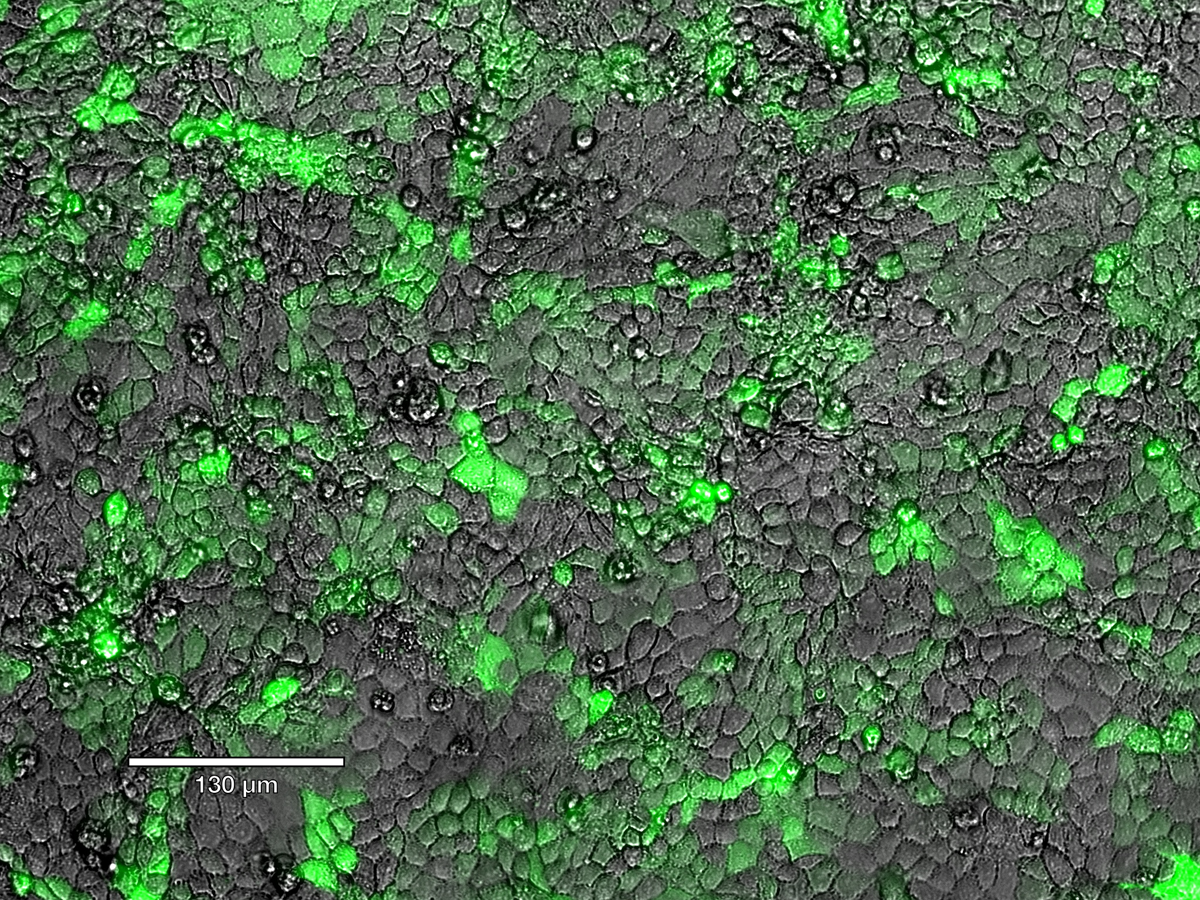

Supplement: Supplementary file 6 — Source Data [file 41467_2022_30668_MOESM6_ESM.zip › uncropped images/resized/fig.4b p2a-wt-dox merge.tif]

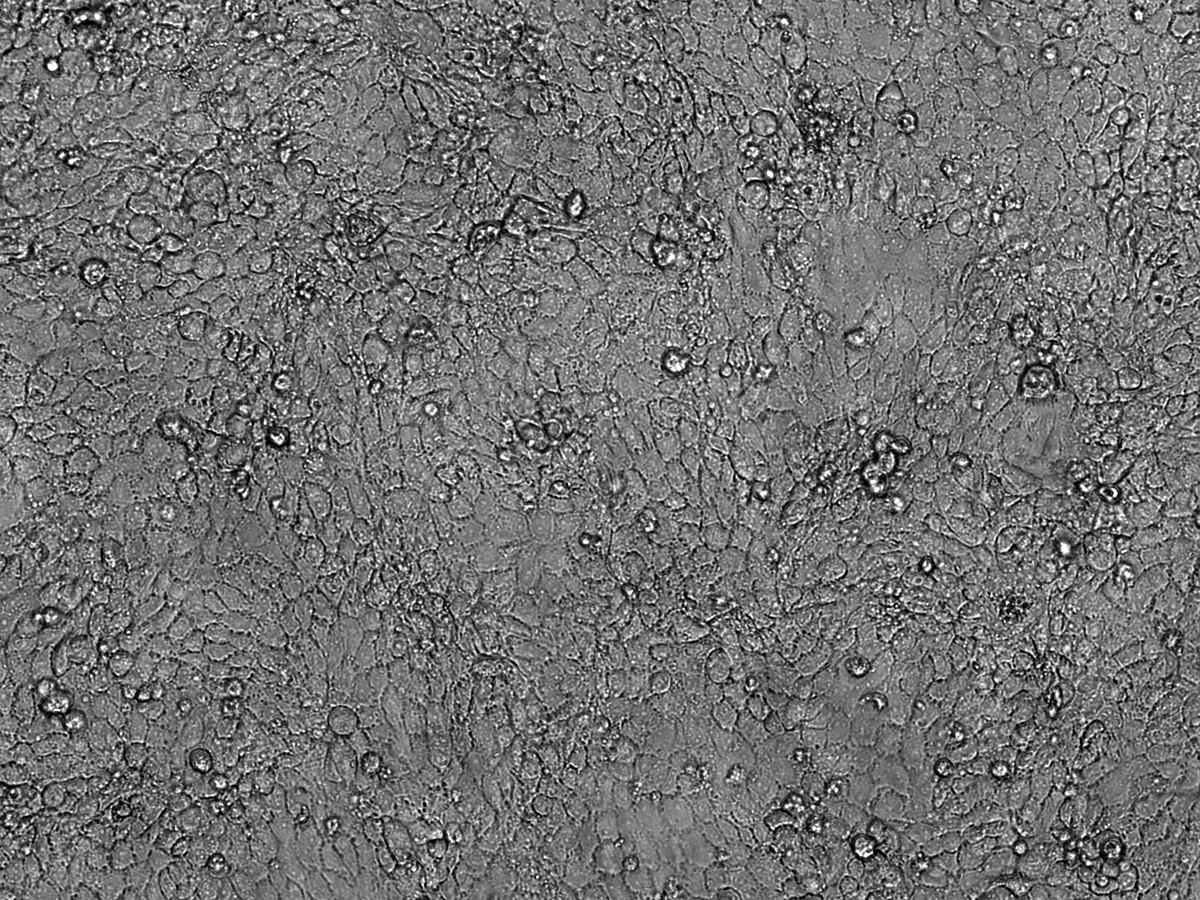

Supplement: Supplementary file 6 — Source Data [file 41467_2022_30668_MOESM6_ESM.zip › uncropped images/resized/fig.4b p2a-wt-nodox brightfield.tif]

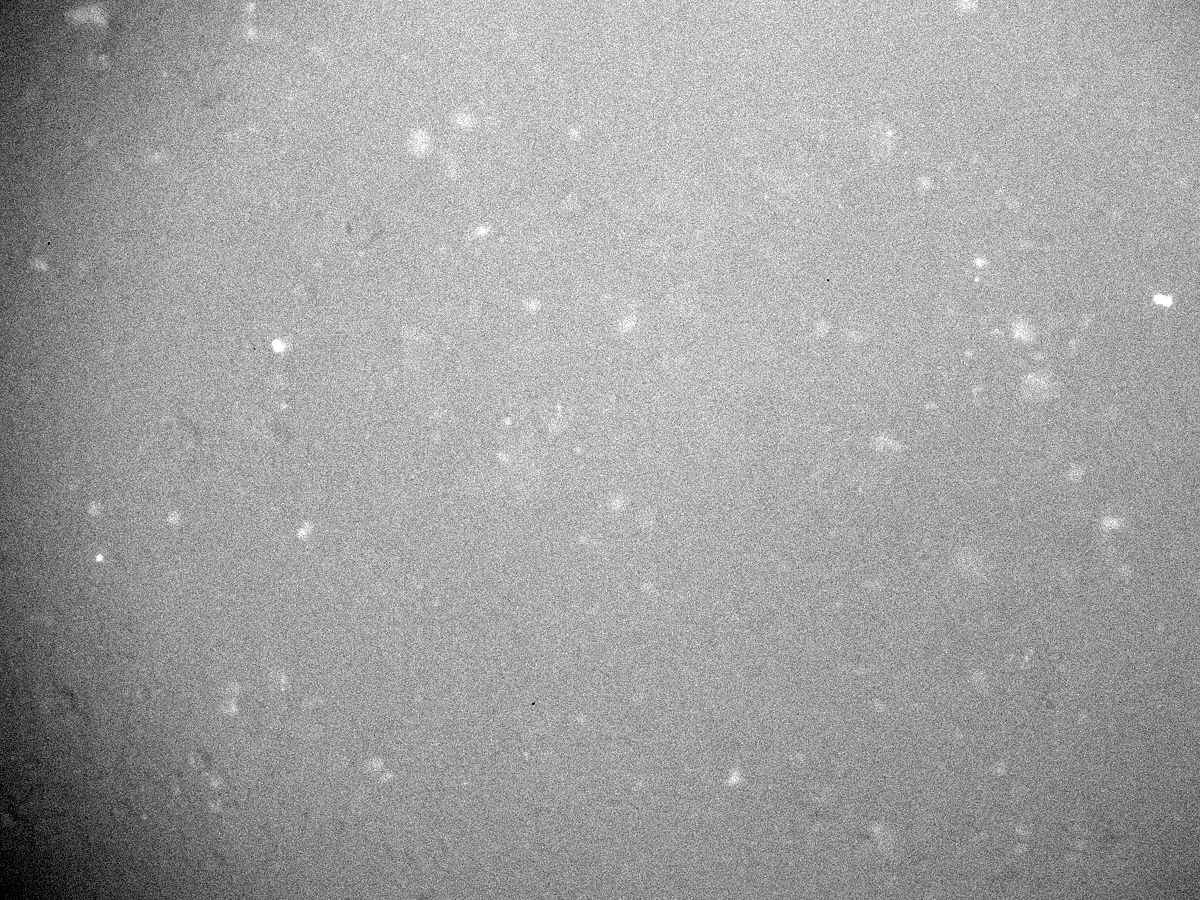

Supplement: Supplementary file 6 — Source Data [file 41467_2022_30668_MOESM6_ESM.zip › uncropped images/resized/fig.4b p2a-wt-nodox gfp.tif]

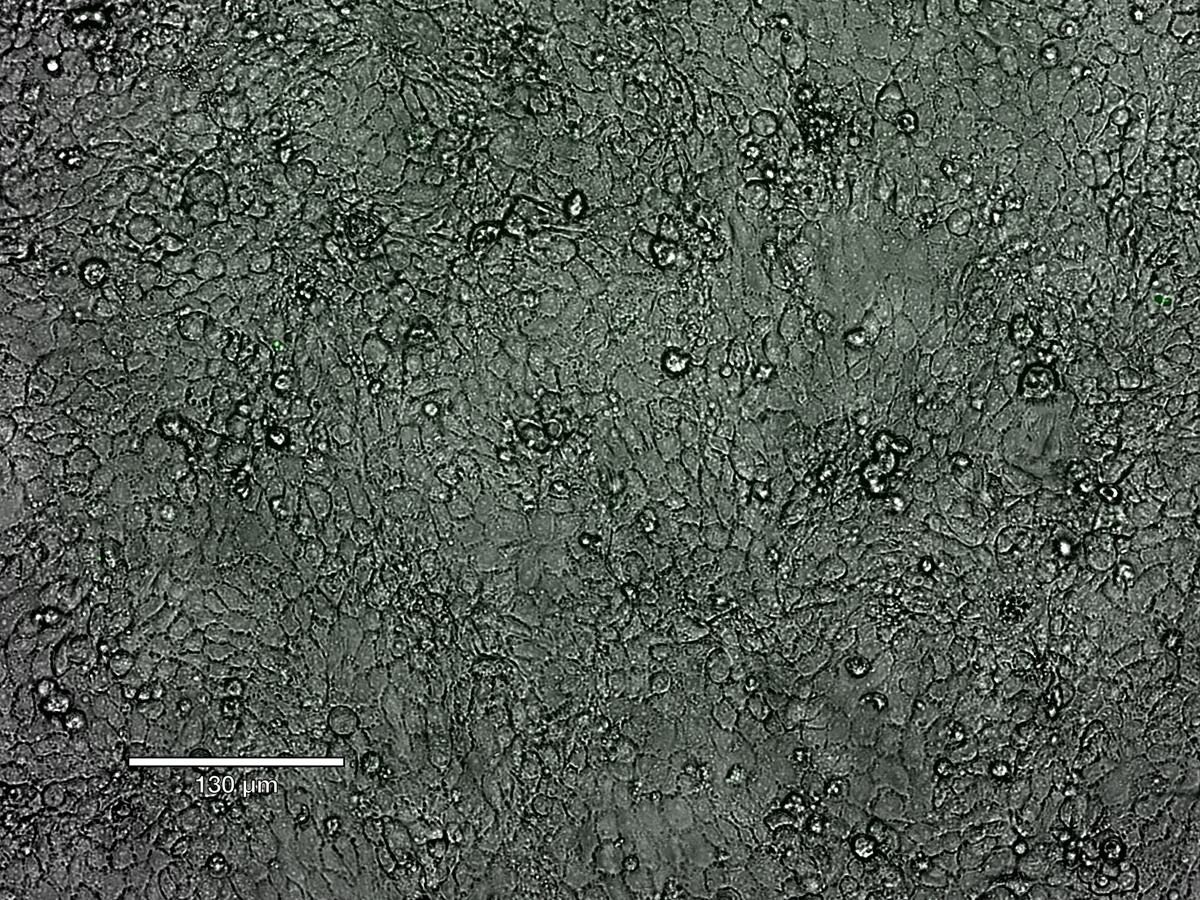

Supplement: Supplementary file 6 — Source Data [file 41467_2022_30668_MOESM6_ESM.zip › uncropped images/resized/fig.4b p2a-wt-nodox merge.tif]

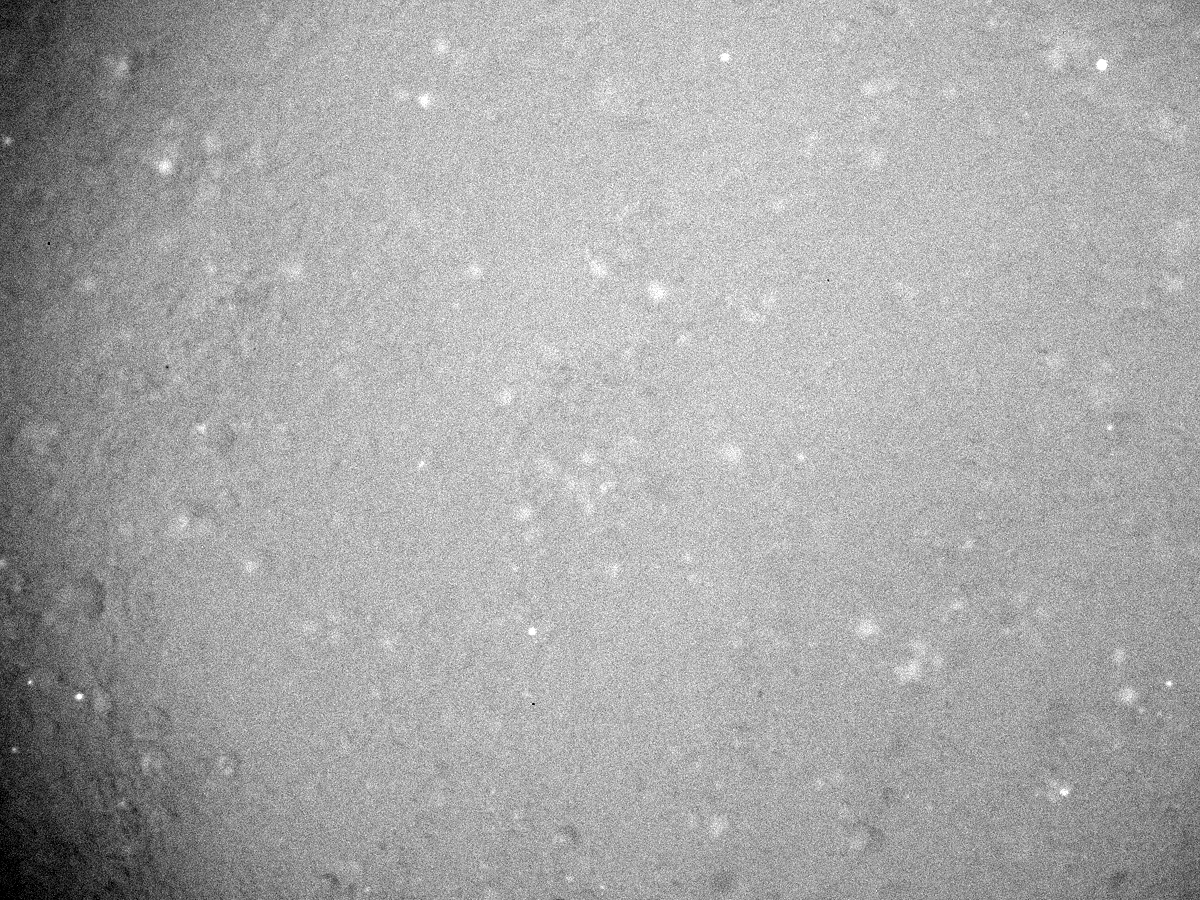

Supplement: Supplementary file 6 — Source Data [file 41467_2022_30668_MOESM6_ESM.zip › uncropped images/resized/fig.4b parental GFP.tif]

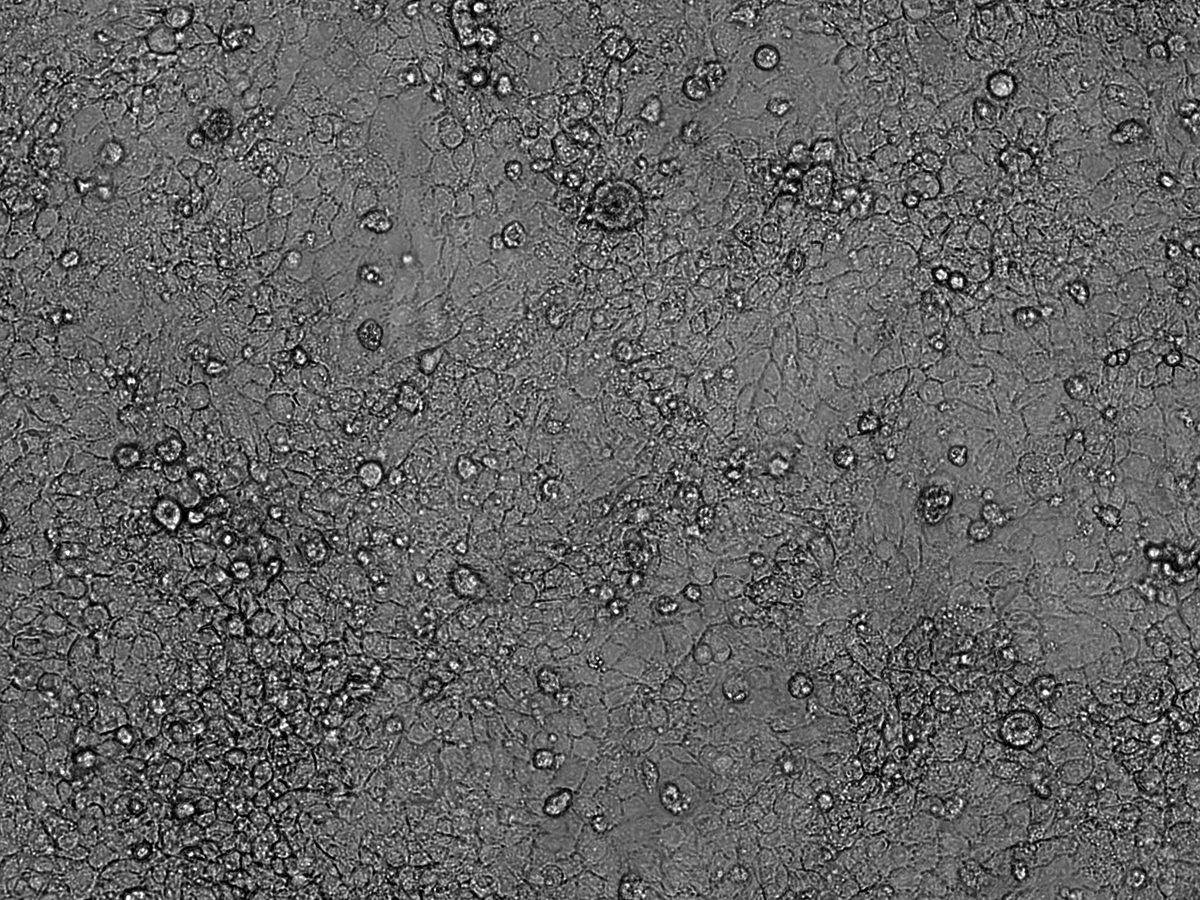

Supplement: Supplementary file 6 — Source Data [file 41467_2022_30668_MOESM6_ESM.zip › uncropped images/resized/fig.4b parental bright field.tif]

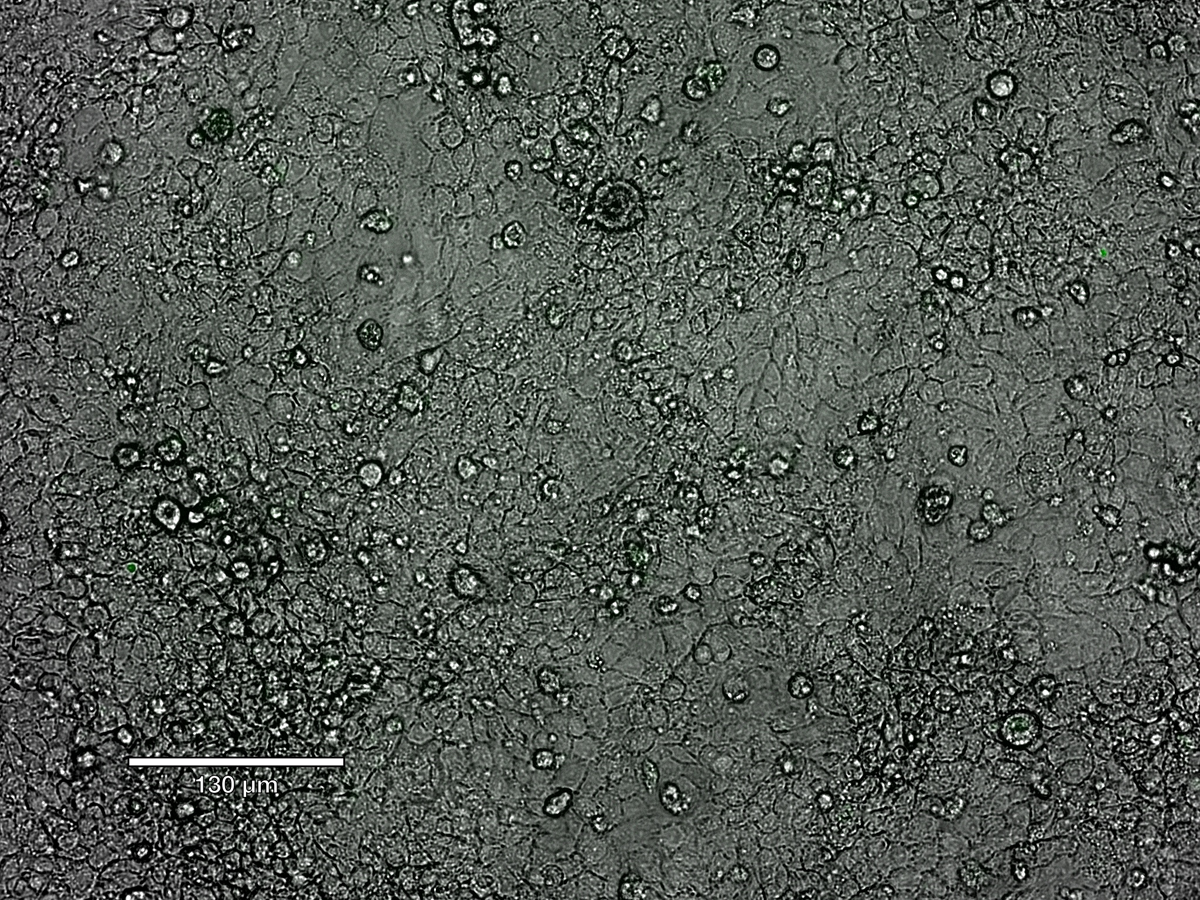

Supplement: Supplementary file 6 — Source Data [file 41467_2022_30668_MOESM6_ESM.zip › uncropped images/resized/fig.4b parental merge.tif]

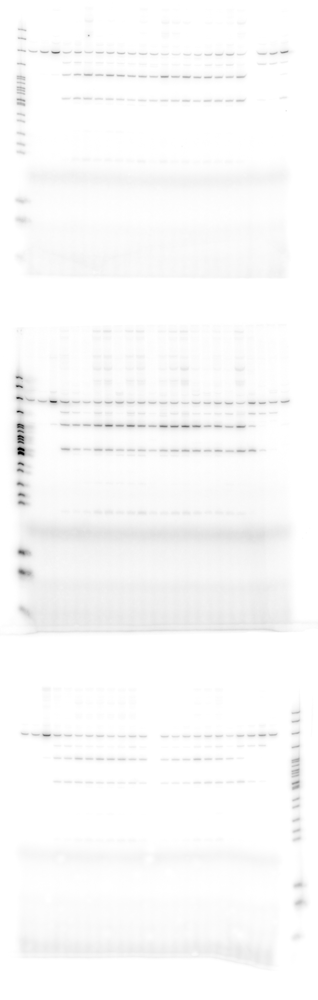

Supplement: Supplementary file 6 — Source Data [file 41467_2022_30668_MOESM6_ESM.zip › uncropped images/resized/fig.S10a.tif]

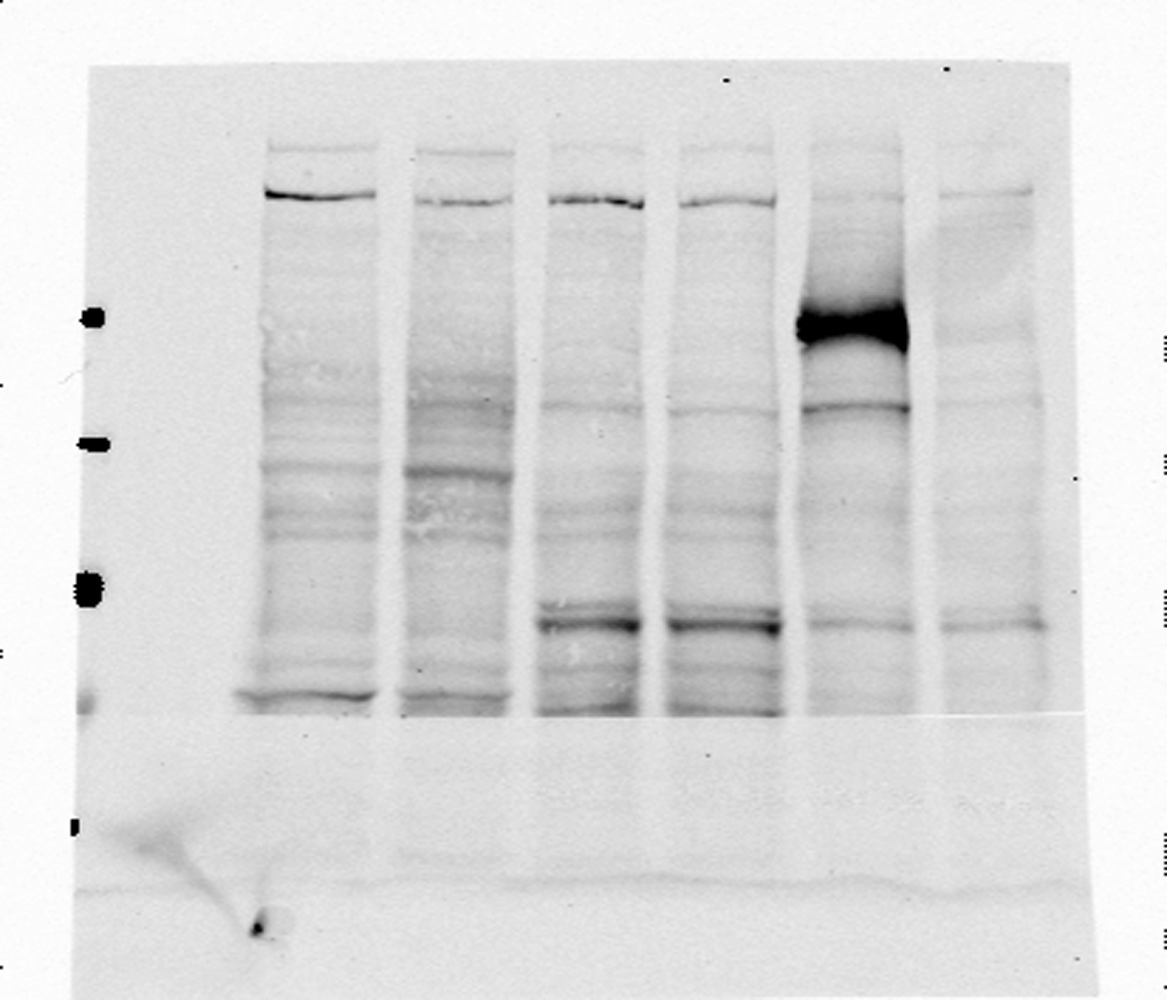

Supplement: Supplementary file 6 — Source Data [file 41467_2022_30668_MOESM6_ESM.zip › uncropped images/resized/fig.S11a-CFTR.tif]

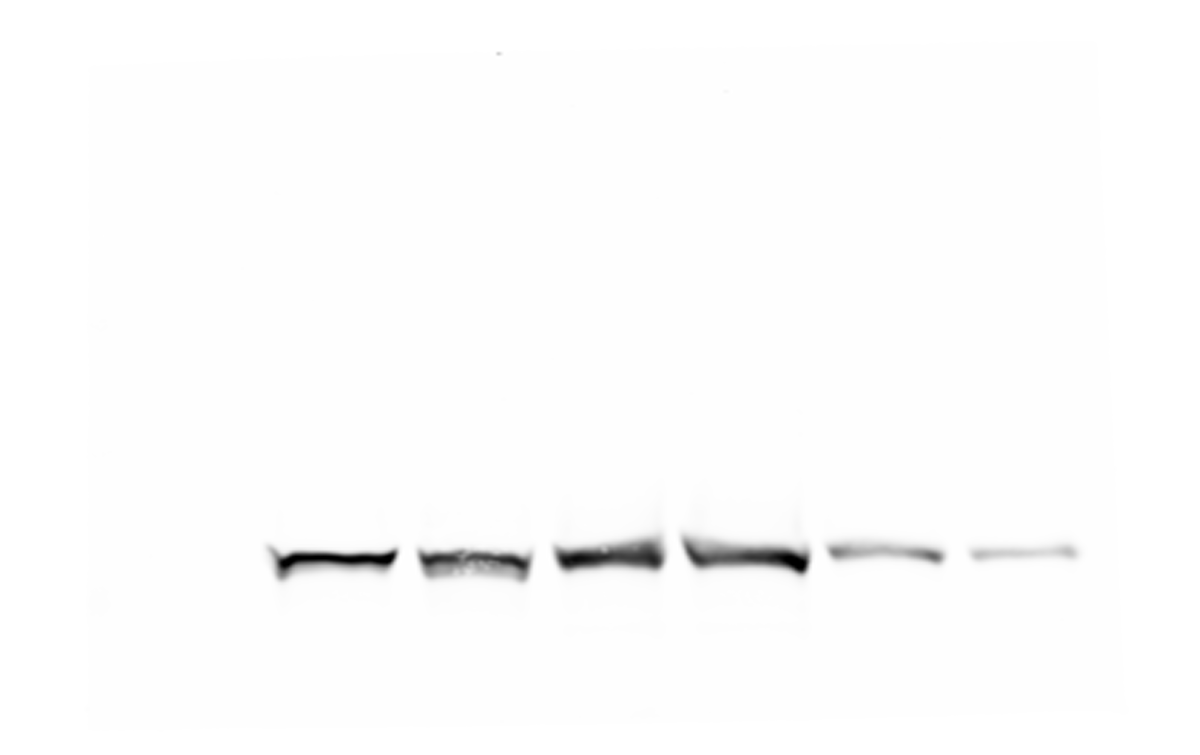

Supplement: Supplementary file 6 — Source Data [file 41467_2022_30668_MOESM6_ESM.zip › uncropped images/resized/fig.S11a-NaKATPase.tif]

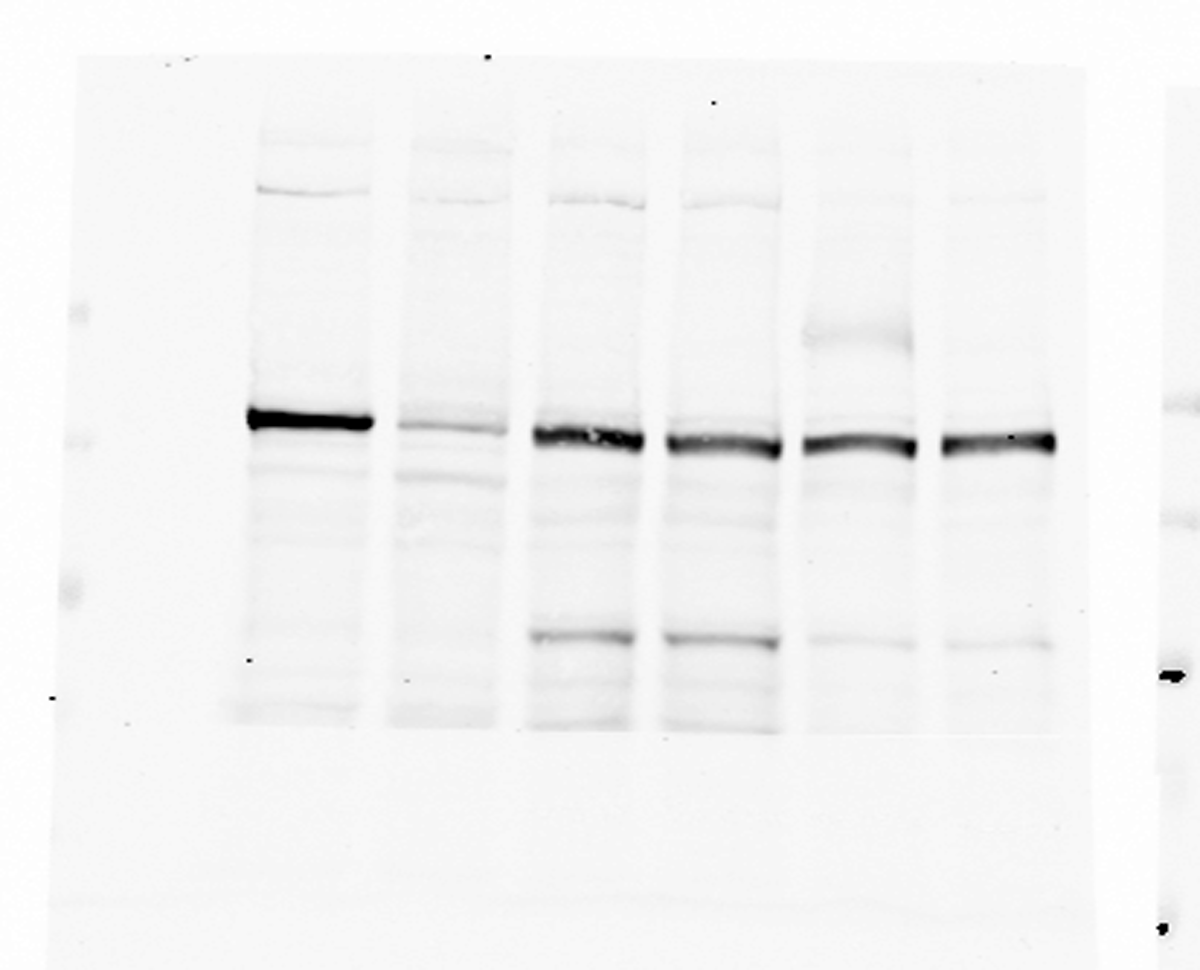

Supplement: Supplementary file 6 — Source Data [file 41467_2022_30668_MOESM6_ESM.zip › uncropped images/resized/fig.S11a-UPF1.tif]

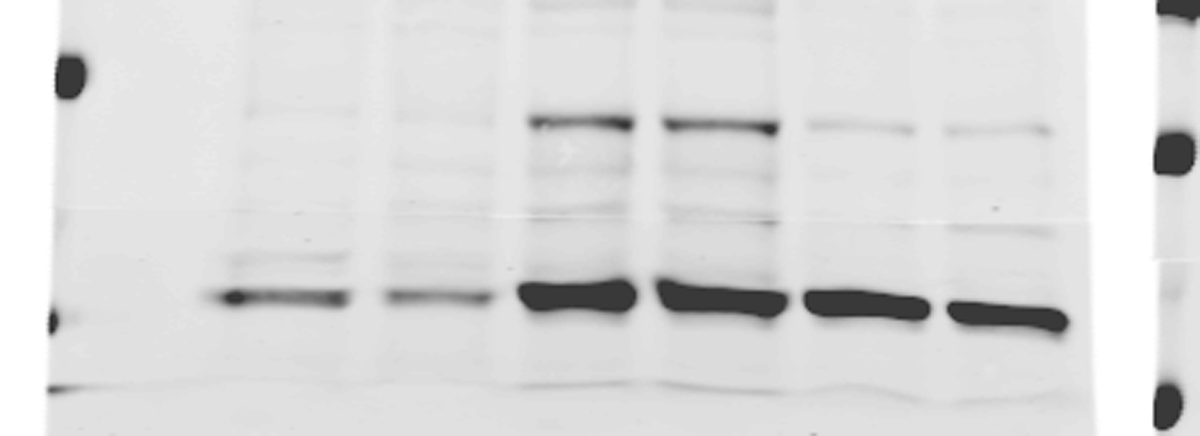

Supplement: Supplementary file 6 — Source Data [file 41467_2022_30668_MOESM6_ESM.zip › uncropped images/resized/fig.S11a-tubulin.tif]

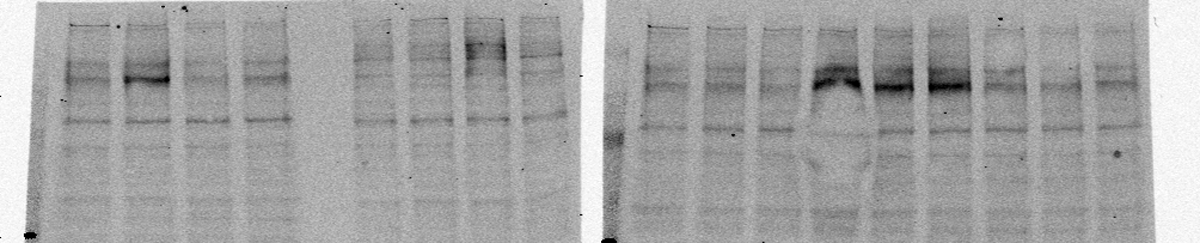

Supplement: Supplementary file 6 — Source Data [file 41467_2022_30668_MOESM6_ESM.zip › uncropped images/resized/fig.S11b-CFTR representative.tif]

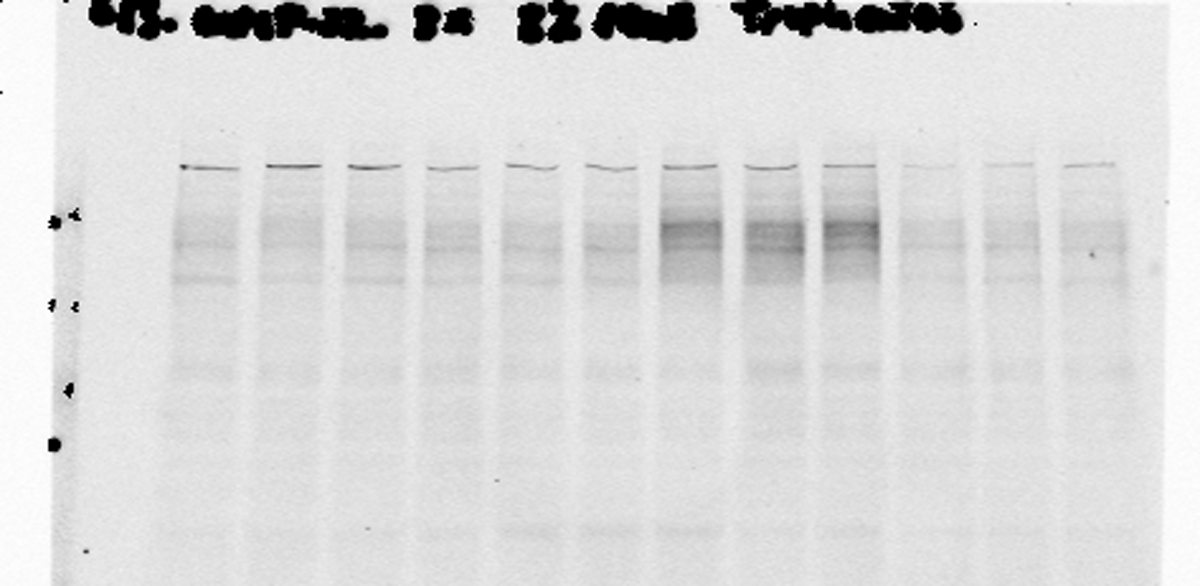

Supplement: Supplementary file 6 — Source Data [file 41467_2022_30668_MOESM6_ESM.zip › uncropped images/resized/fig.S11b-CFTR.tif]

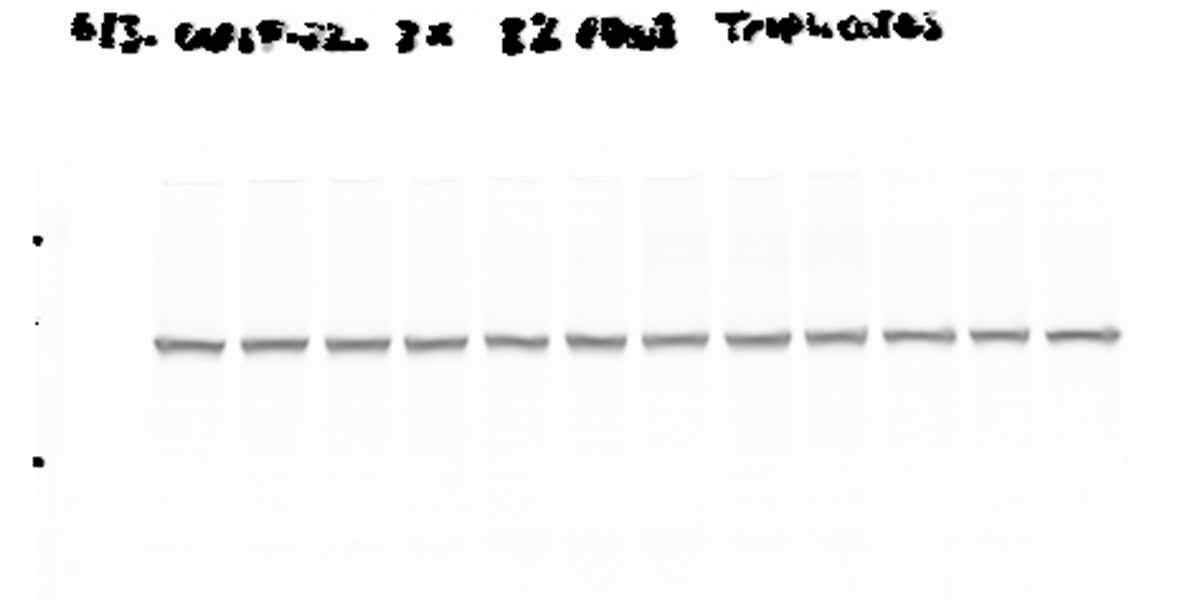

Supplement: Supplementary file 6 — Source Data [file 41467_2022_30668_MOESM6_ESM.zip › uncropped images/resized/fig.S11b-NaKATPase (2).tif]

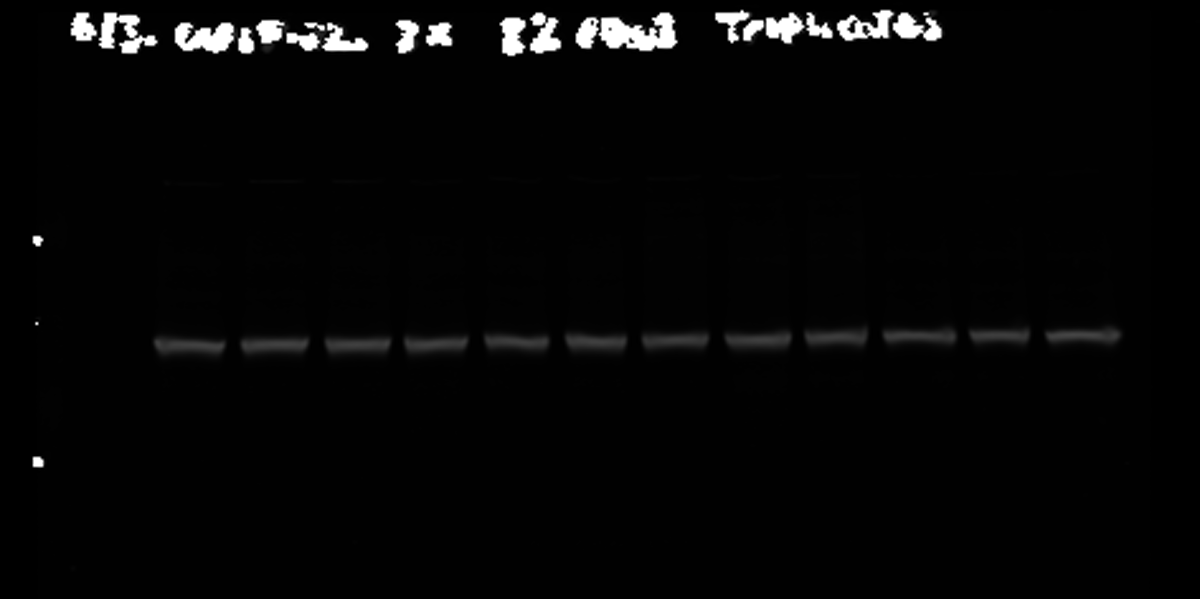

Supplement: Supplementary file 6 — Source Data [file 41467_2022_30668_MOESM6_ESM.zip › uncropped images/resized/fig.S11b-NaKATPase.tif]

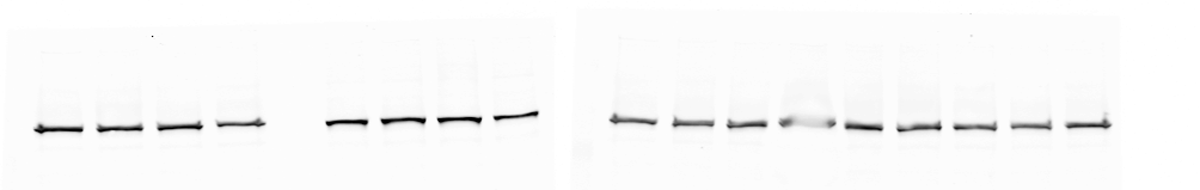

Supplement: Supplementary file 6 — Source Data [file 41467_2022_30668_MOESM6_ESM.zip › uncropped images/resized/fig.S11b-representative NaKATPase.tif]

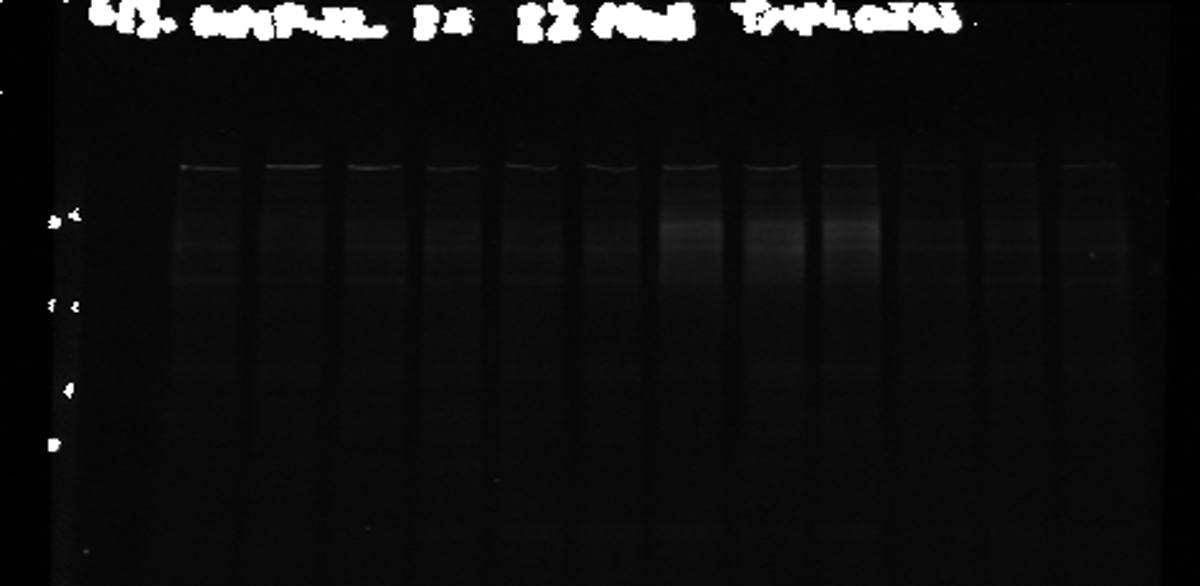

Supplement: Supplementary file 6 — Source Data [file 41467_2022_30668_MOESM6_ESM.zip › uncropped images/resized/fig.S11b.tif]

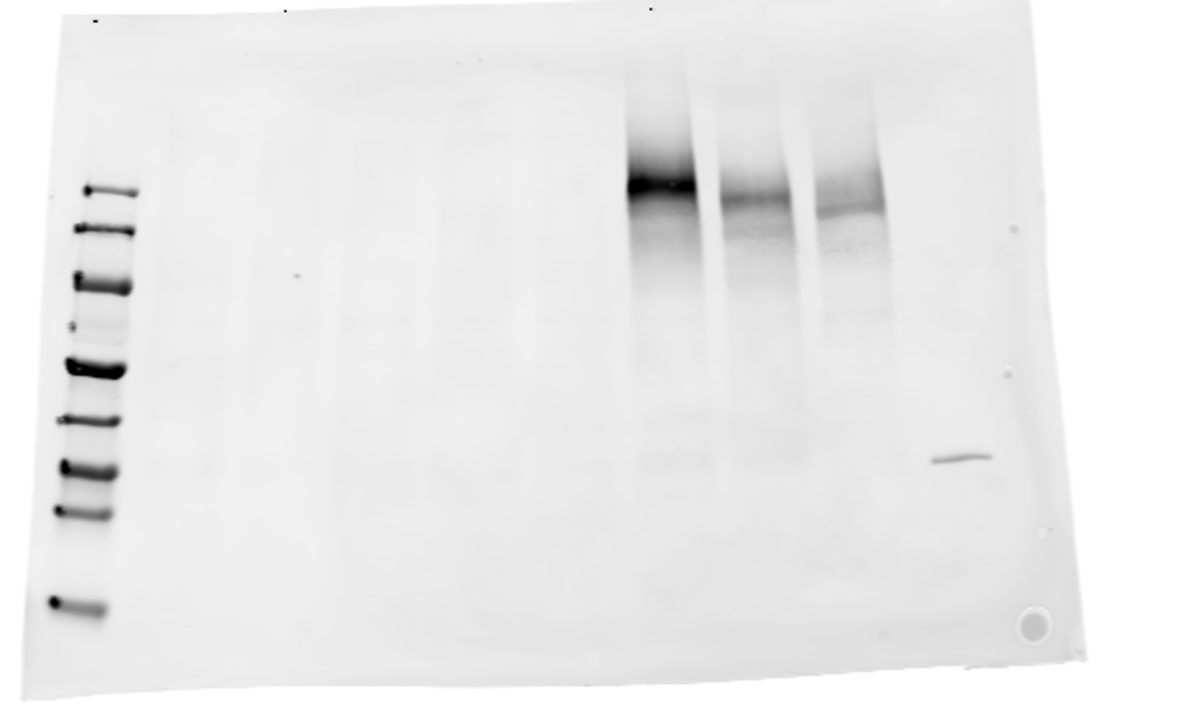

Supplement: Supplementary file 6 — Source Data [file 41467_2022_30668_MOESM6_ESM.zip › uncropped images/resized/fig.S12-CFTR.tif]

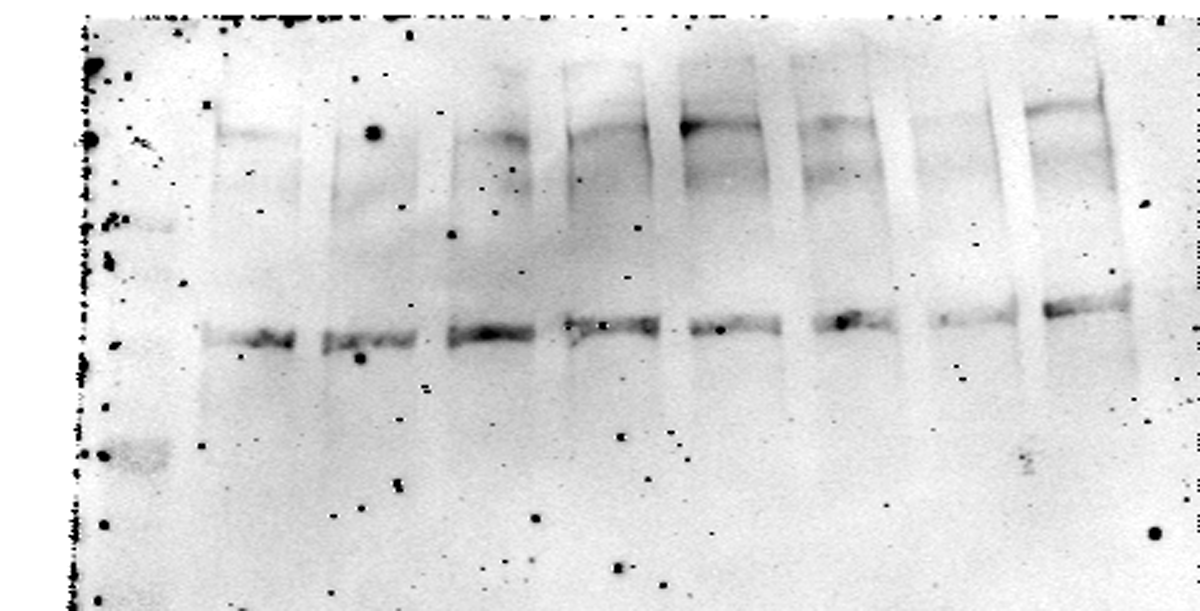

Supplement: Supplementary file 6 — Source Data [file 41467_2022_30668_MOESM6_ESM.zip › uncropped images/resized/fig.S12-NaKATPase.tif]

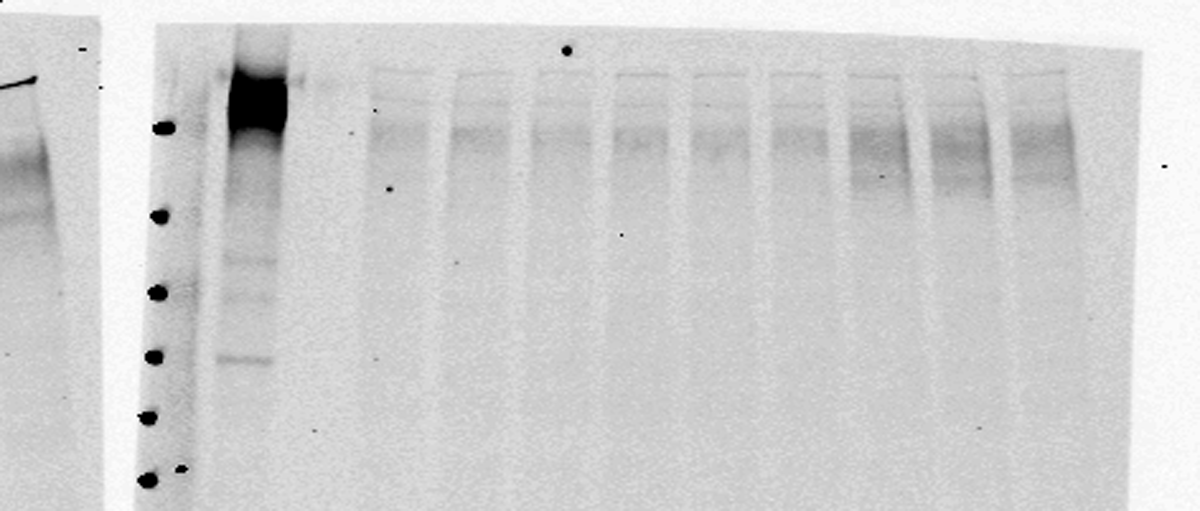

Supplement: Supplementary file 6 — Source Data [file 41467_2022_30668_MOESM6_ESM.zip › uncropped images/resized/fig.S14a-CFTR.tif]

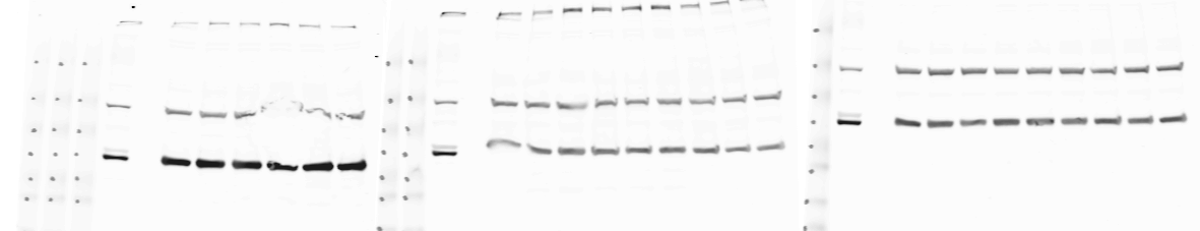

Supplement: Supplementary file 6 — Source Data [file 41467_2022_30668_MOESM6_ESM.zip › uncropped images/resized/fig.S14a-NaKATPase and Tubulin.tif]

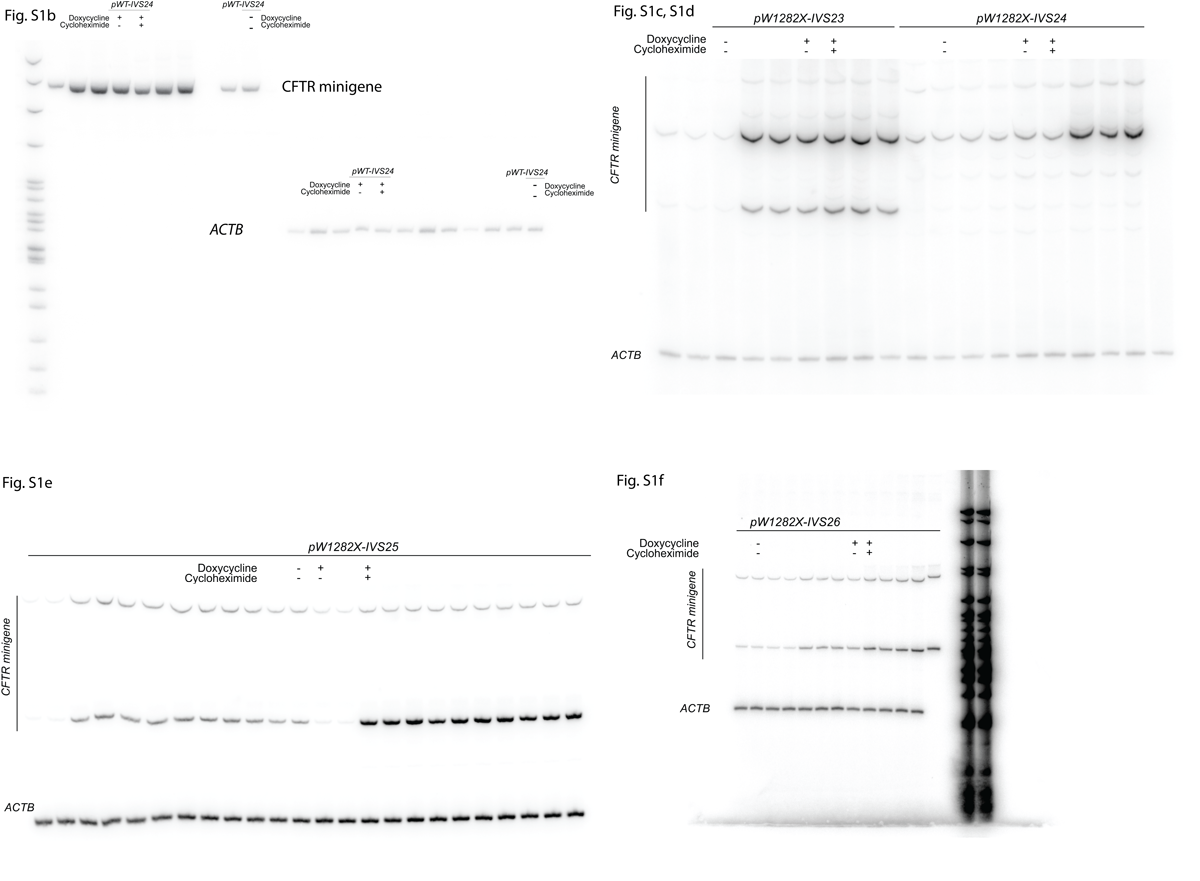

Supplement: Supplementary file 6 — Source Data [file 41467_2022_30668_MOESM6_ESM.zip › uncropped images/resized/fig.S1b-f summary.tif]

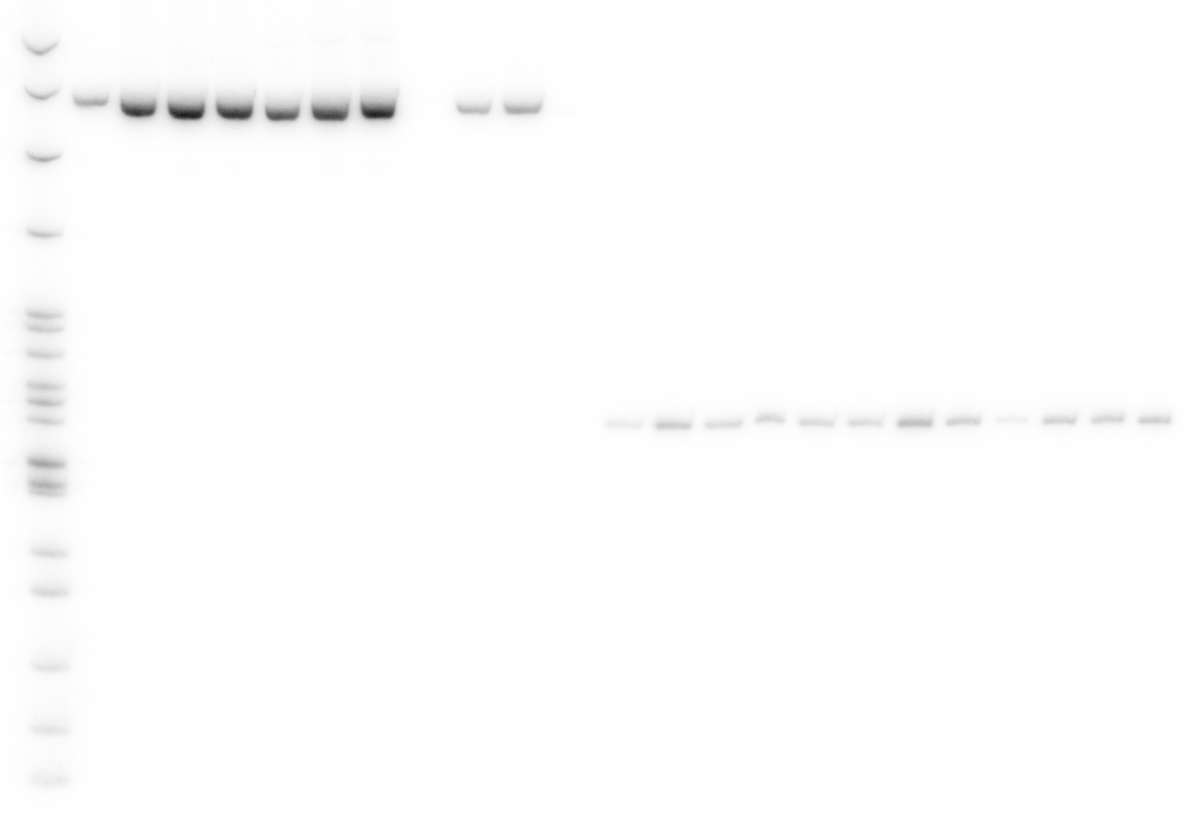

Supplement: Supplementary file 6 — Source Data [file 41467_2022_30668_MOESM6_ESM.zip › uncropped images/resized/fig.S1b.tif]

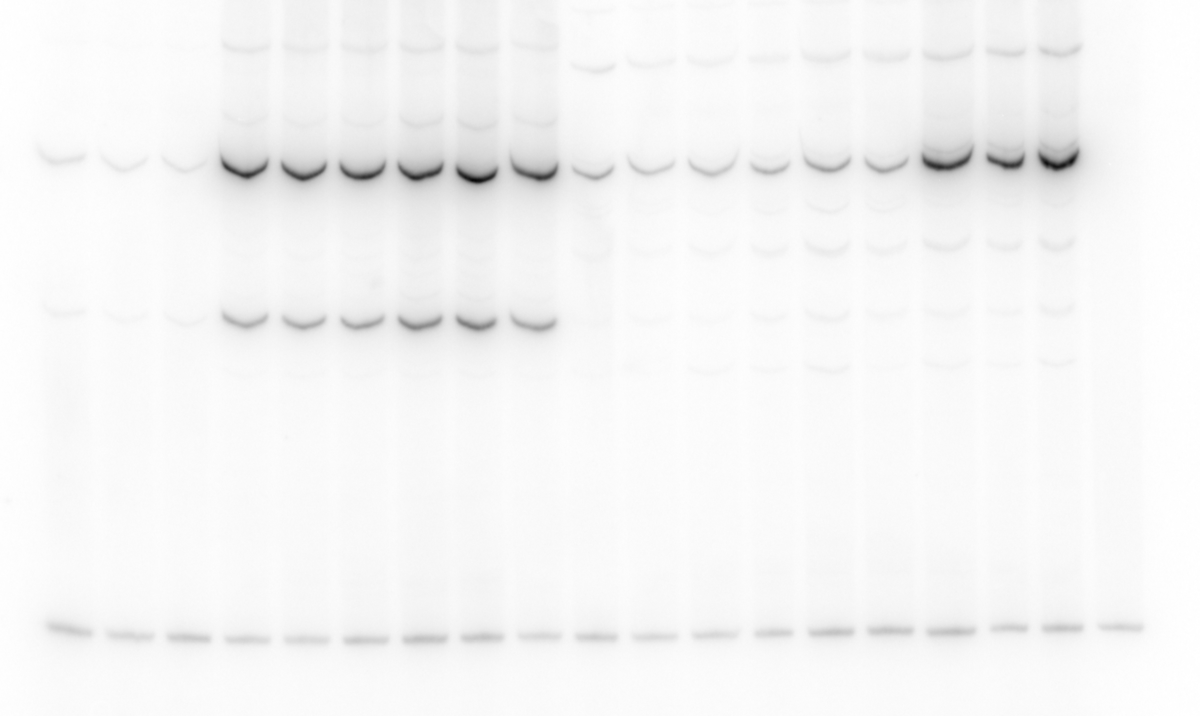

Supplement: Supplementary file 6 — Source Data [file 41467_2022_30668_MOESM6_ESM.zip › uncropped images/resized/fig.S1c-d.tif]

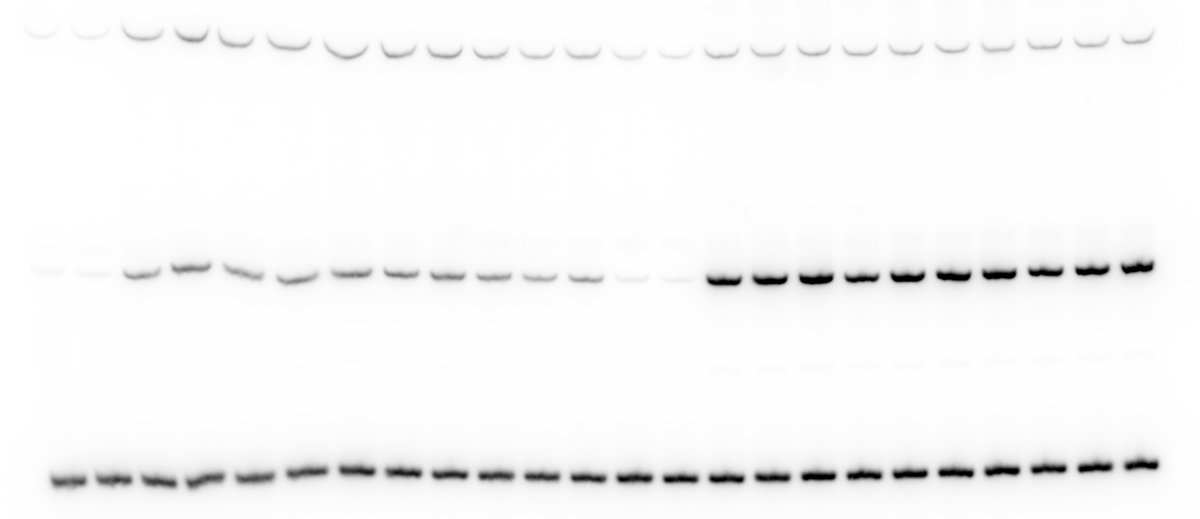

Supplement: Supplementary file 6 — Source Data [file 41467_2022_30668_MOESM6_ESM.zip › uncropped images/resized/fig.S1e.tif]

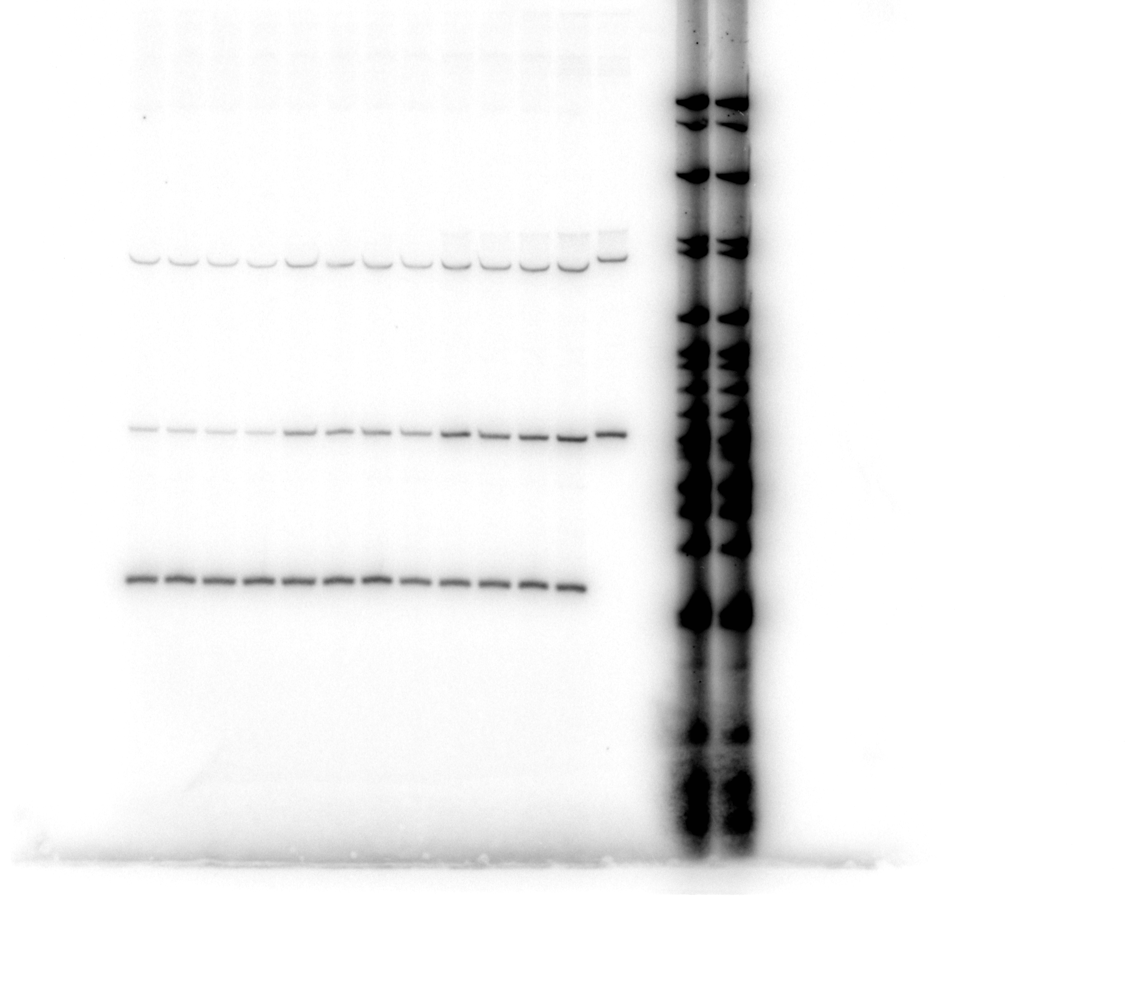

Supplement: Supplementary file 6 — Source Data [file 41467_2022_30668_MOESM6_ESM.zip › uncropped images/resized/fig.S1f.tif]

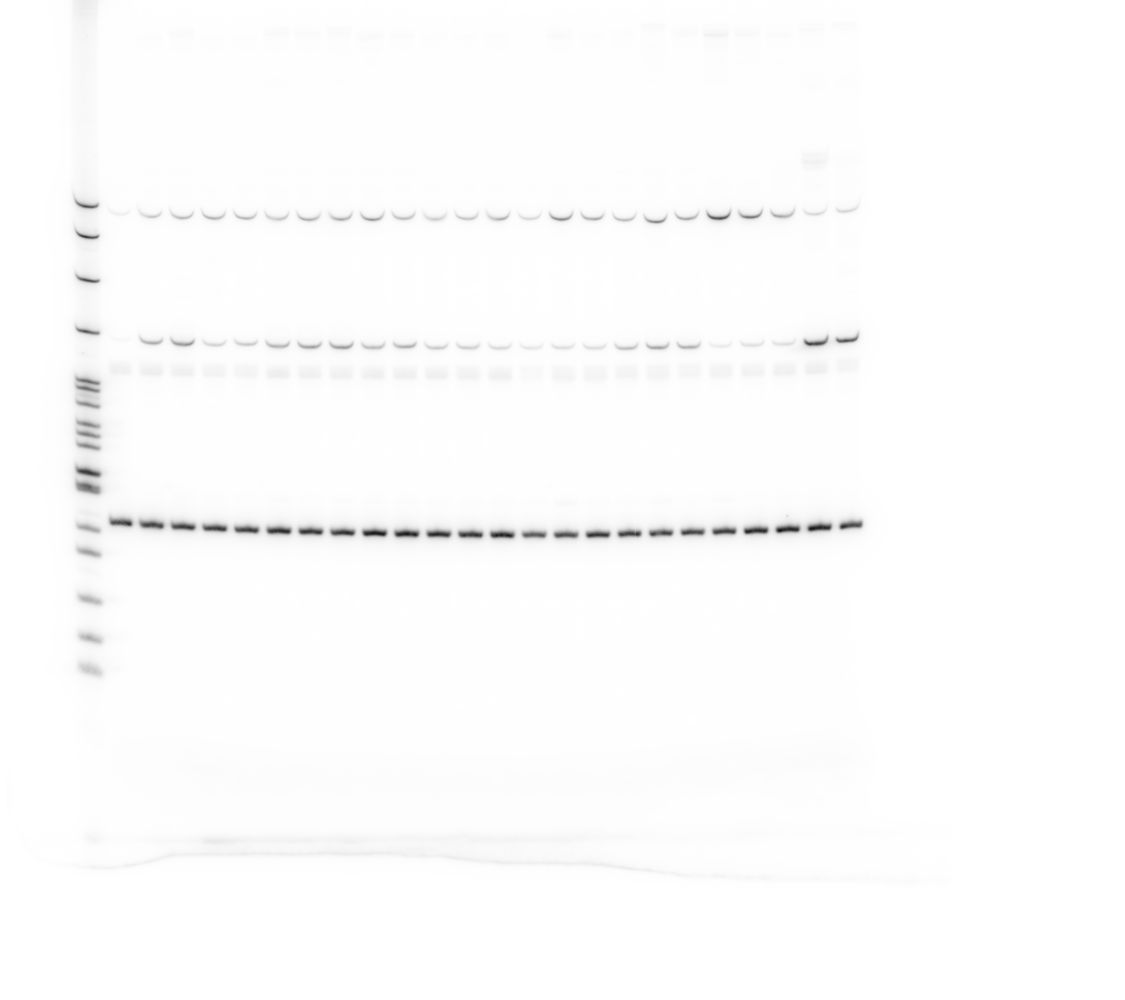

Supplement: Supplementary file 6 — Source Data [file 41467_2022_30668_MOESM6_ESM.zip › uncropped images/resized/fig.S2a.tif]

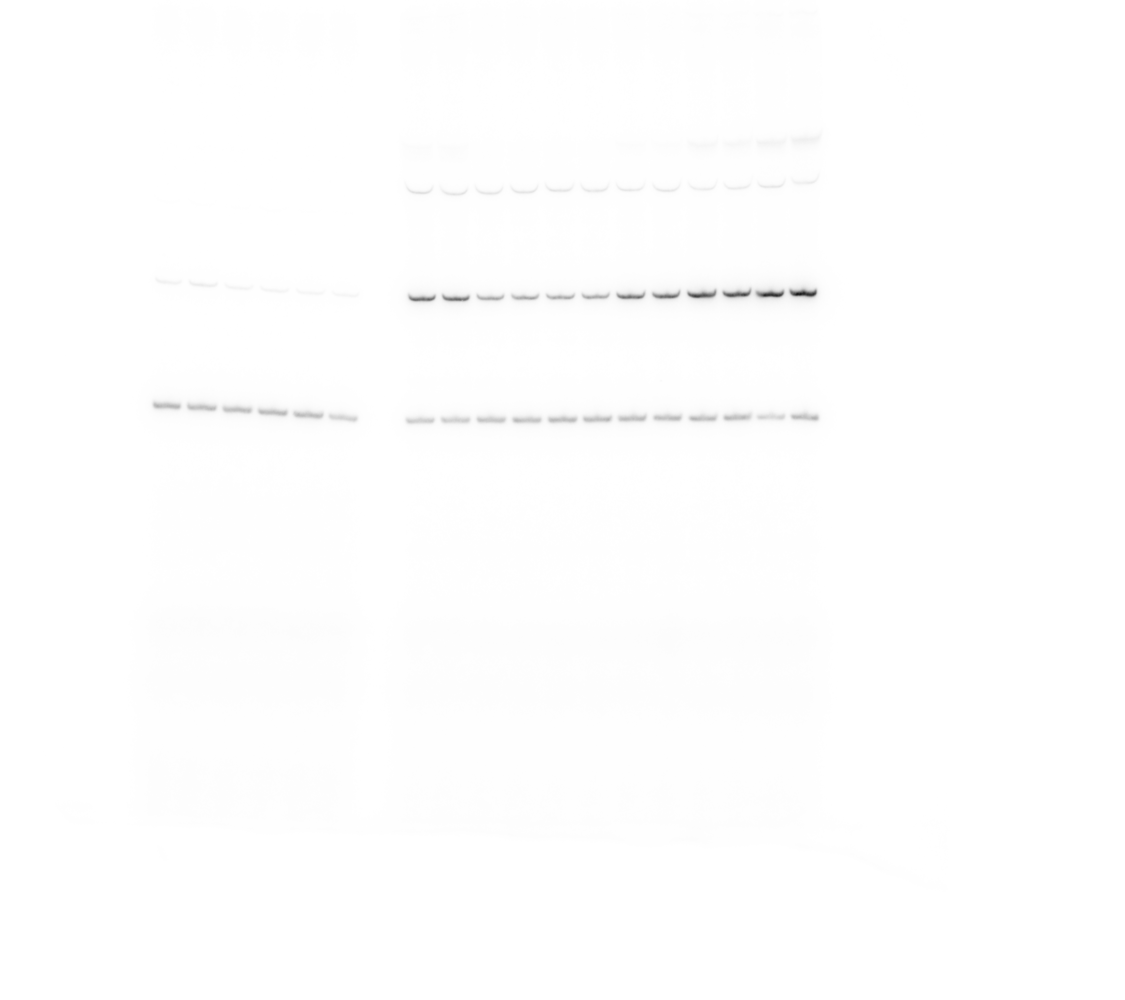

Supplement: Supplementary file 6 — Source Data [file 41467_2022_30668_MOESM6_ESM.zip › uncropped images/resized/fig.S2c.tif]

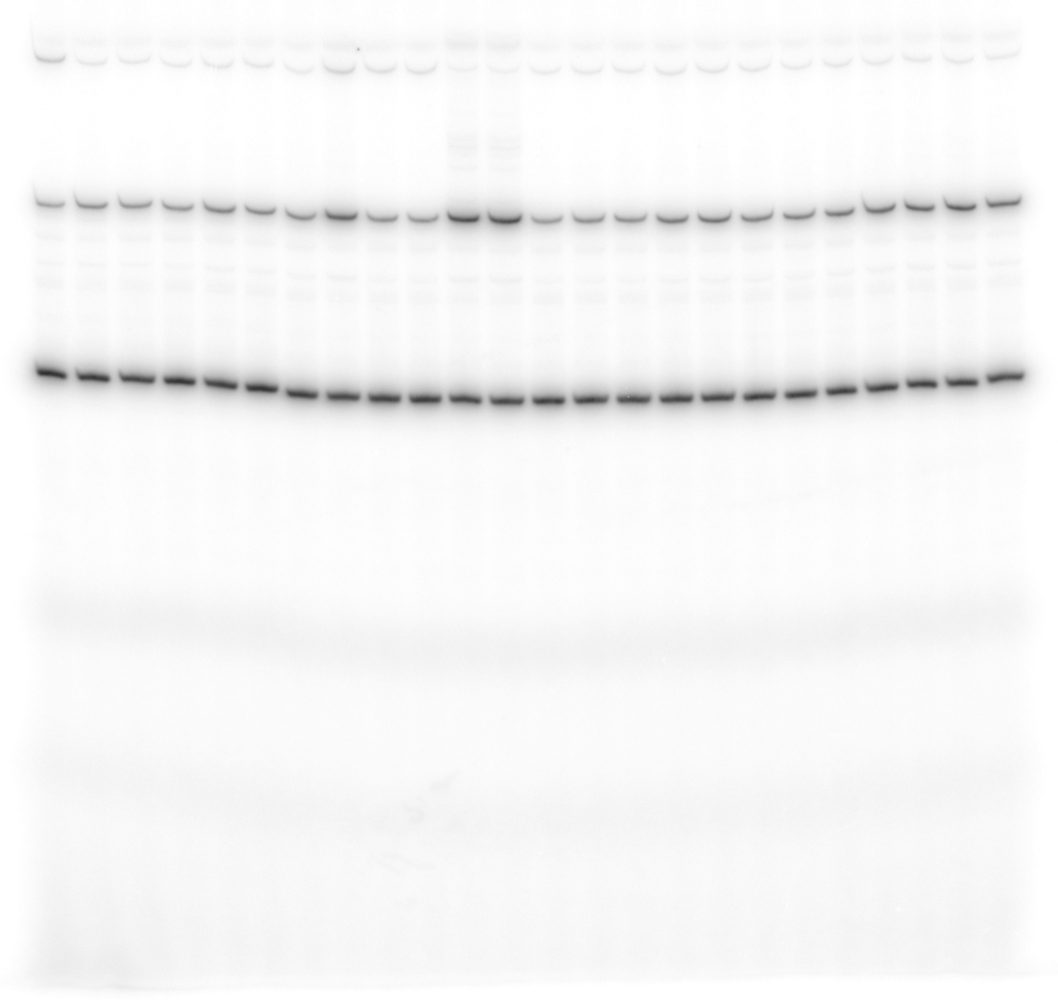

Supplement: Supplementary file 6 — Source Data [file 41467_2022_30668_MOESM6_ESM.zip › uncropped images/resized/fig.S2d.tif]

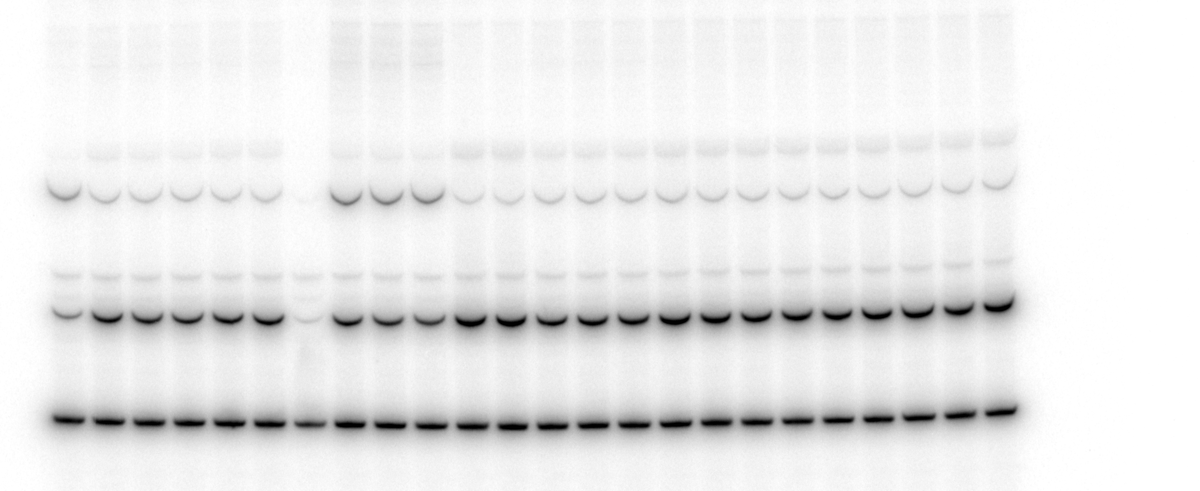

Supplement: Supplementary file 6 — Source Data [file 41467_2022_30668_MOESM6_ESM.zip › uncropped images/resized/fig.S2e.tif]

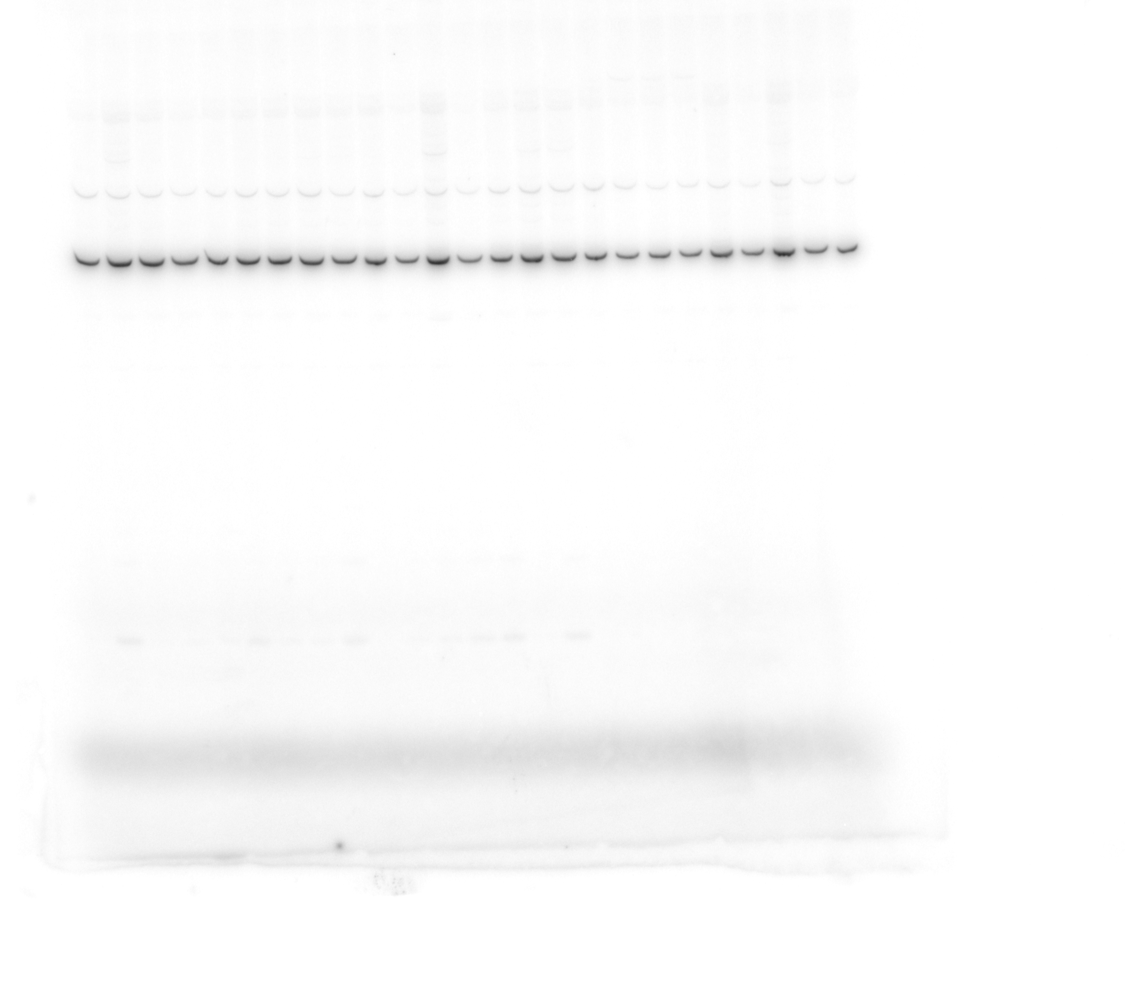

Supplement: Supplementary file 6 — Source Data [file 41467_2022_30668_MOESM6_ESM.zip › uncropped images/resized/fig.S7a bottom panel.tif]

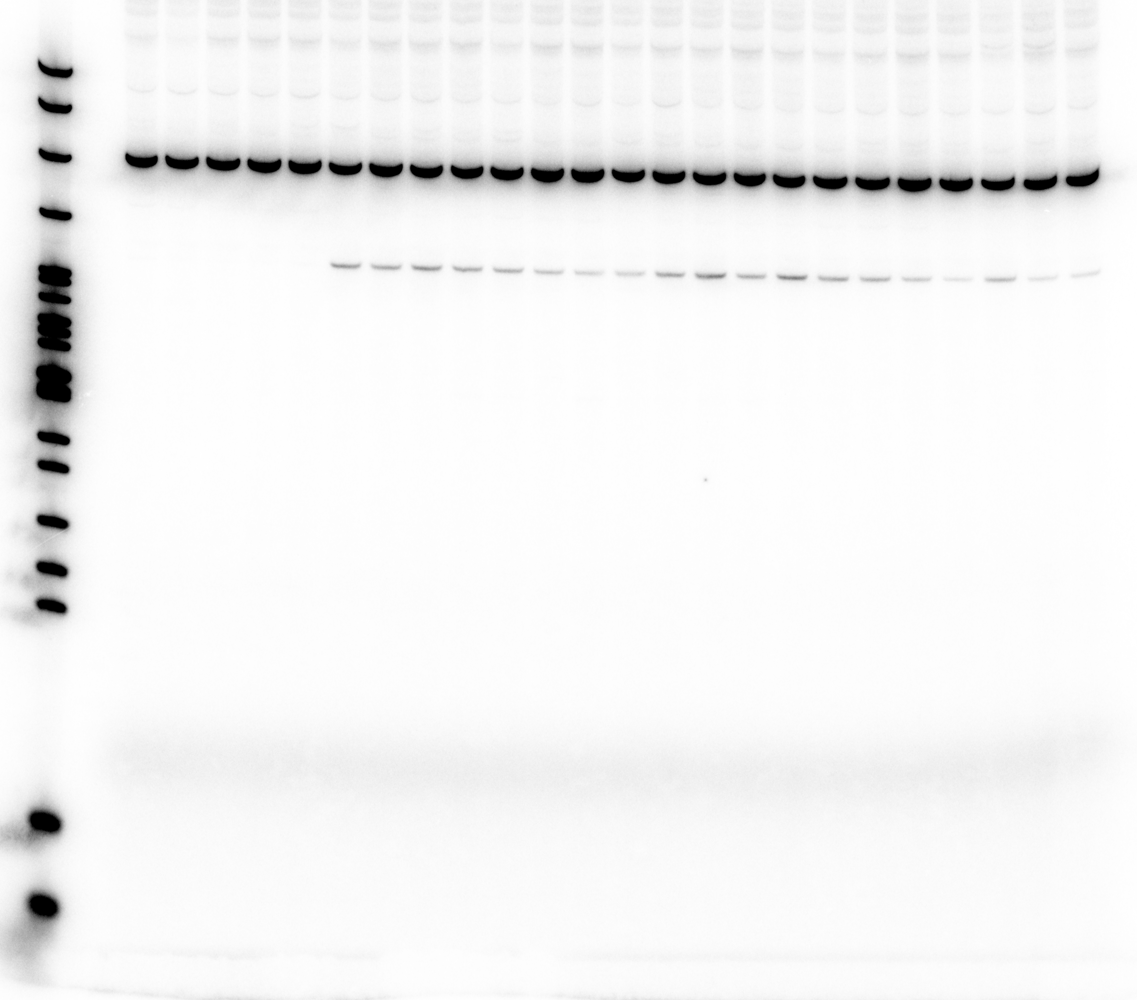

Supplement: Supplementary file 6 — Source Data [file 41467_2022_30668_MOESM6_ESM.zip › uncropped images/resized/fig.S7a middle panel.tif]

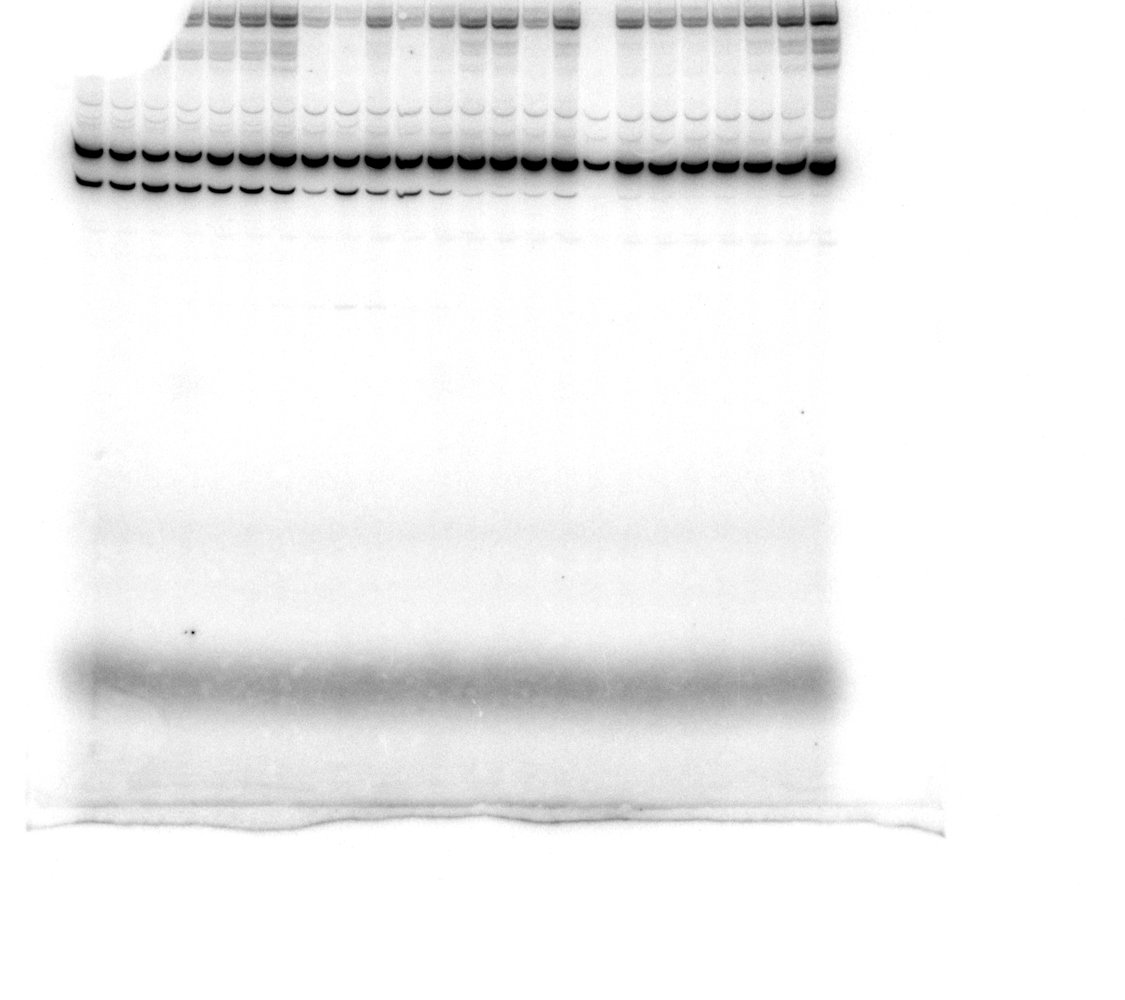

Supplement: Supplementary file 6 — Source Data [file 41467_2022_30668_MOESM6_ESM.zip › uncropped images/resized/fig.S7a top panel.tif]

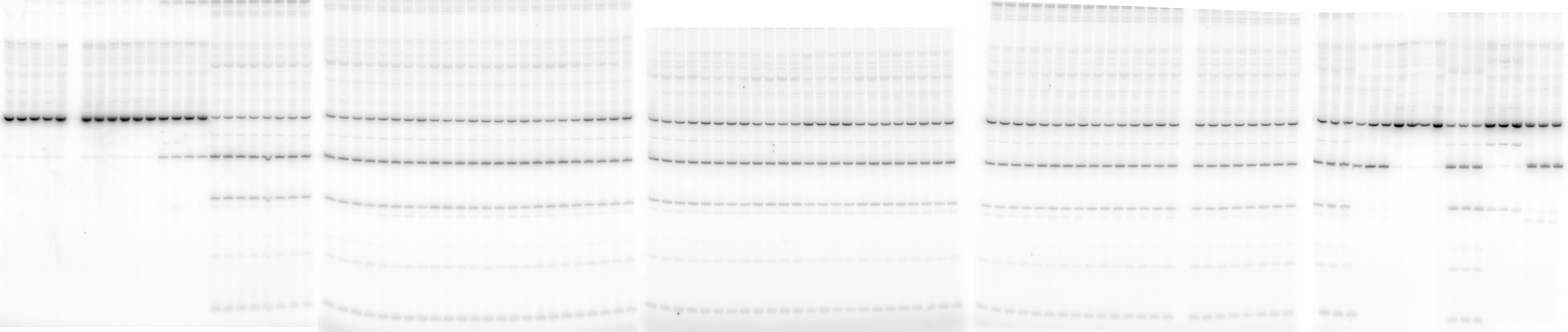

Supplement: Supplementary file 6 — Source Data [file 41467_2022_30668_MOESM6_ESM.zip › uncropped images/resized/fig.S8a.tif]

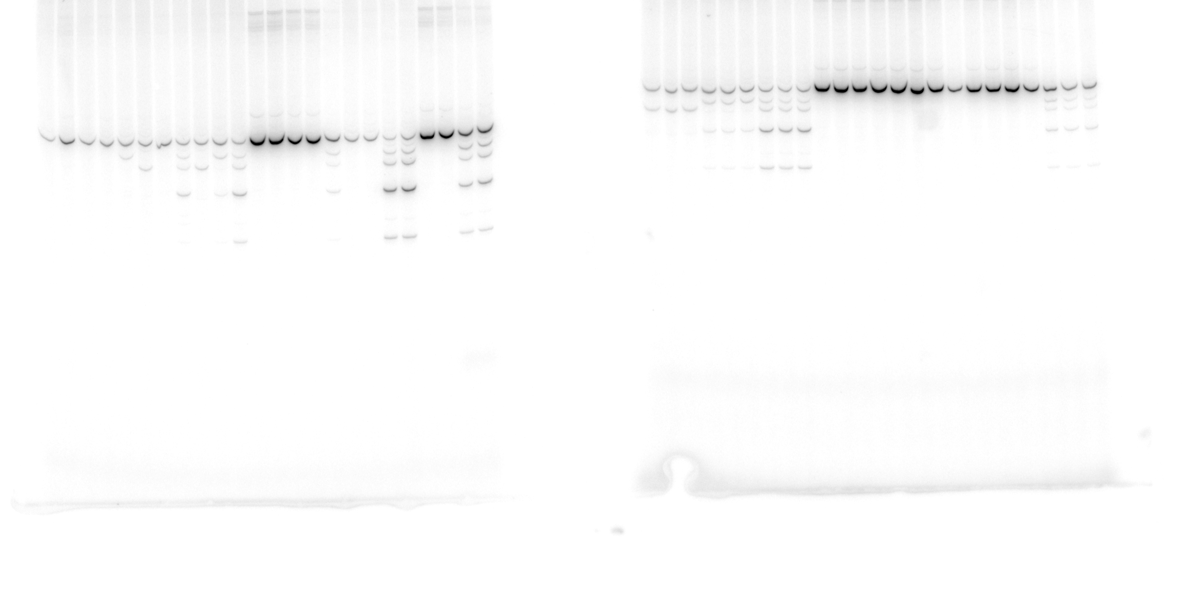

Supplement: Supplementary file 6 — Source Data [file 41467_2022_30668_MOESM6_ESM.zip › uncropped images/resized/fig.S8b,S8e.tif]

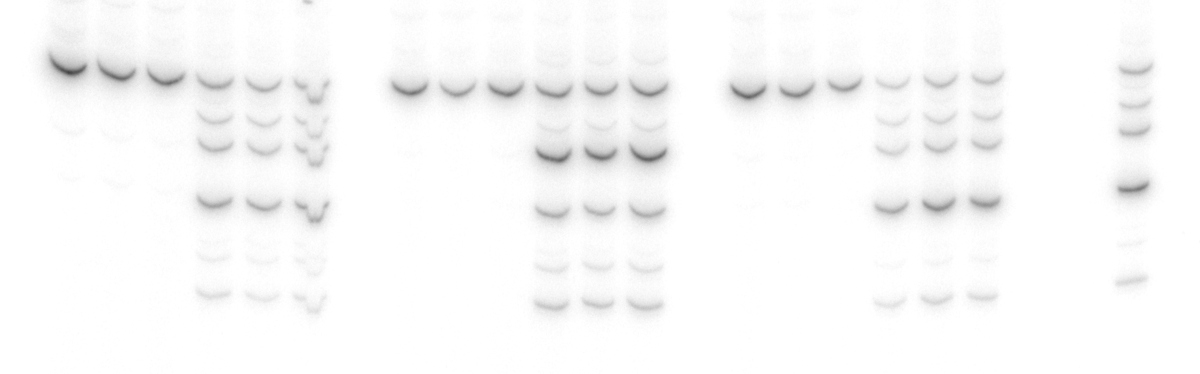

Supplement: Supplementary file 6 — Source Data [file 41467_2022_30668_MOESM6_ESM.zip › uncropped images/resized/fig.S9a.tif]

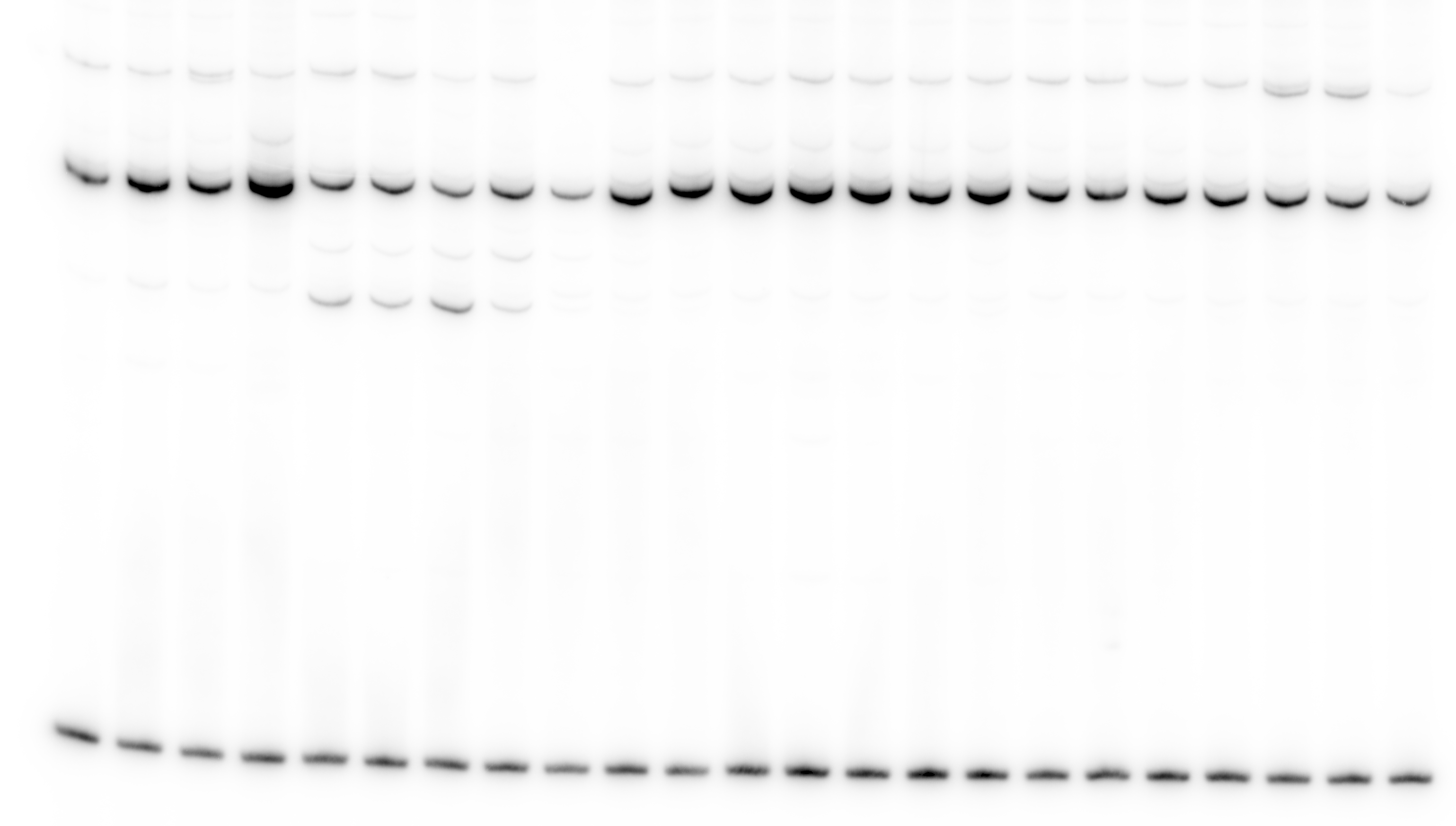

Supplement: Supplementary file 6 — Source Data [file 41467_2022_30668_MOESM6_ESM.zip › uncropped images/fig.1c.tif]

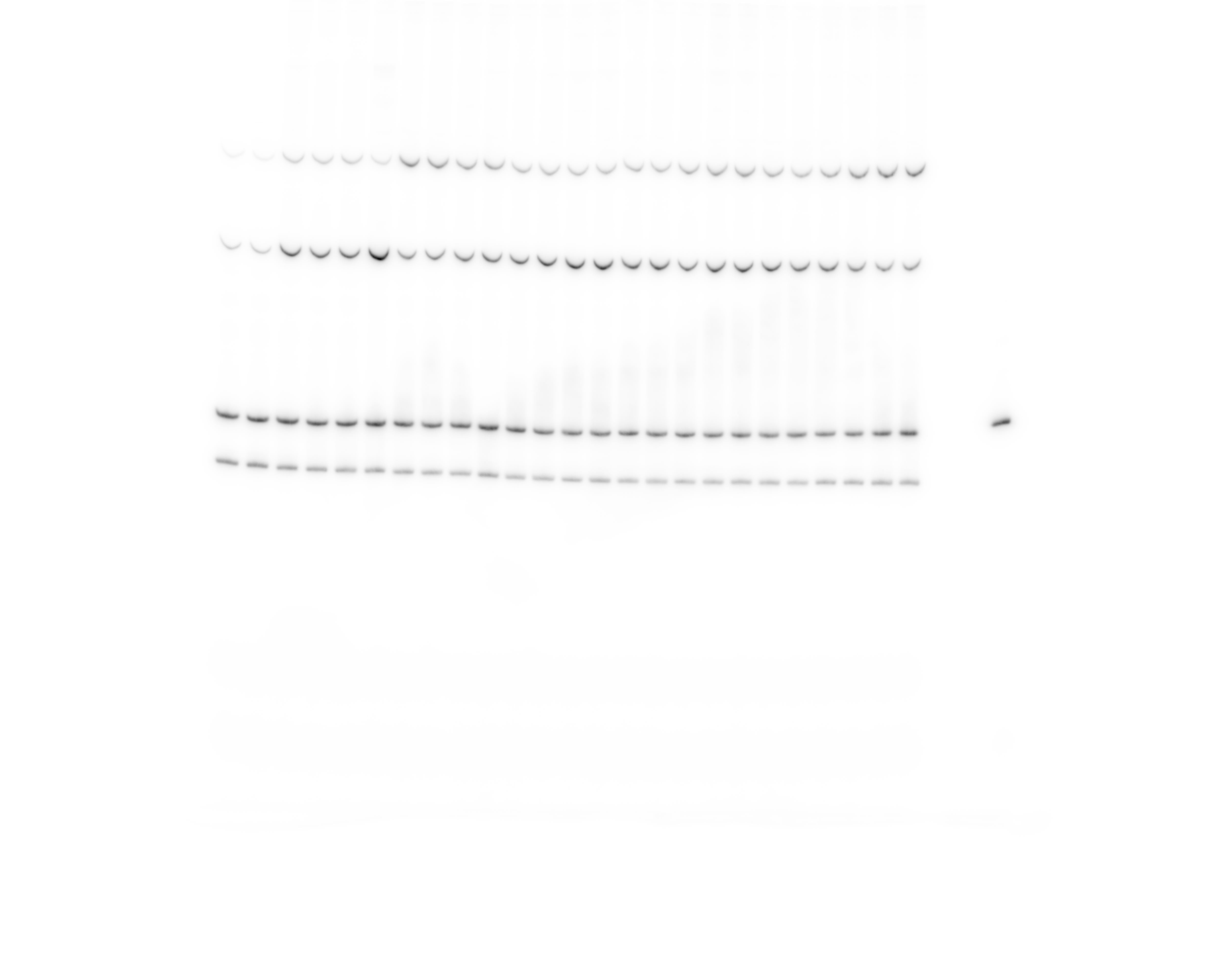

Supplement: Supplementary file 6 — Source Data [file 41467_2022_30668_MOESM6_ESM.zip › uncropped images/fig.1d.tif]

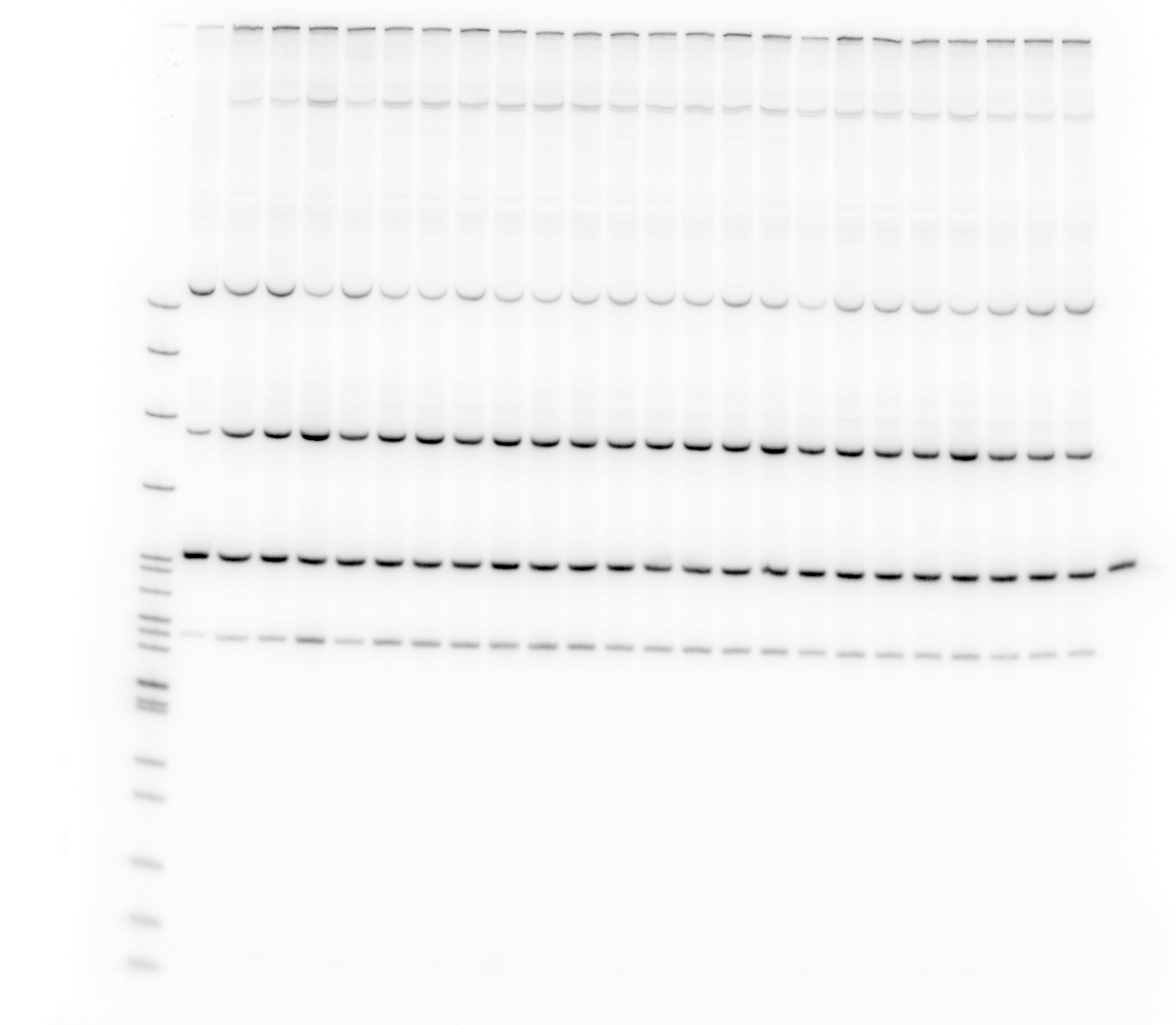

Supplement: Supplementary file 6 — Source Data [file 41467_2022_30668_MOESM6_ESM.zip › uncropped images/fig.1e.tif]

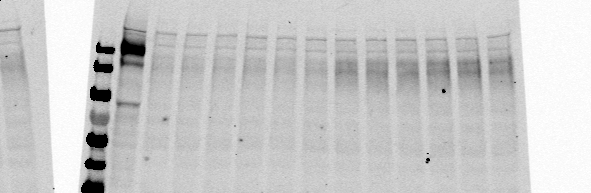

Supplement: Supplementary file 6 — Source Data [file 41467_2022_30668_MOESM6_ESM.zip › uncropped images/fig.3a CFTR.TIF]

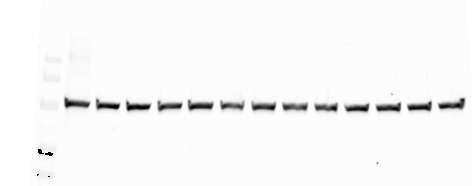

Supplement: Supplementary file 6 — Source Data [file 41467_2022_30668_MOESM6_ESM.zip › uncropped images/fig.3a NAKATPase.TIF]

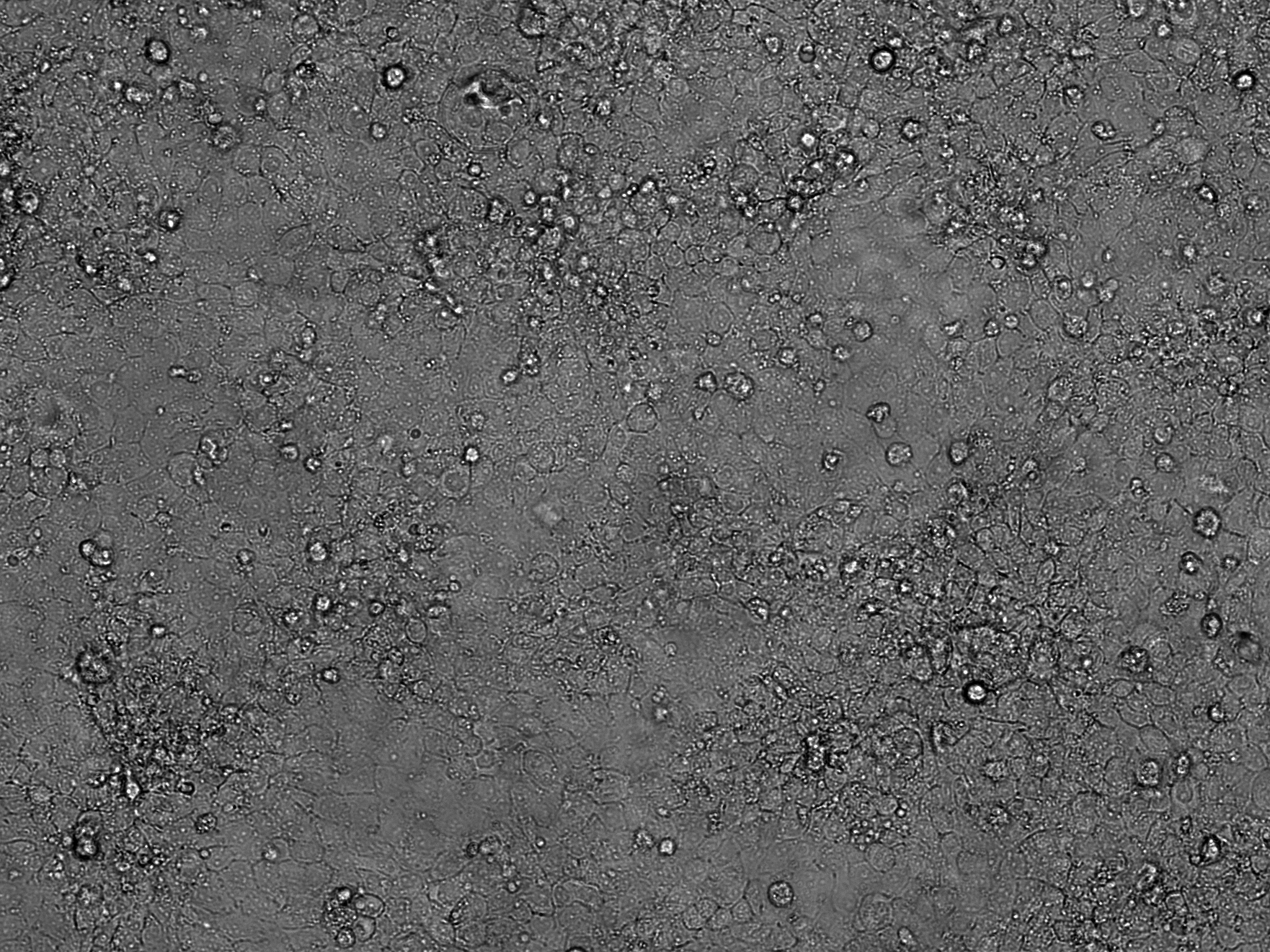

Supplement: Supplementary file 6 — Source Data [file 41467_2022_30668_MOESM6_ESM.zip › uncropped images/fig.4b p2a-w1282x-dox brightfield.tiff]

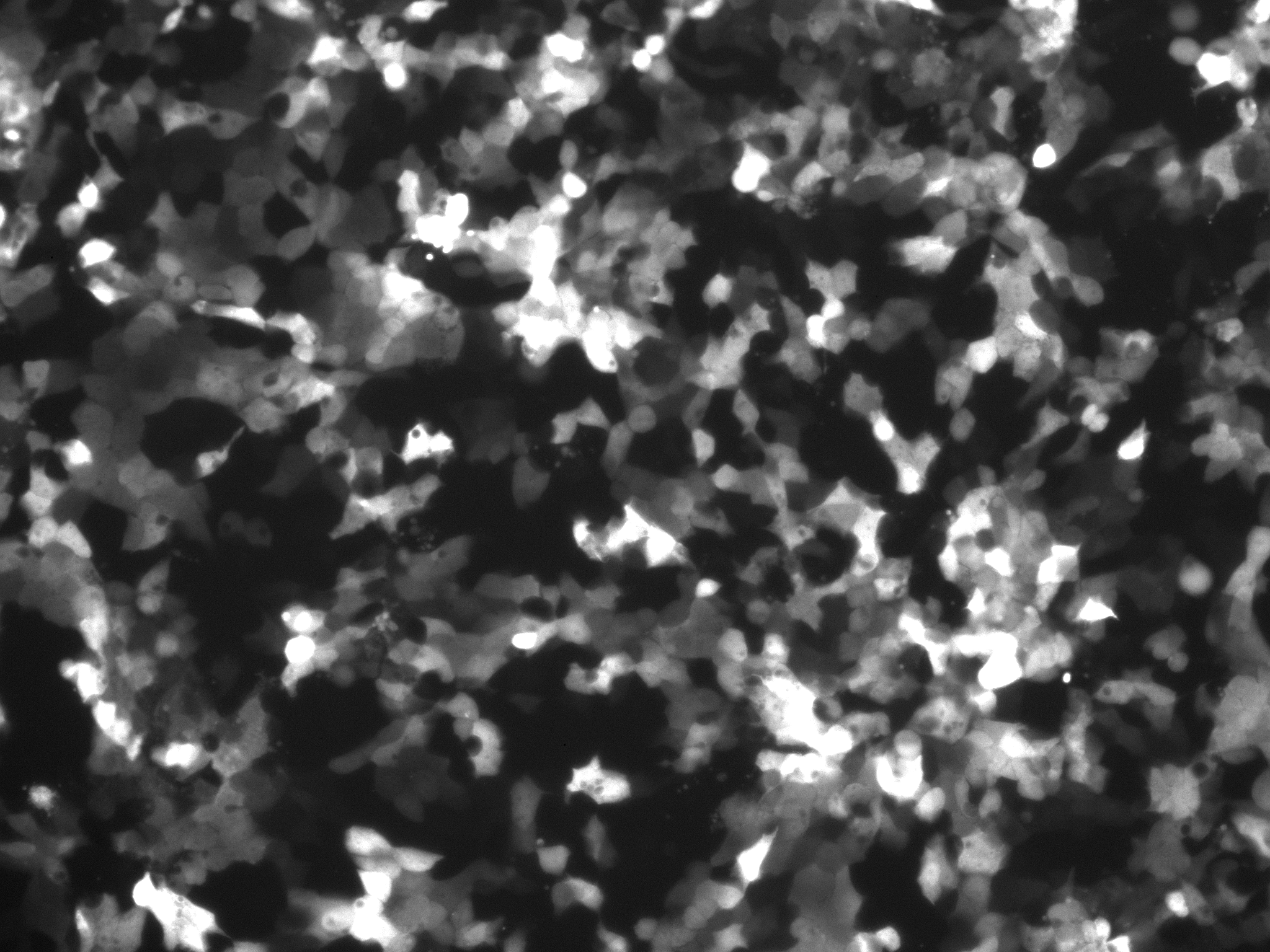

Supplement: Supplementary file 6 — Source Data [file 41467_2022_30668_MOESM6_ESM.zip › uncropped images/fig.4b p2a-w1282x-dox gfp.tiff]

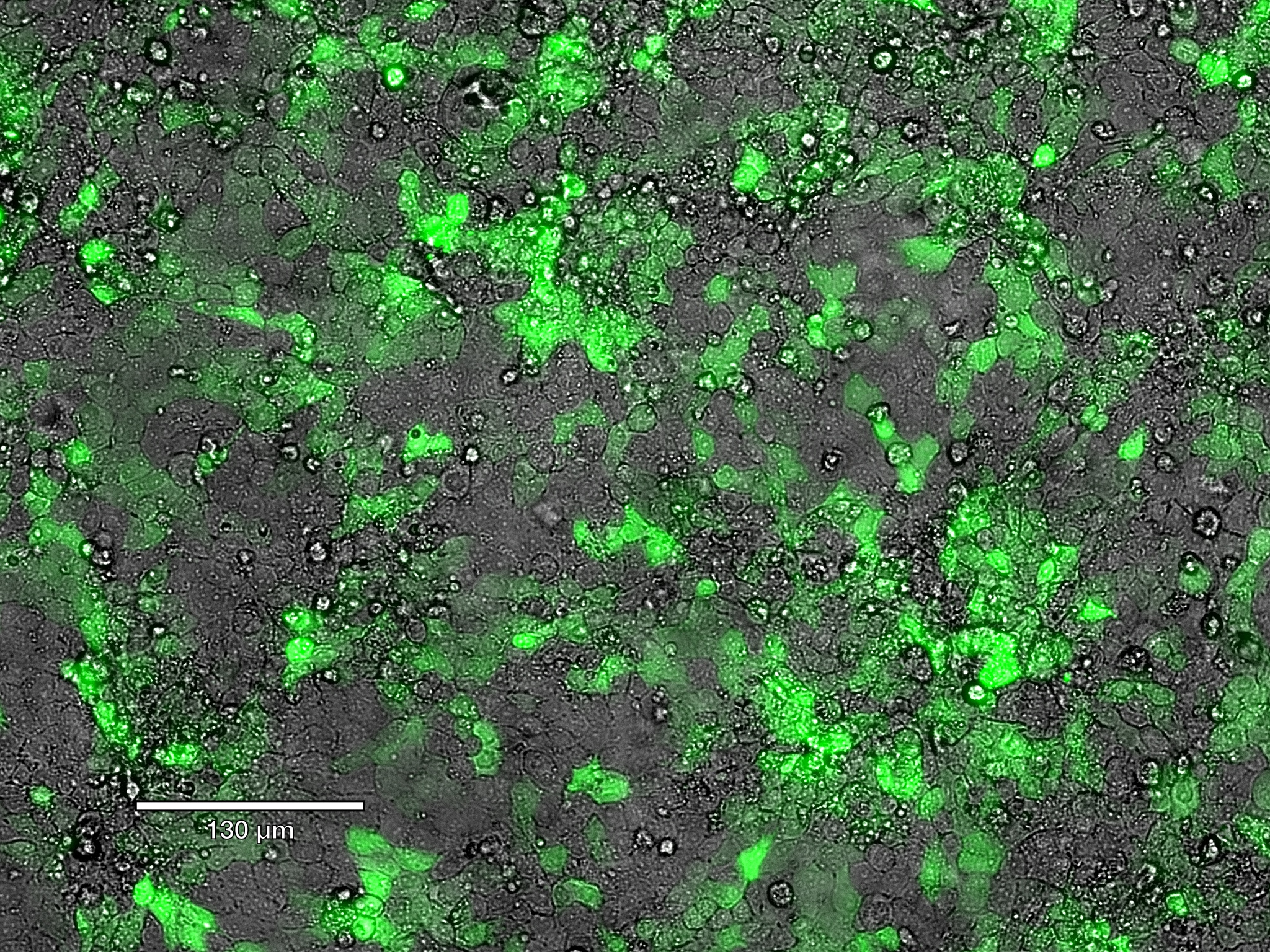

Supplement: Supplementary file 6 — Source Data [file 41467_2022_30668_MOESM6_ESM.zip › uncropped images/fig.4b p2a-w1282x-dox merge.jpg]

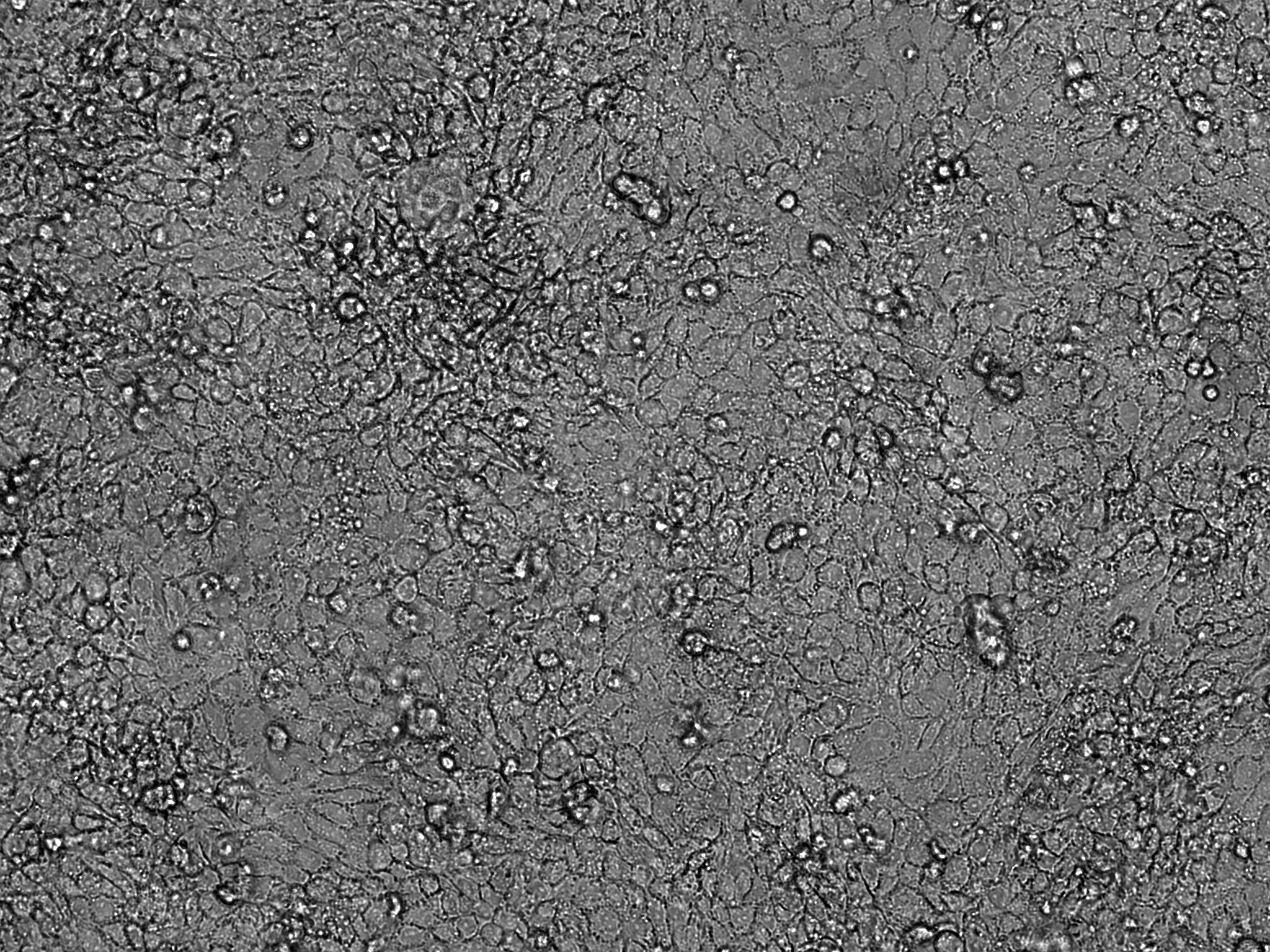

Supplement: Supplementary file 6 — Source Data [file 41467_2022_30668_MOESM6_ESM.zip › uncropped images/fig.4b p2a-w1282x-nodox brightfield.tiff]

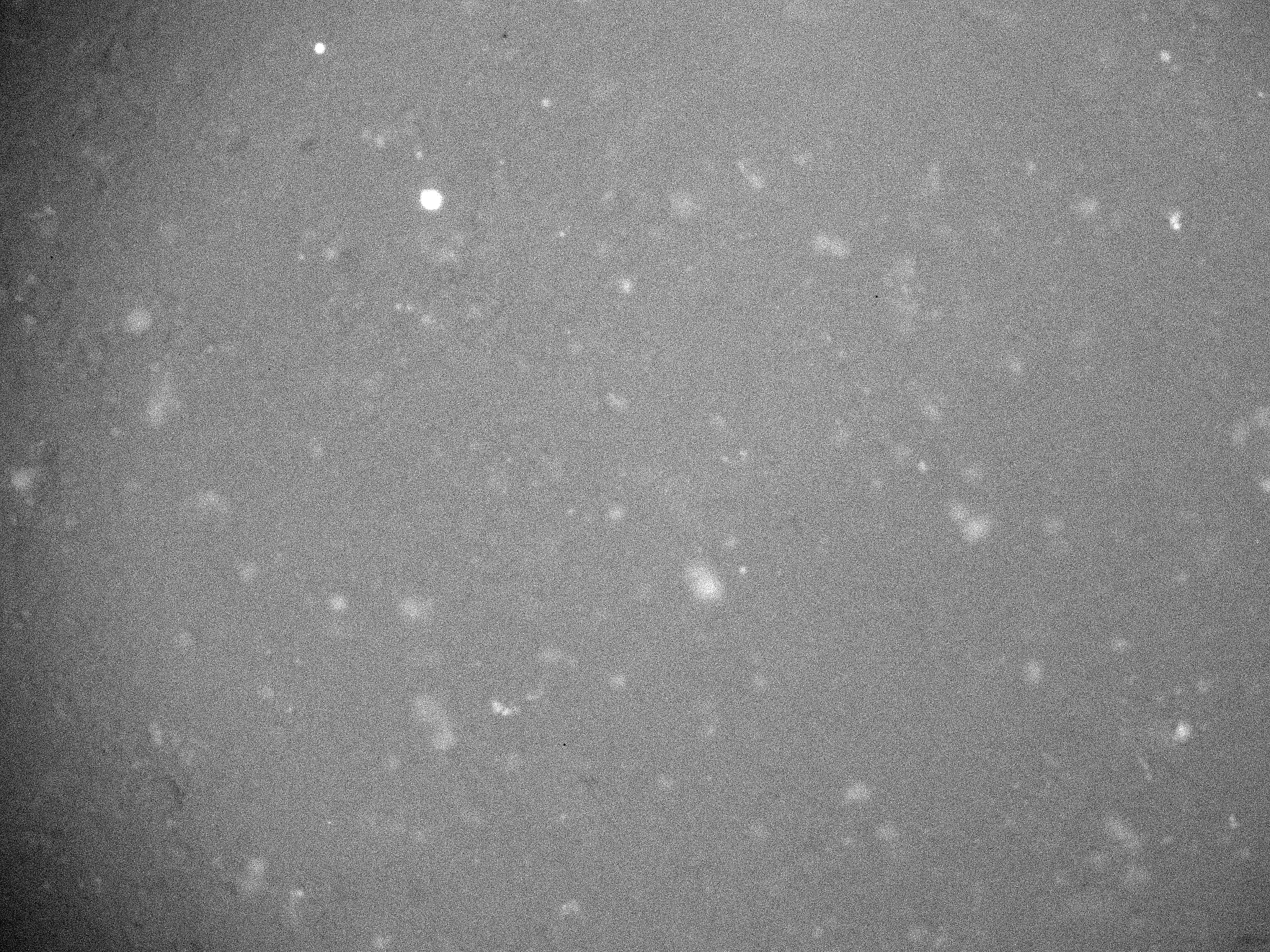

Supplement: Supplementary file 6 — Source Data [file 41467_2022_30668_MOESM6_ESM.zip › uncropped images/fig.4b p2a-w1282x-nodox gfp.tiff]

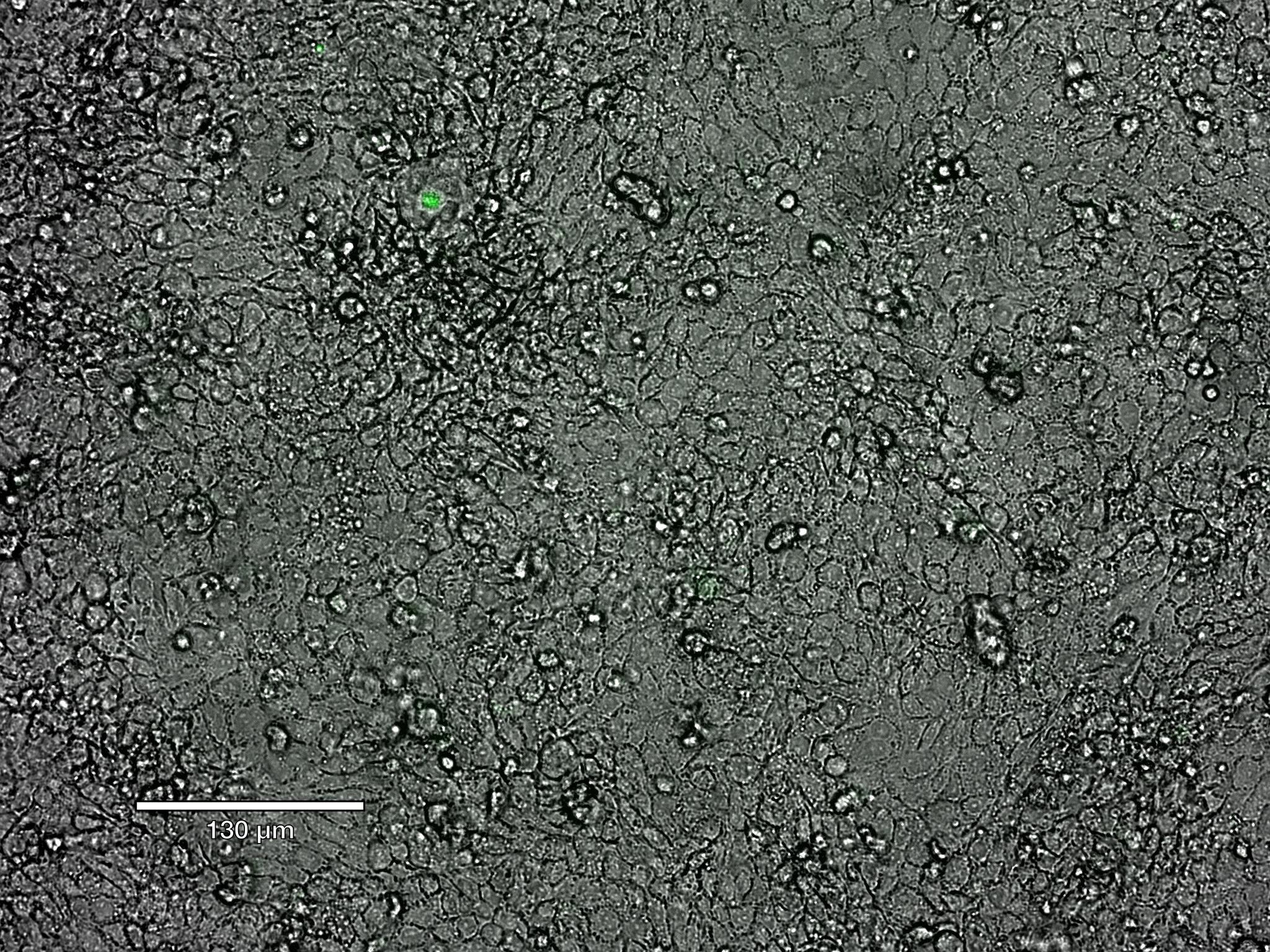

Supplement: Supplementary file 6 — Source Data [file 41467_2022_30668_MOESM6_ESM.zip › uncropped images/fig.4b p2a-w1282x-nodox merge.jpg]

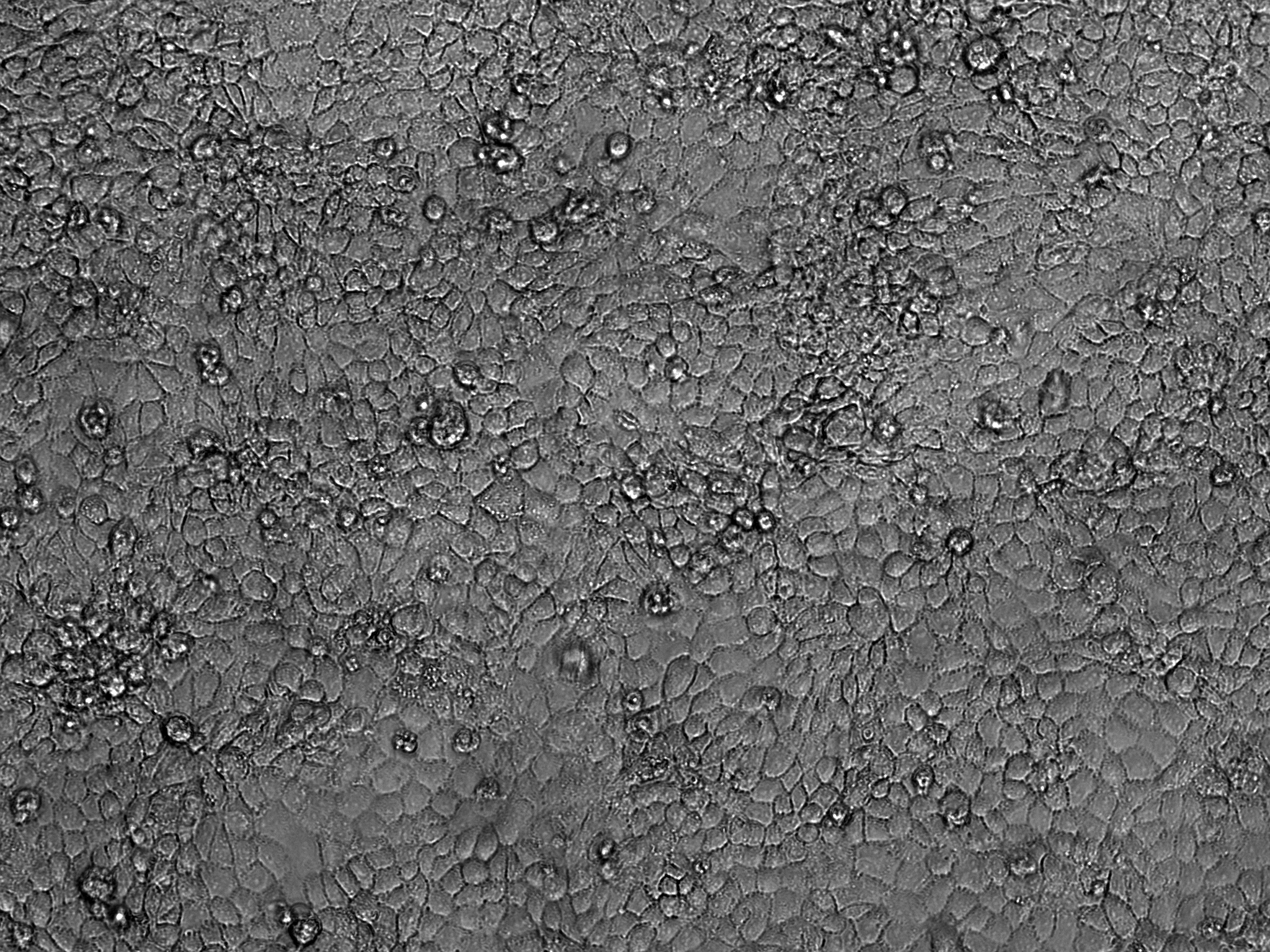

Supplement: Supplementary file 6 — Source Data [file 41467_2022_30668_MOESM6_ESM.zip › uncropped images/fig.4b p2a-wt-dox brightfield.tiff]

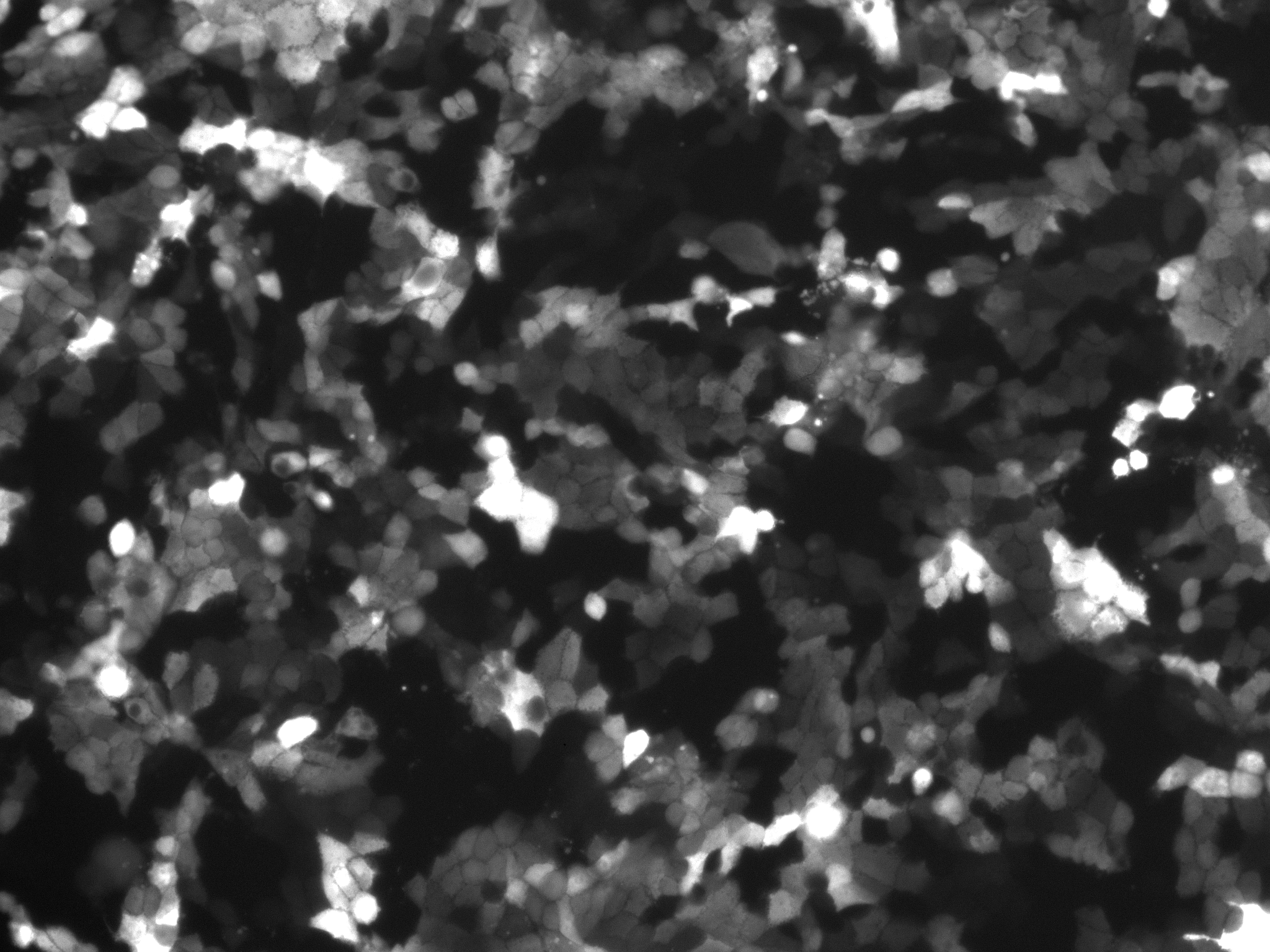

Supplement: Supplementary file 6 — Source Data [file 41467_2022_30668_MOESM6_ESM.zip › uncropped images/fig.4b p2a-wt-dox gfp.tiff]

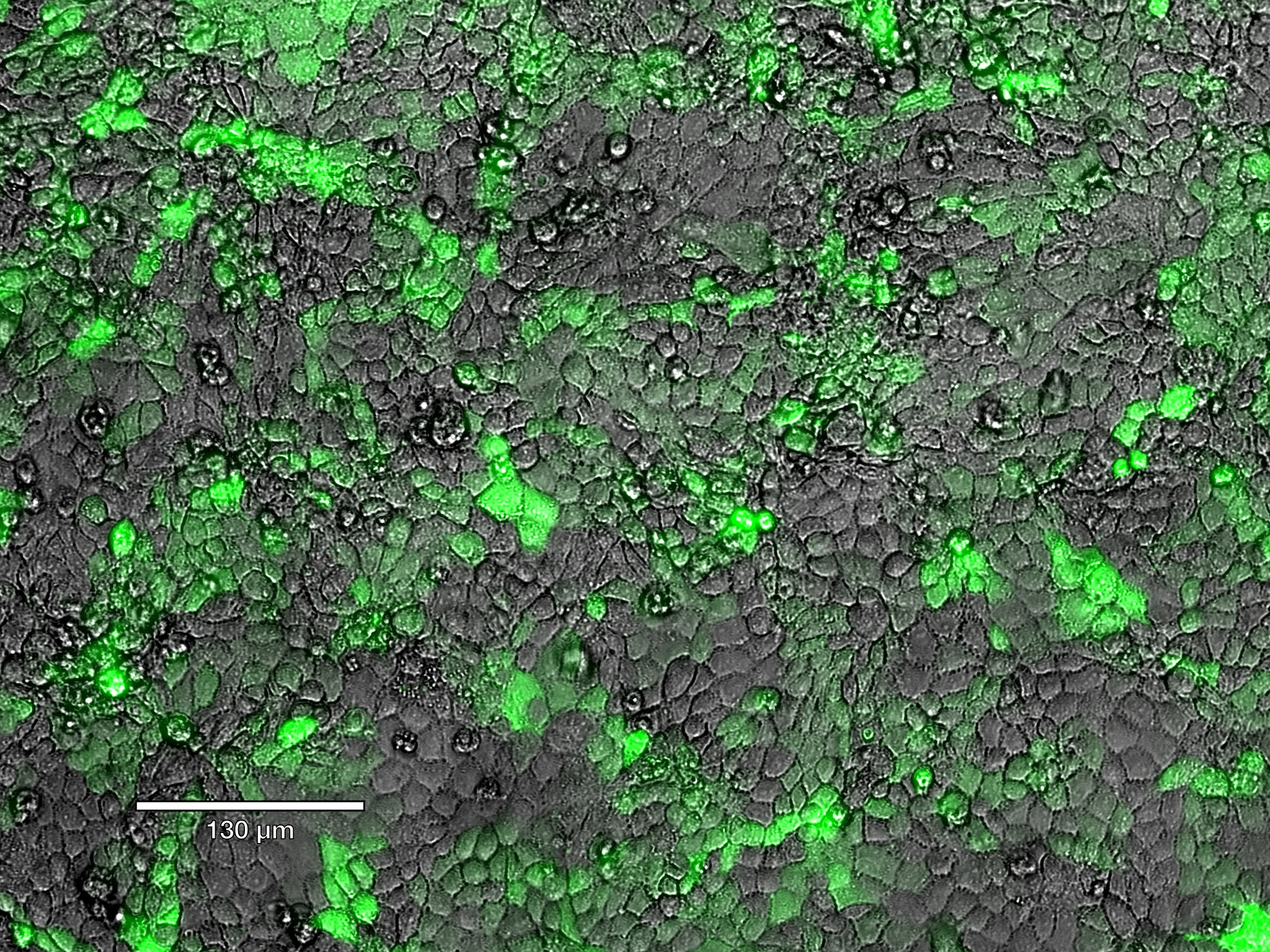

Supplement: Supplementary file 6 — Source Data [file 41467_2022_30668_MOESM6_ESM.zip › uncropped images/fig.4b p2a-wt-dox merge.jpg]

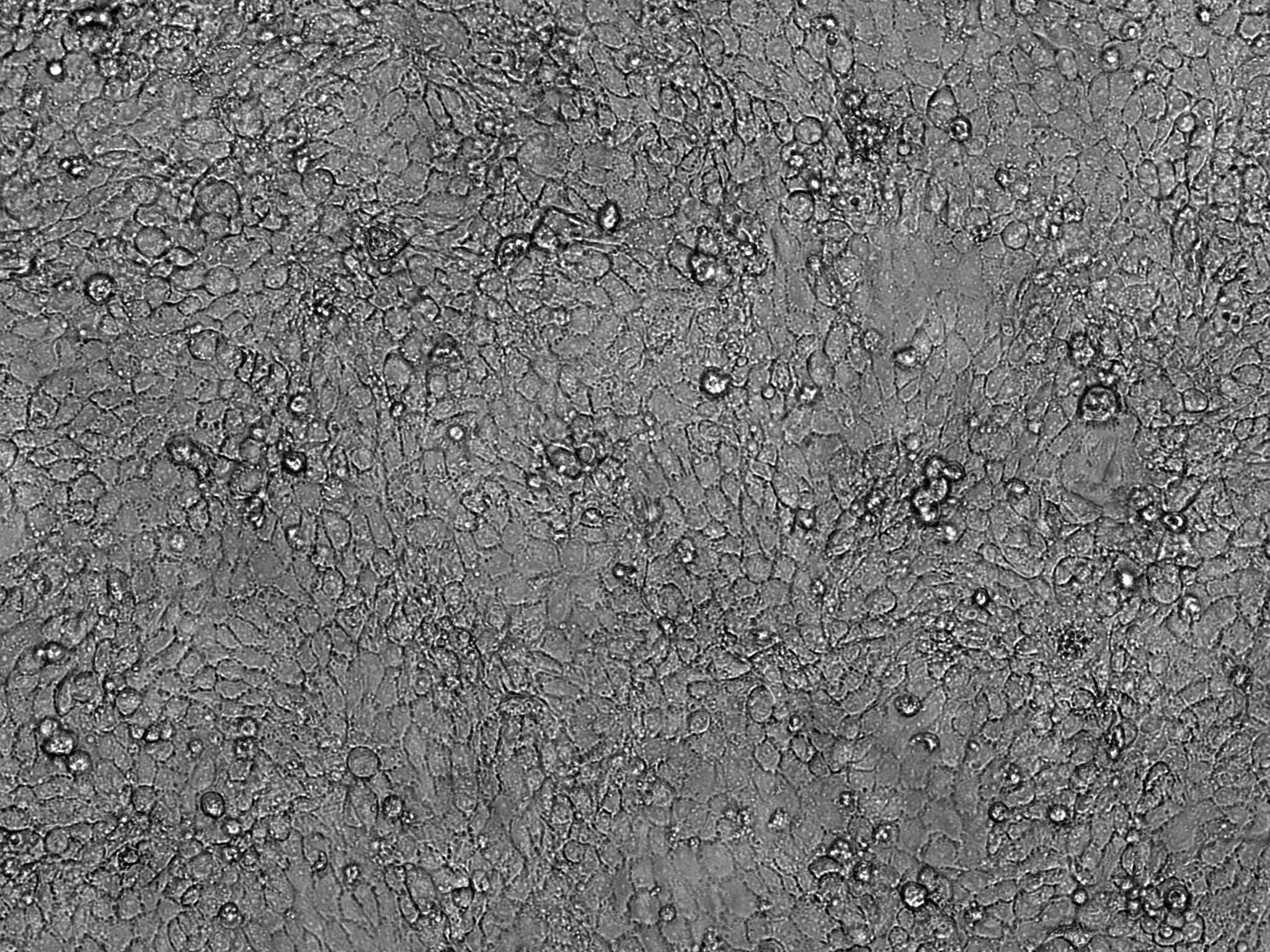

Supplement: Supplementary file 6 — Source Data [file 41467_2022_30668_MOESM6_ESM.zip › uncropped images/fig.4b p2a-wt-nodox brightfield.tiff]

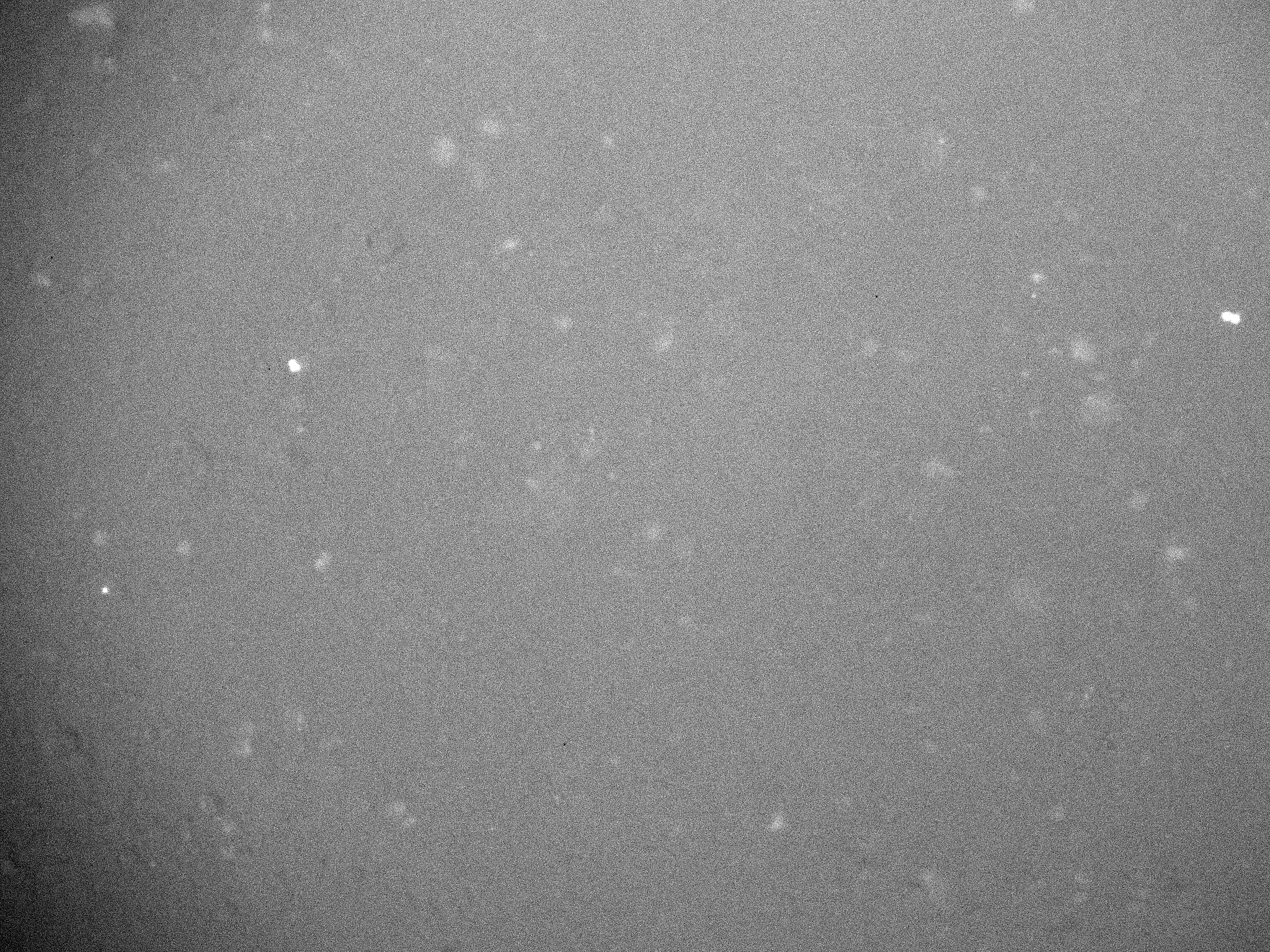

Supplement: Supplementary file 6 — Source Data [file 41467_2022_30668_MOESM6_ESM.zip › uncropped images/fig.4b p2a-wt-nodox gfp.tiff]

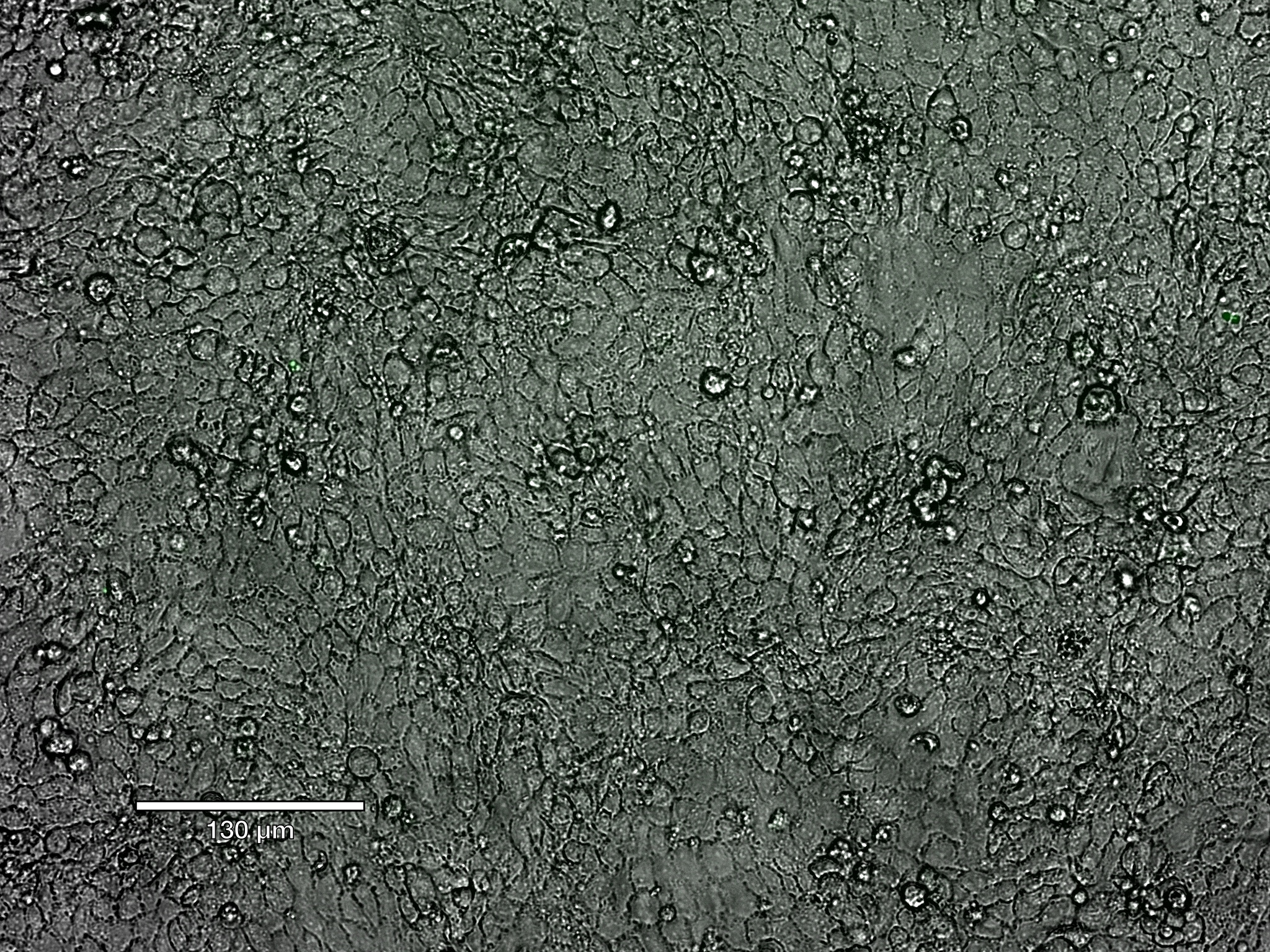

Supplement: Supplementary file 6 — Source Data [file 41467_2022_30668_MOESM6_ESM.zip › uncropped images/fig.4b p2a-wt-nodox merge.jpg]

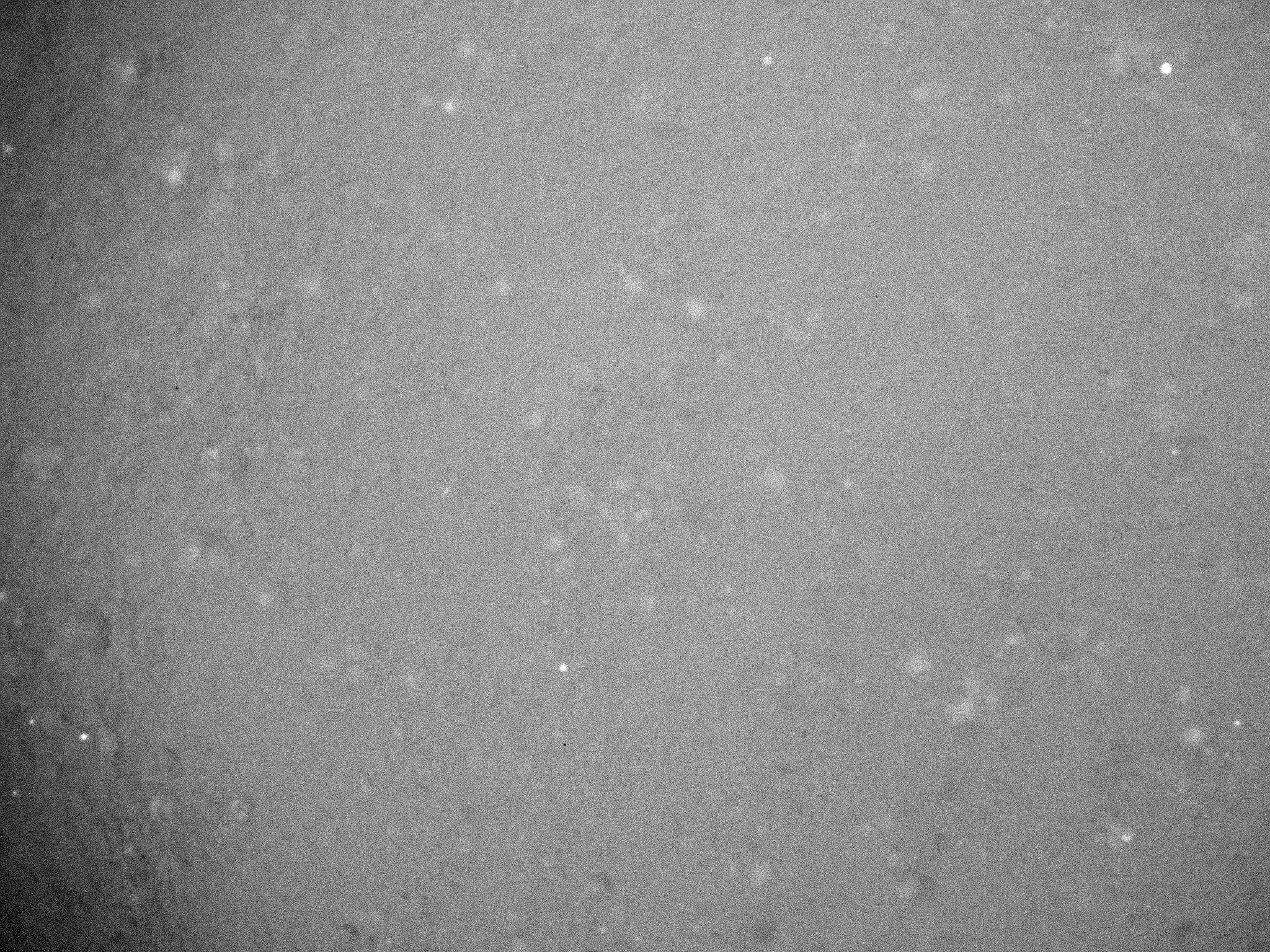

Supplement: Supplementary file 6 — Source Data [file 41467_2022_30668_MOESM6_ESM.zip › uncropped images/fig.4b parental GFP.tiff]

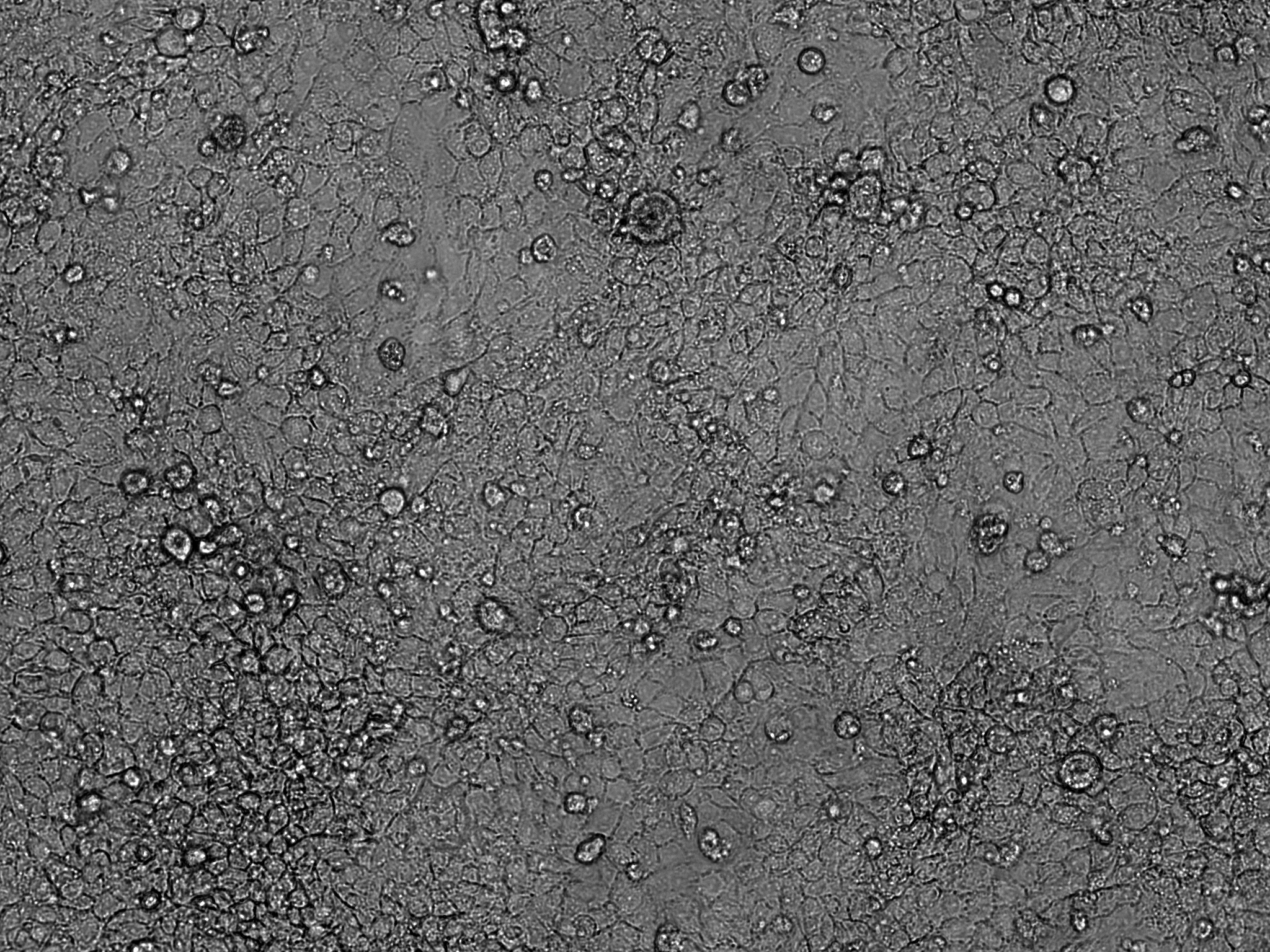

Supplement: Supplementary file 6 — Source Data [file 41467_2022_30668_MOESM6_ESM.zip › uncropped images/fig.4b parental bright field.tiff]

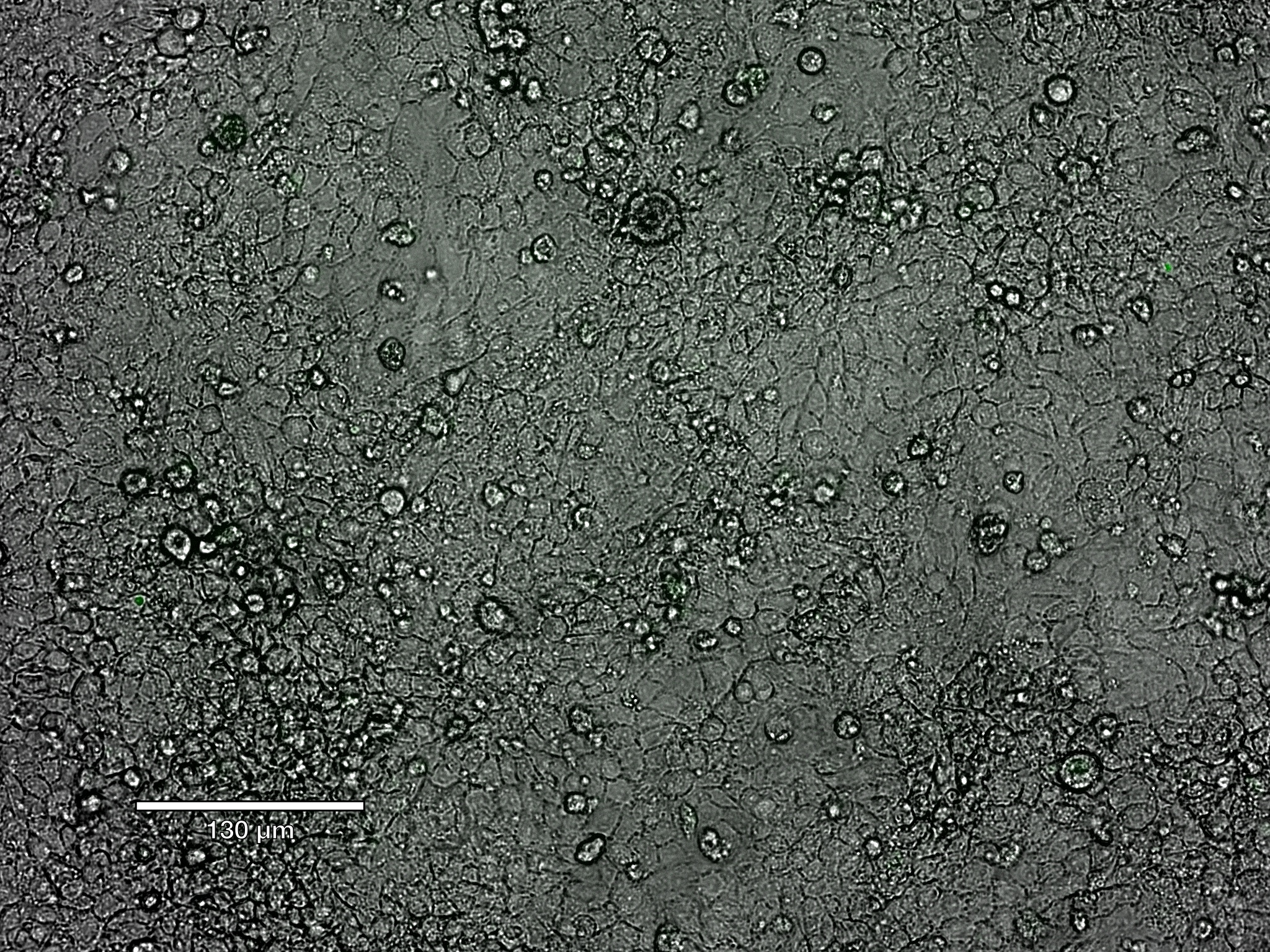

Supplement: Supplementary file 6 — Source Data [file 41467_2022_30668_MOESM6_ESM.zip › uncropped images/fig.4b parental merge.jpg]

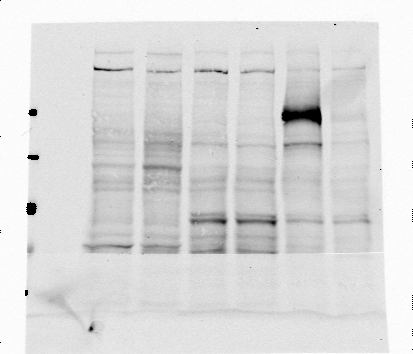

Supplement: Supplementary file 6 — Source Data [file 41467_2022_30668_MOESM6_ESM.zip › uncropped images/fig.S11a-CFTR.TIF]

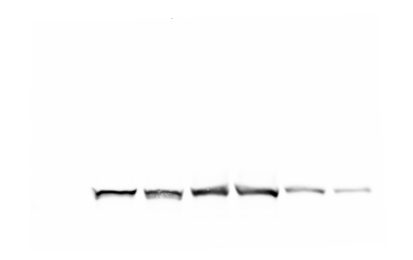

Supplement: Supplementary file 6 — Source Data [file 41467_2022_30668_MOESM6_ESM.zip › uncropped images/fig.S11a-NaKATPase.TIF]

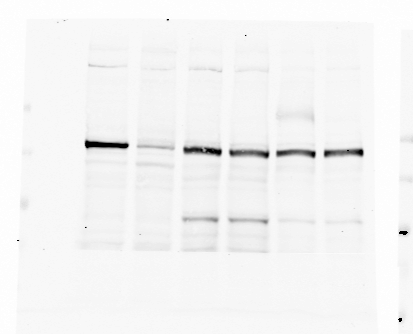

Supplement: Supplementary file 6 — Source Data [file 41467_2022_30668_MOESM6_ESM.zip › uncropped images/fig.S11a-UPF1.TIF]

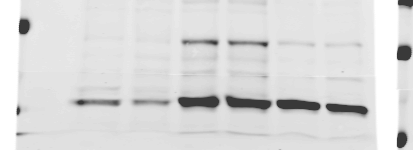

Supplement: Supplementary file 6 — Source Data [file 41467_2022_30668_MOESM6_ESM.zip › uncropped images/fig.S11a-tubulin.TIF]

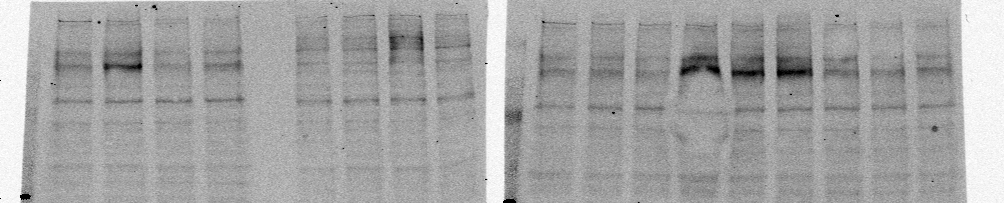

Supplement: Supplementary file 6 — Source Data [file 41467_2022_30668_MOESM6_ESM.zip › uncropped images/fig.S11b-CFTR representative.TIF]

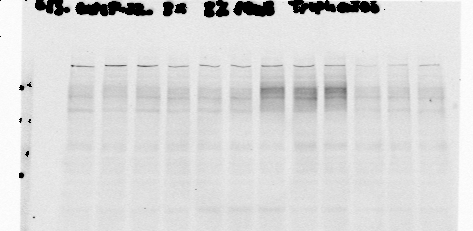

Supplement: Supplementary file 6 — Source Data [file 41467_2022_30668_MOESM6_ESM.zip › uncropped images/fig.S11b-CFTR.tif]

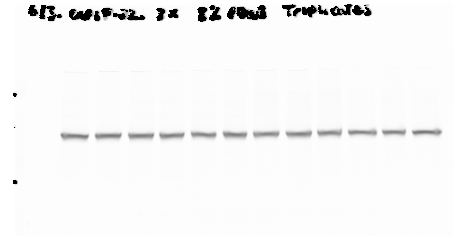

Supplement: Supplementary file 6 — Source Data [file 41467_2022_30668_MOESM6_ESM.zip › uncropped images/fig.S11b-NaKATPase (2).tif]

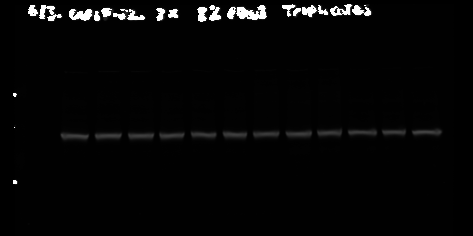

Supplement: Supplementary file 6 — Source Data [file 41467_2022_30668_MOESM6_ESM.zip › uncropped images/fig.S11b-NaKATPase.TIF]

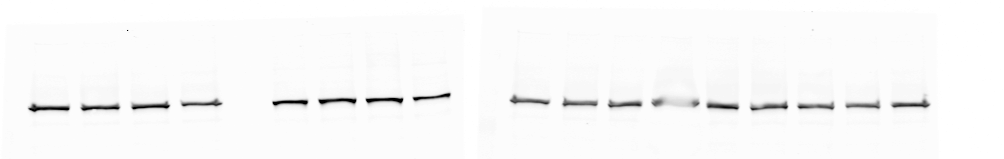

Supplement: Supplementary file 6 — Source Data [file 41467_2022_30668_MOESM6_ESM.zip › uncropped images/fig.S11b-representative NaKATPase.TIF]

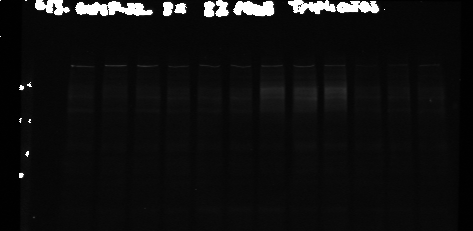

Supplement: Supplementary file 6 — Source Data [file 41467_2022_30668_MOESM6_ESM.zip › uncropped images/fig.S11b.TIF]

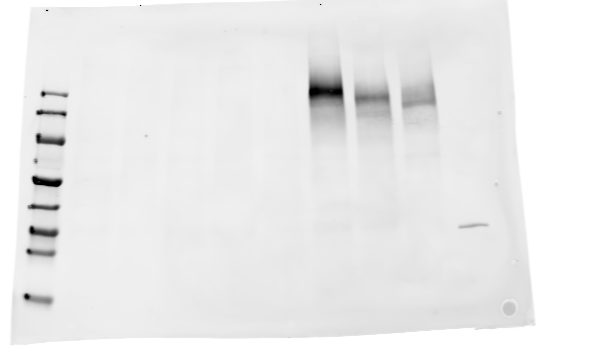

Supplement: Supplementary file 6 — Source Data [file 41467_2022_30668_MOESM6_ESM.zip › uncropped images/fig.S12-CFTR.tif]

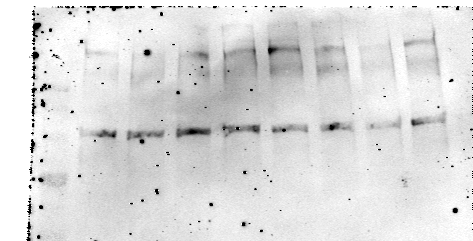

Supplement: Supplementary file 6 — Source Data [file 41467_2022_30668_MOESM6_ESM.zip › uncropped images/fig.S12-NaKATPase.TIF]

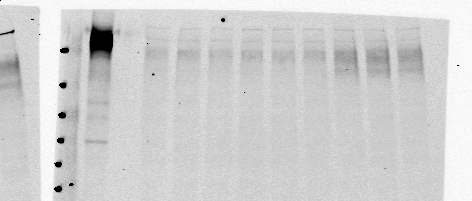

Supplement: Supplementary file 6 — Source Data [file 41467_2022_30668_MOESM6_ESM.zip › uncropped images/fig.S14a-CFTR.tif]

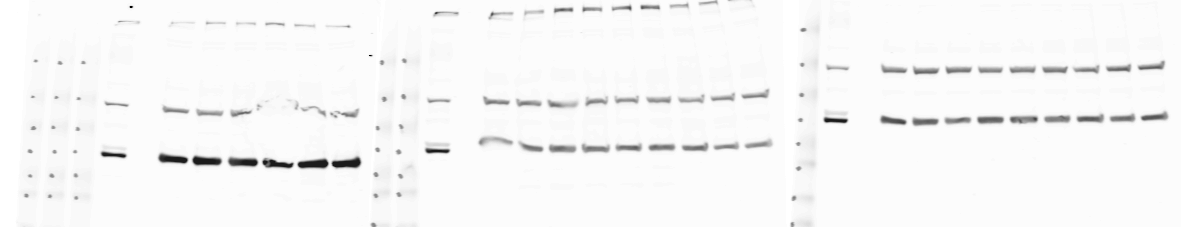

Supplement: Supplementary file 6 — Source Data [file 41467_2022_30668_MOESM6_ESM.zip › uncropped images/fig.S14a-NaKATPase and Tubulin.tif]

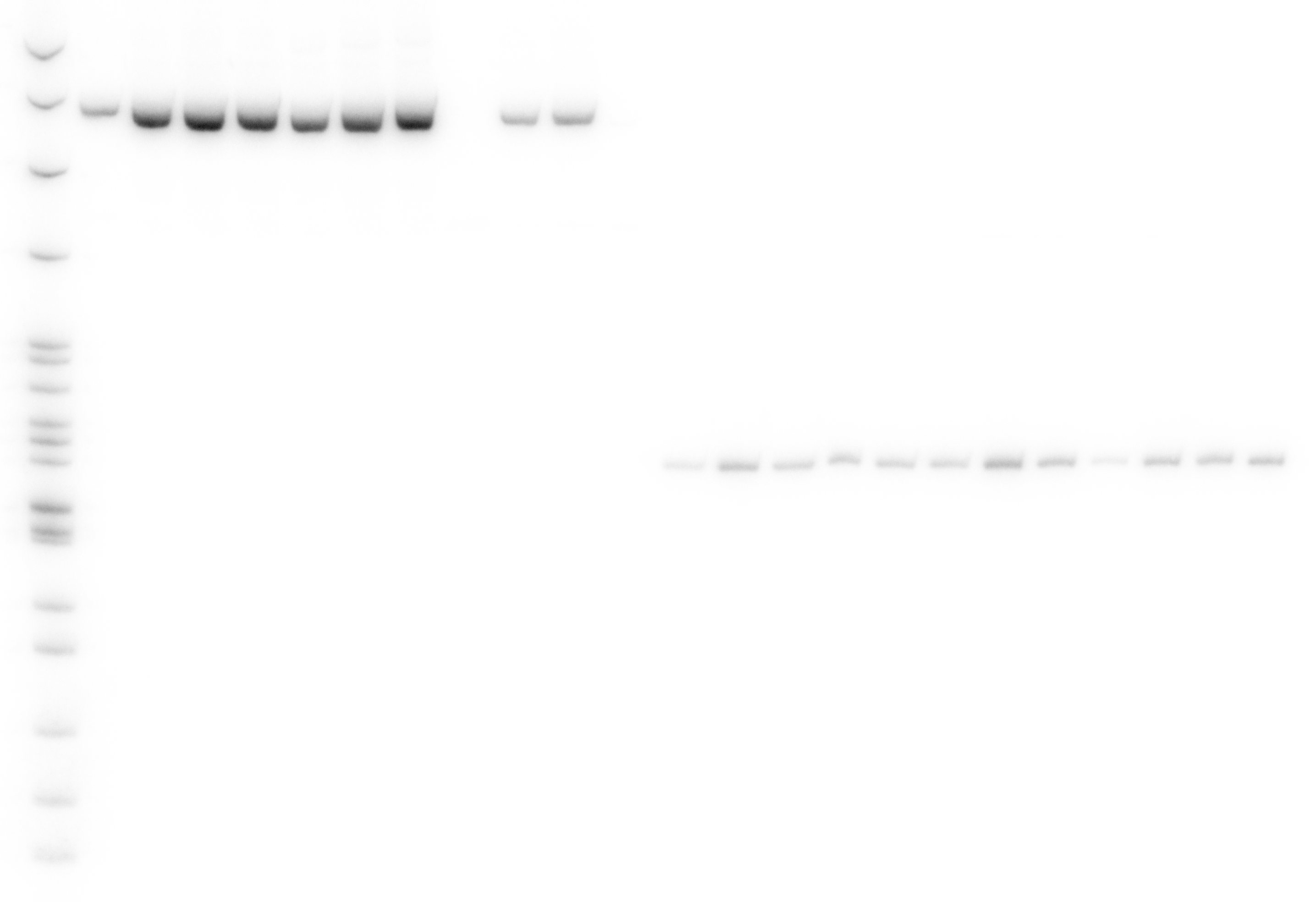

Supplement: Supplementary file 6 — Source Data [file 41467_2022_30668_MOESM6_ESM.zip › uncropped images/fig.S1b.tif]

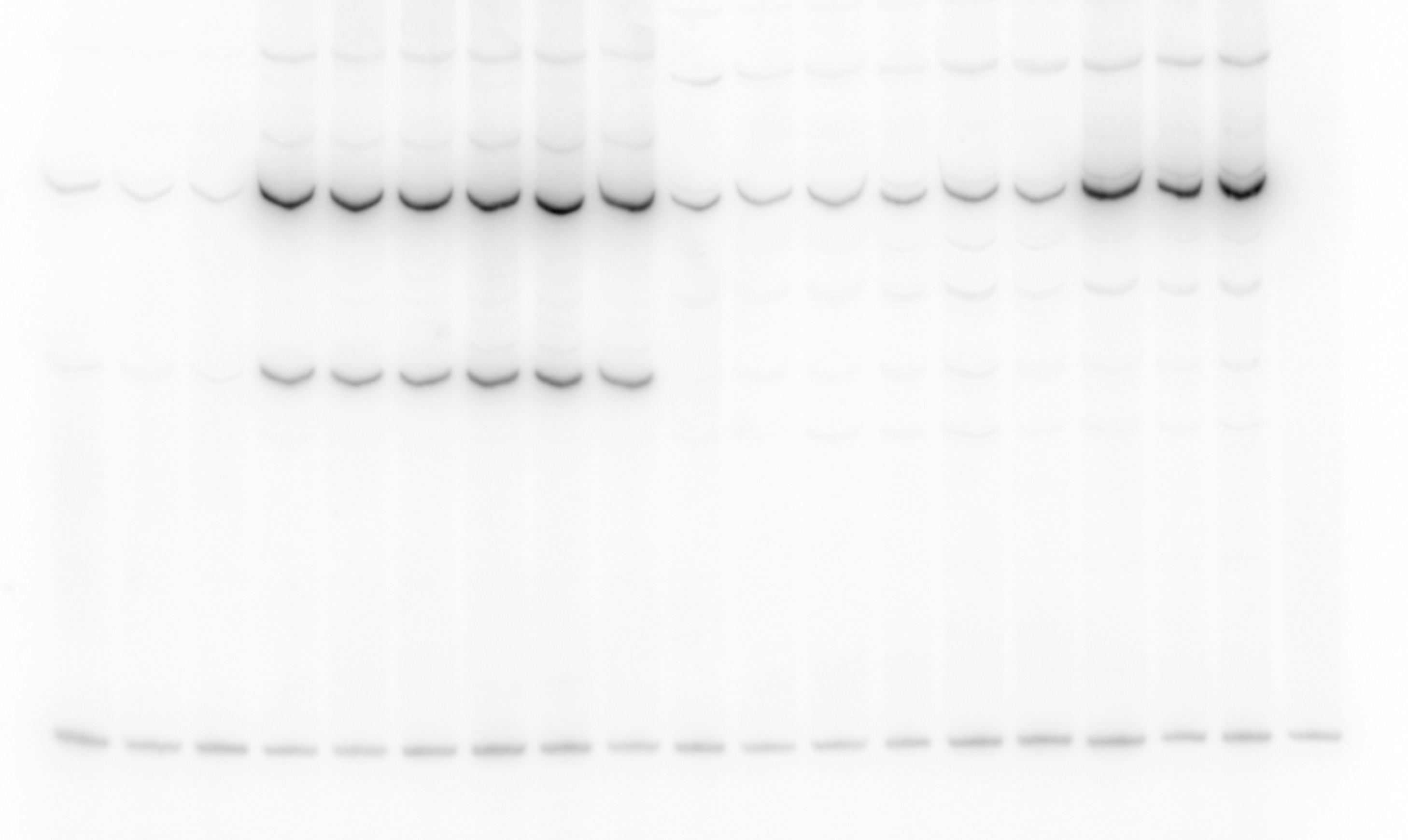

Supplement: Supplementary file 6 — Source Data [file 41467_2022_30668_MOESM6_ESM.zip › uncropped images/fig.S1c-d.tif]

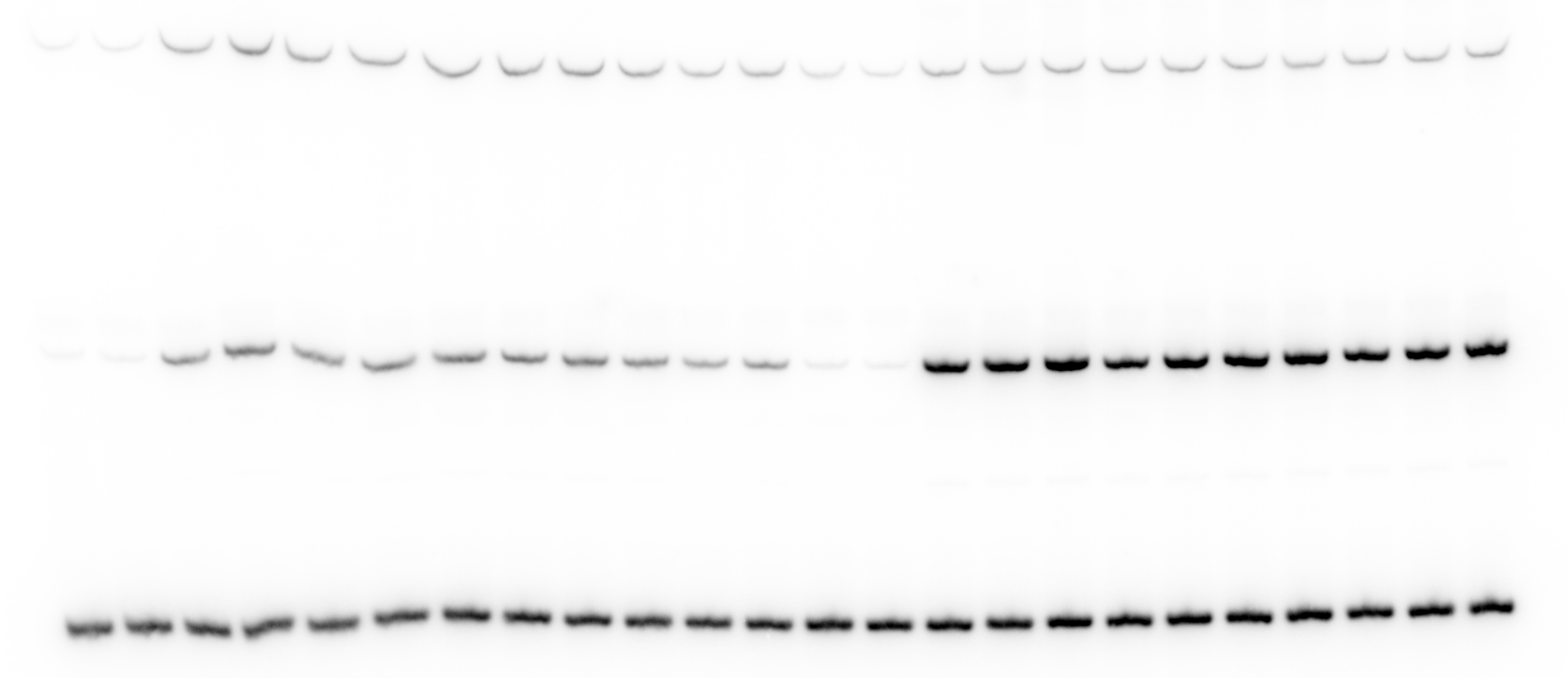

Supplement: Supplementary file 6 — Source Data [file 41467_2022_30668_MOESM6_ESM.zip › uncropped images/fig.S1e.tif]

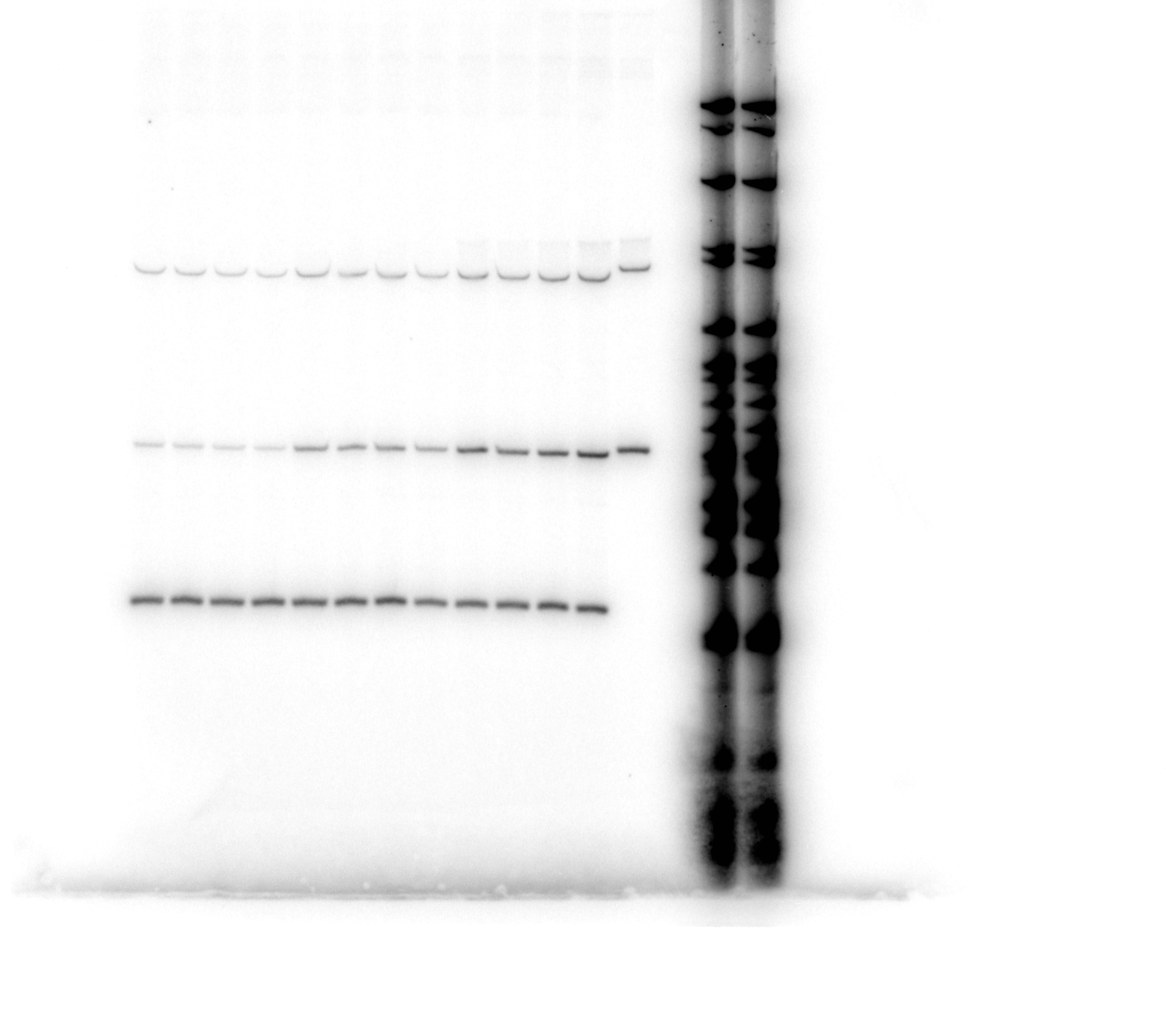

Supplement: Supplementary file 6 — Source Data [file 41467_2022_30668_MOESM6_ESM.zip › uncropped images/fig.S1f.tif]

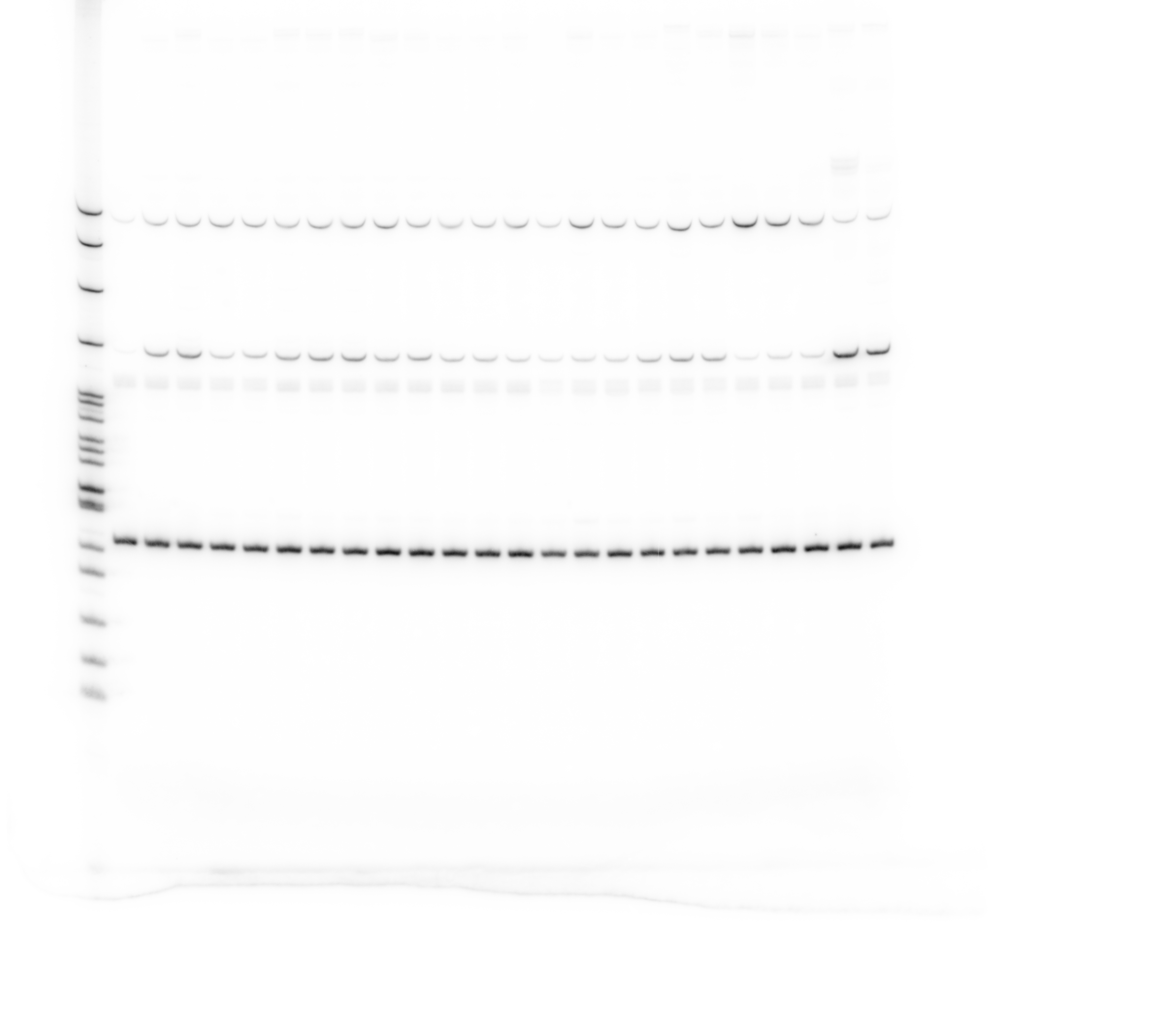

Supplement: Supplementary file 6 — Source Data [file 41467_2022_30668_MOESM6_ESM.zip › uncropped images/fig.S2a.tif]

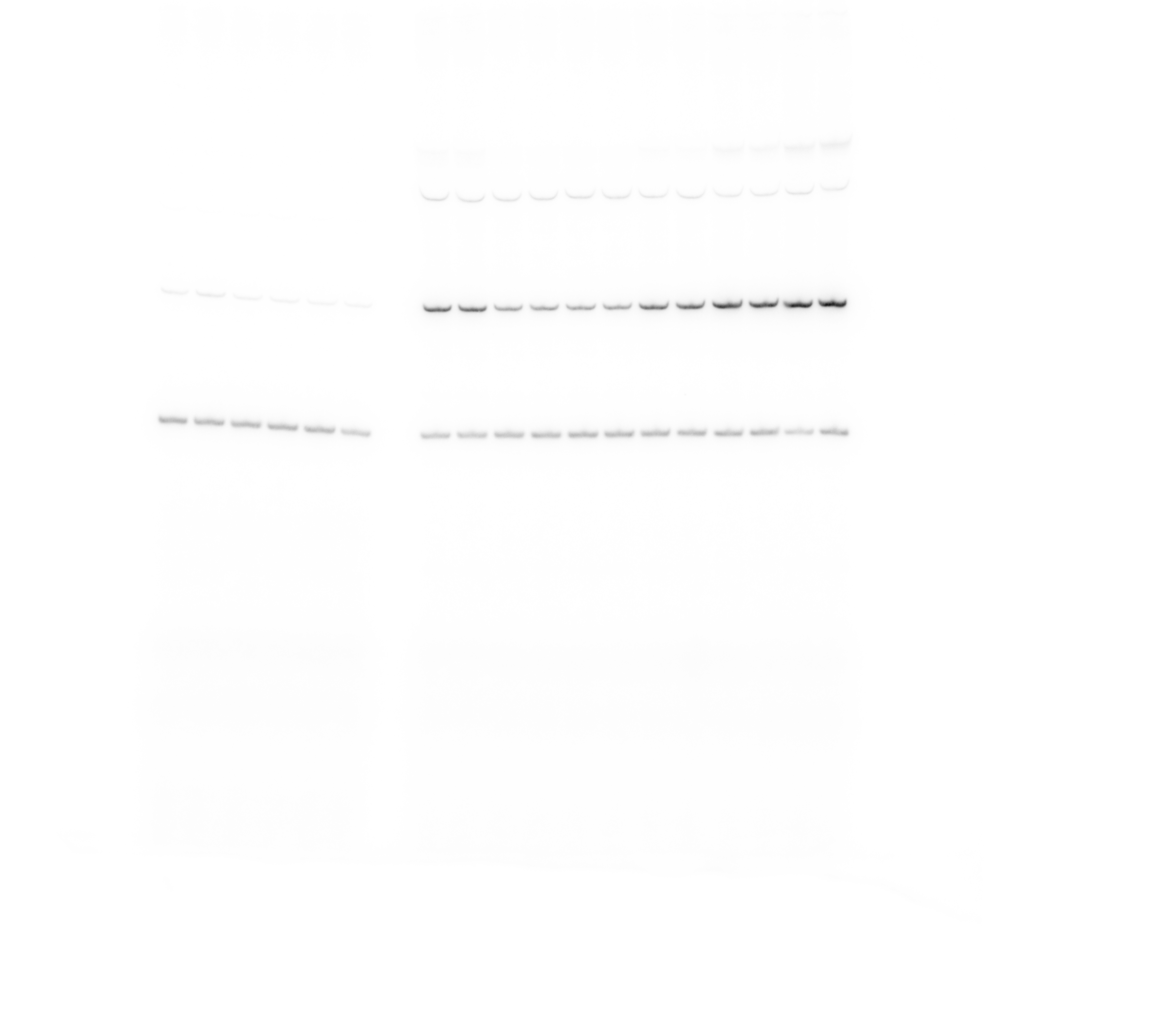

Supplement: Supplementary file 6 — Source Data [file 41467_2022_30668_MOESM6_ESM.zip › uncropped images/fig.S2c.tif]

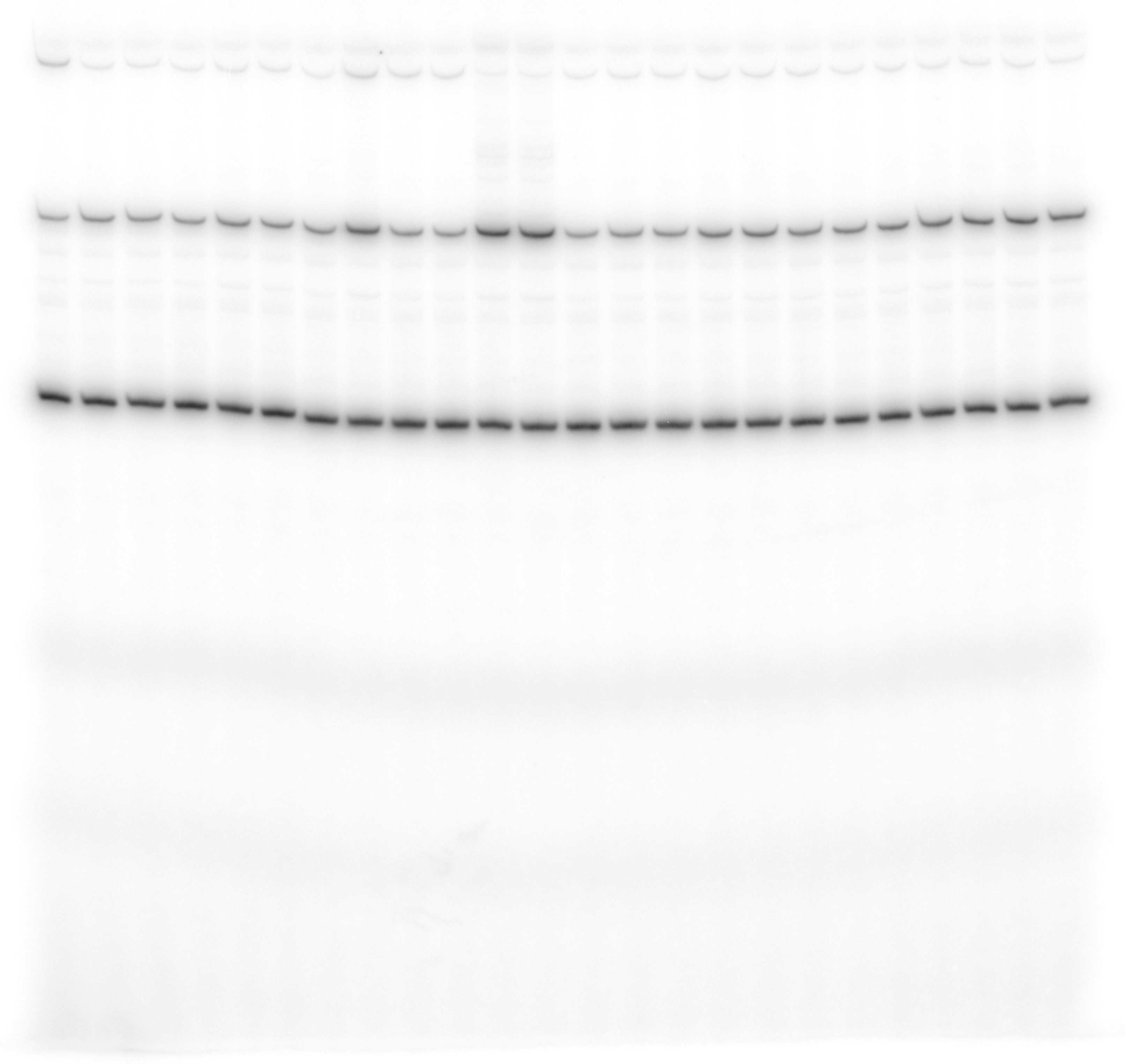

Supplement: Supplementary file 6 — Source Data [file 41467_2022_30668_MOESM6_ESM.zip › uncropped images/fig.S2d.tif]

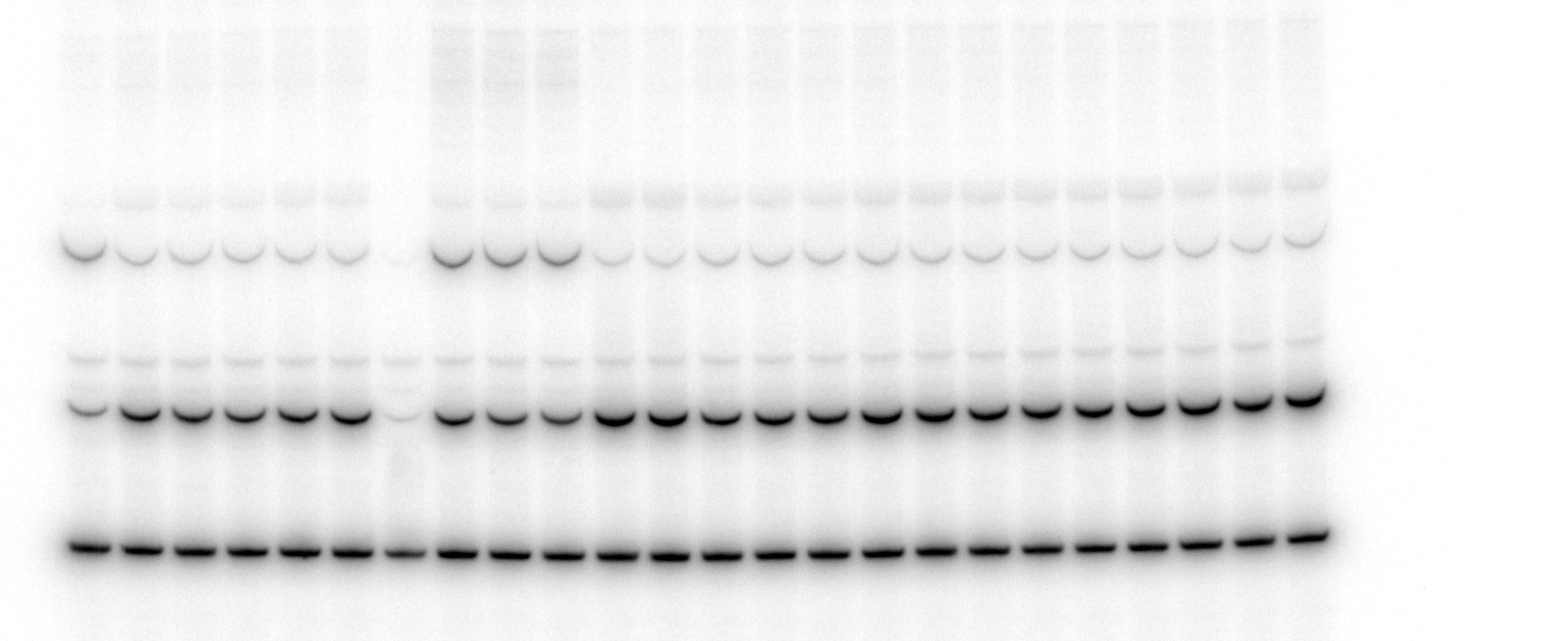

Supplement: Supplementary file 6 — Source Data [file 41467_2022_30668_MOESM6_ESM.zip › uncropped images/fig.S2e.tif]

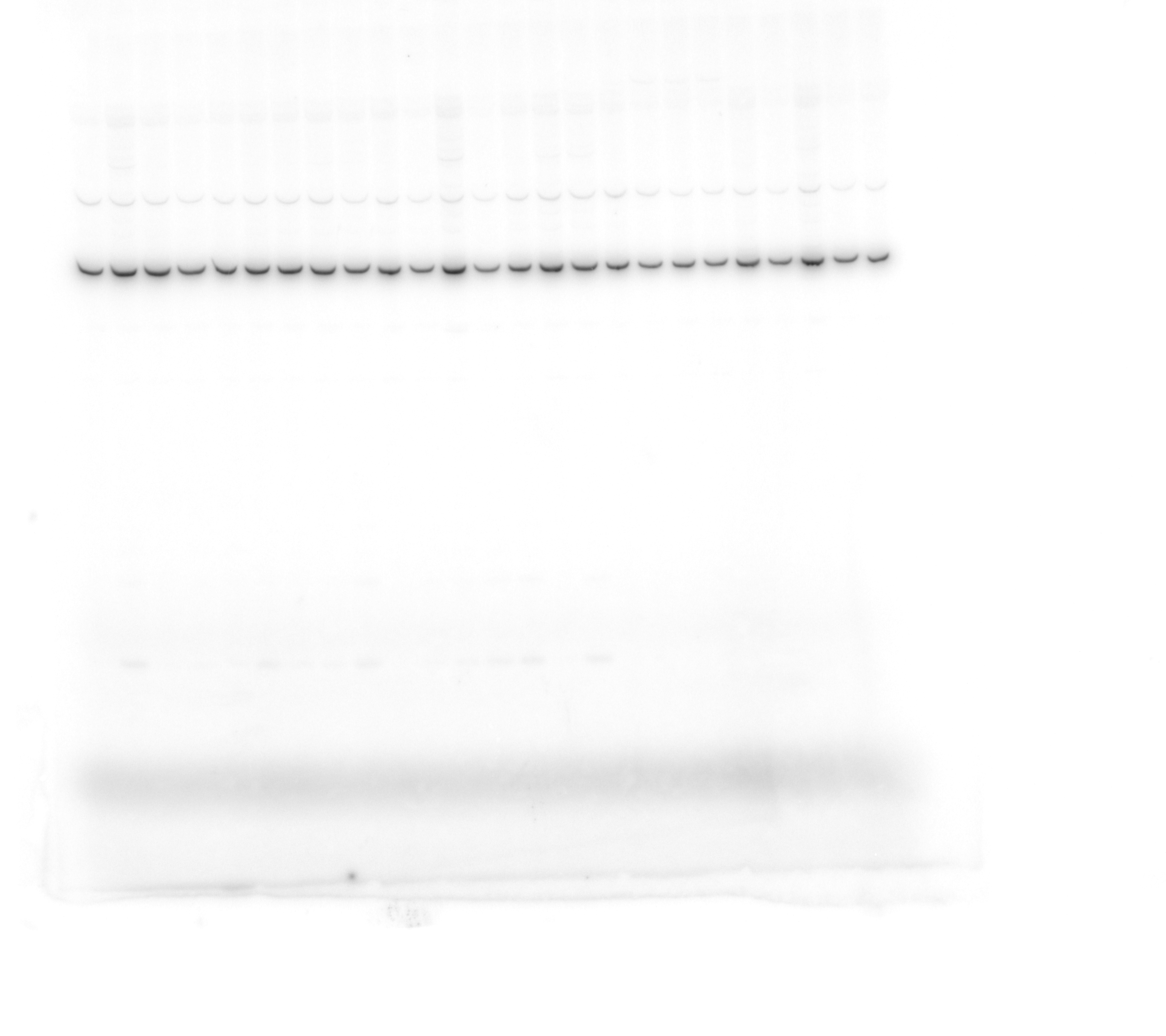

Supplement: Supplementary file 6 — Source Data [file 41467_2022_30668_MOESM6_ESM.zip › uncropped images/fig.S7a bottom panel.tif]

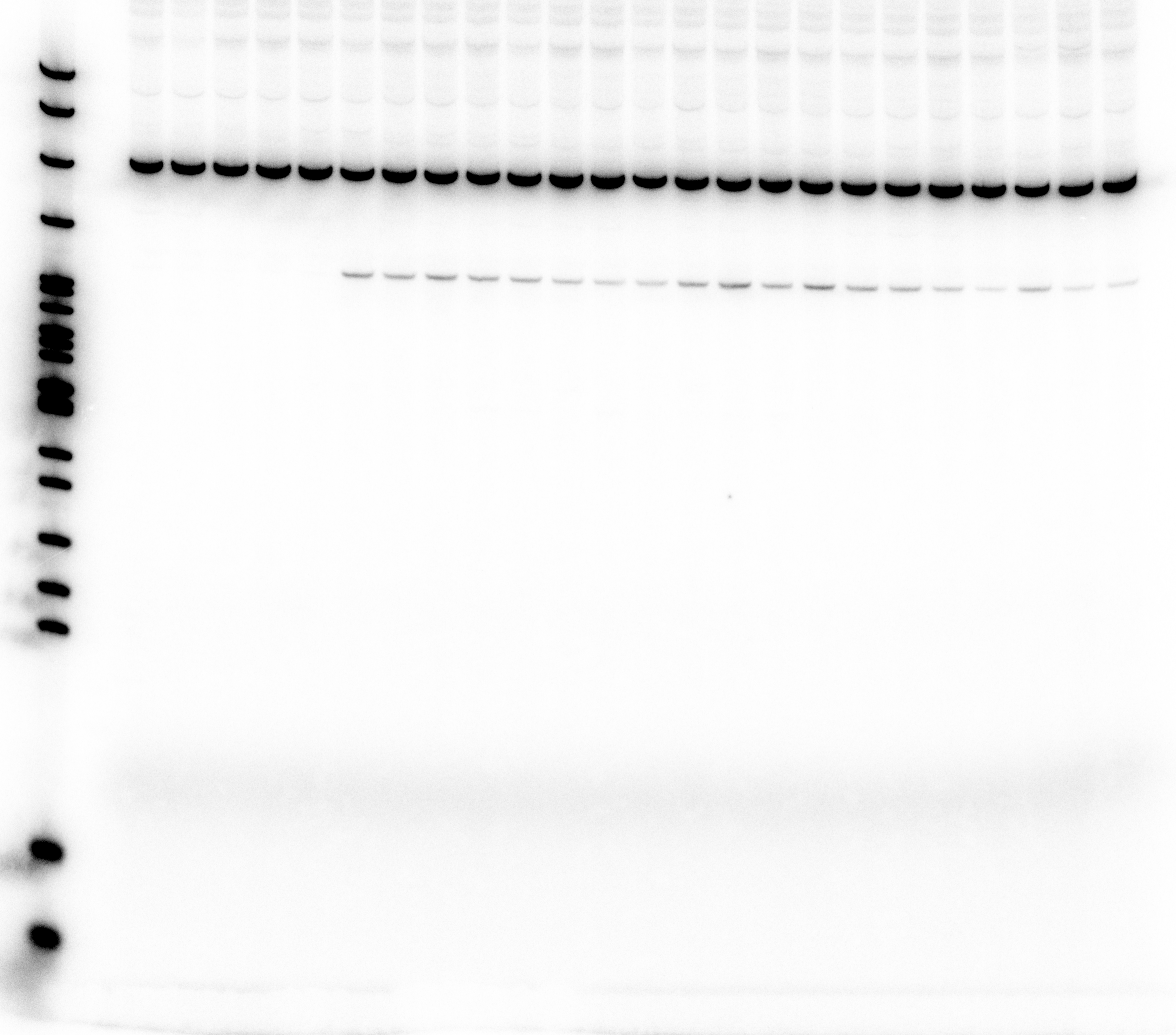

Supplement: Supplementary file 6 — Source Data [file 41467_2022_30668_MOESM6_ESM.zip › uncropped images/fig.S7a middle panel.tif]

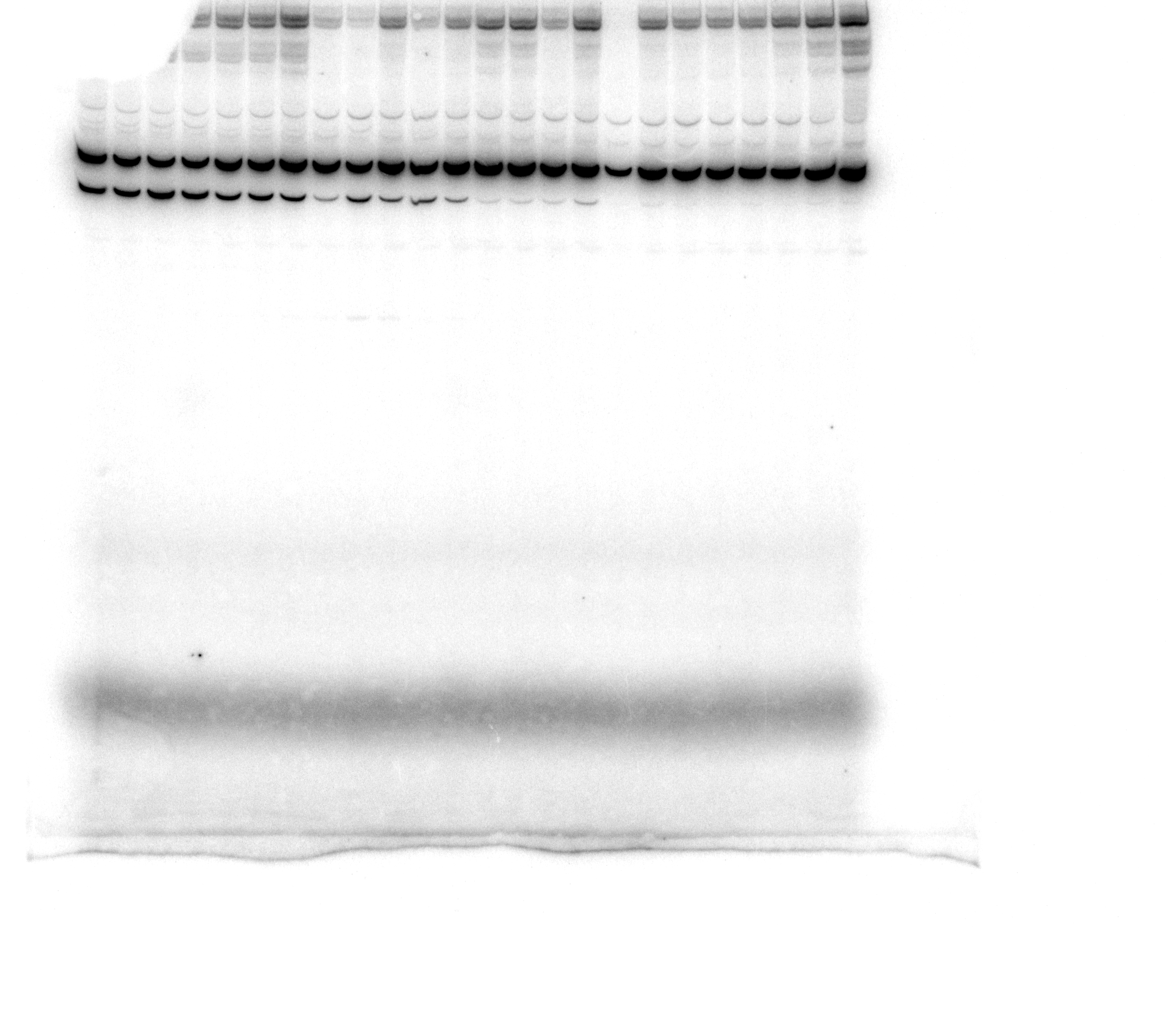

Supplement: Supplementary file 6 — Source Data [file 41467_2022_30668_MOESM6_ESM.zip › uncropped images/fig.S7a top panel.tif]

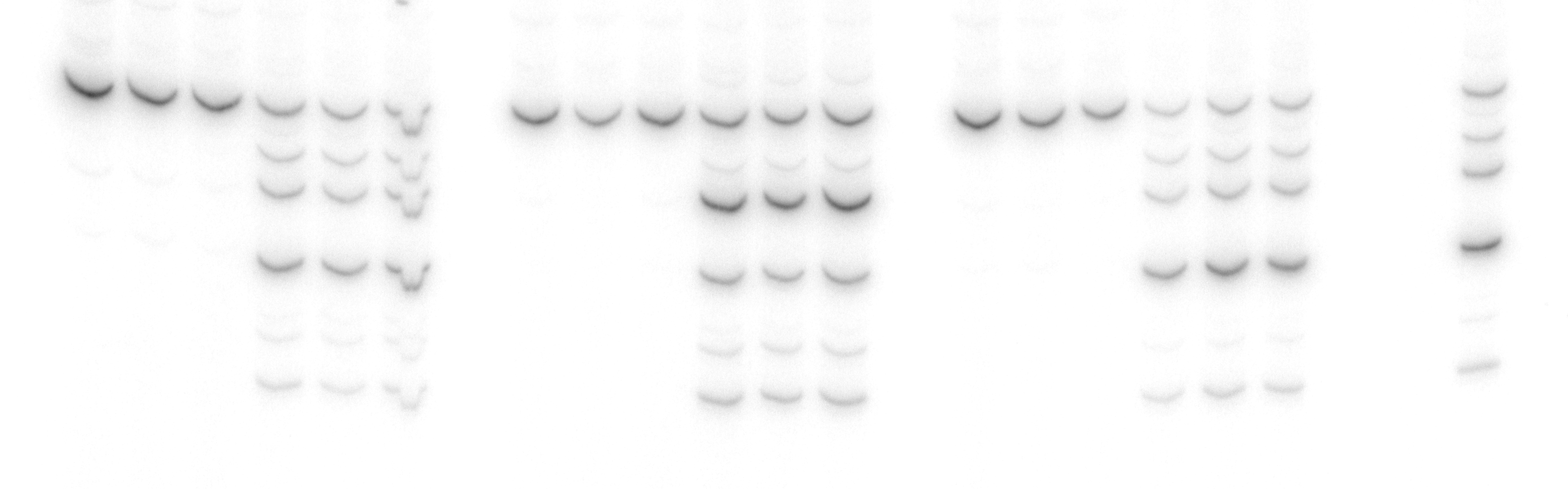

Supplement: Supplementary file 6 — Source Data [file 41467_2022_30668_MOESM6_ESM.zip › uncropped images/fig.S9a.tif]

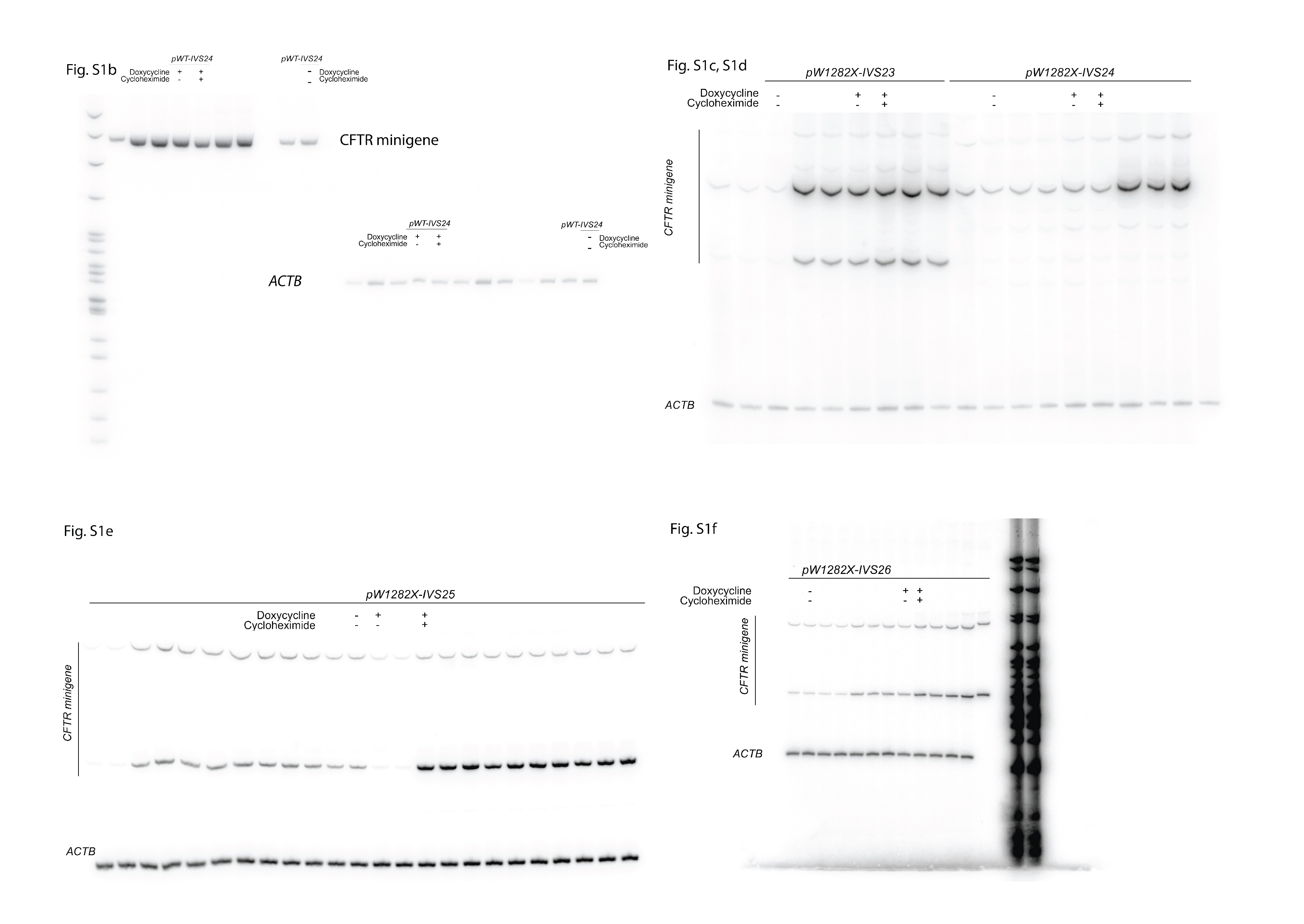

Supplement: Supplementary file 6 — Source Data [file 41467_2022_30668_MOESM6_ESM.zip › Source data, excel/original image summary_Supp fig 1.jpg]

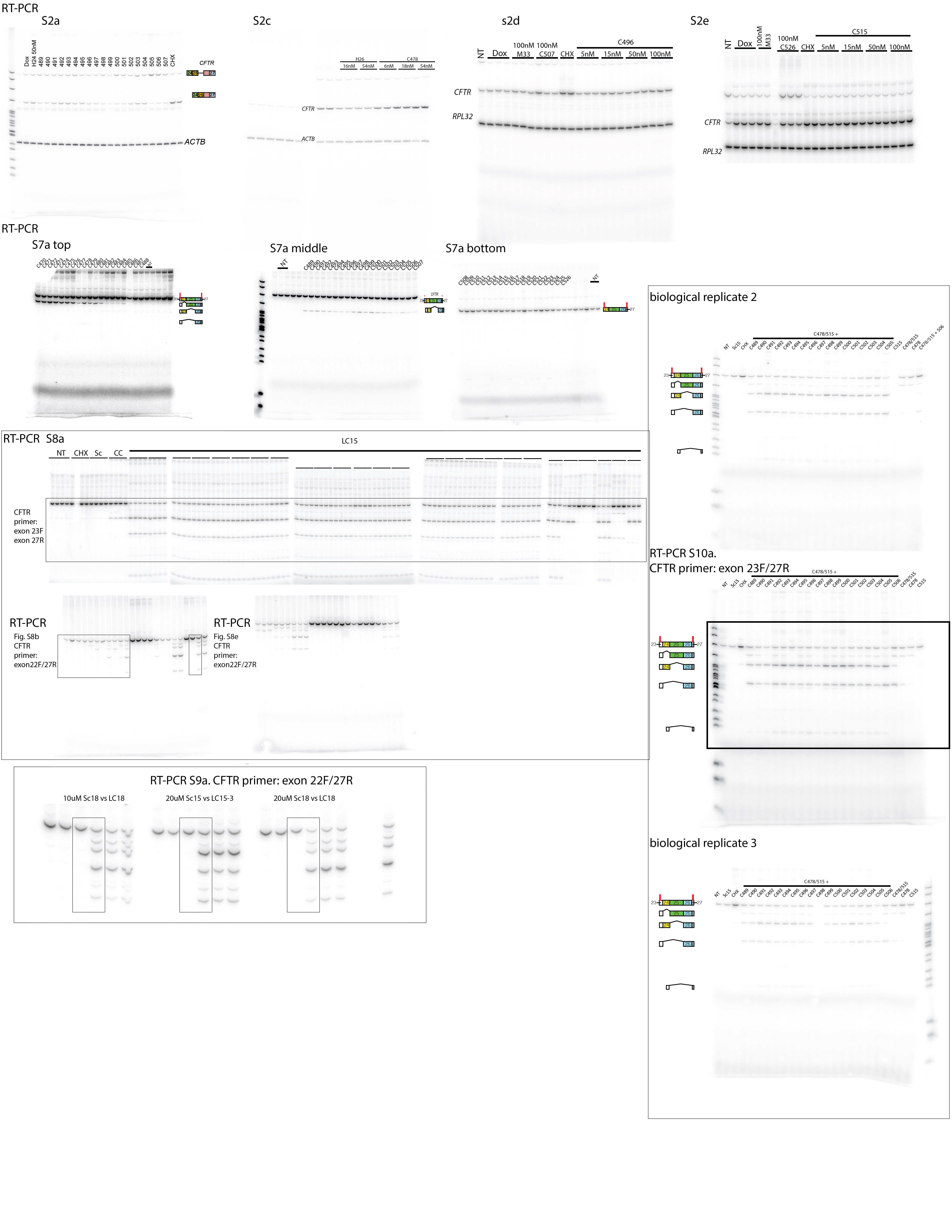

Supplement: Supplementary file 6 — Source Data [file 41467_2022_30668_MOESM6_ESM.zip › Source data, excel/original image summary_Supp fig 2.jpg]

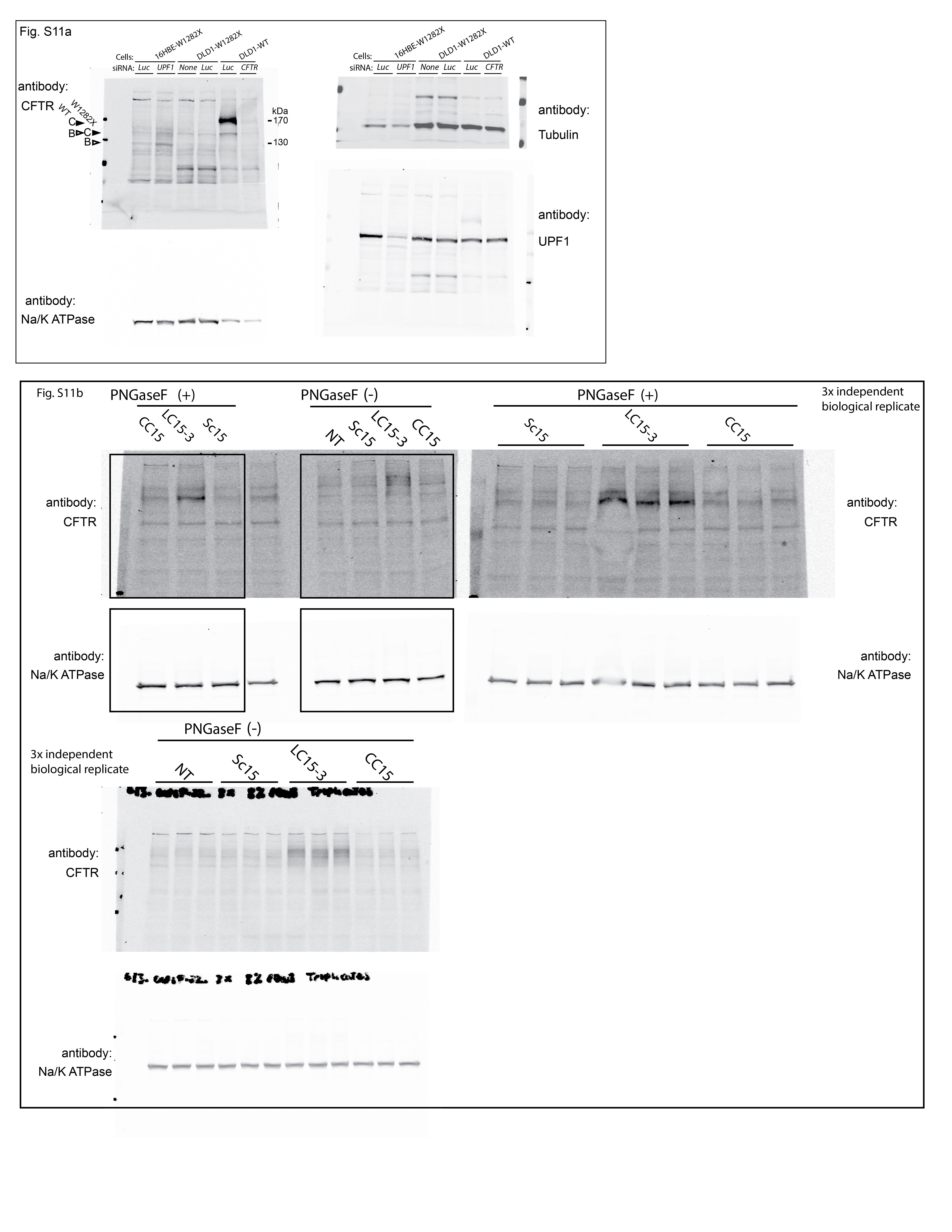

Supplement: Supplementary file 6 — Source Data [file 41467_2022_30668_MOESM6_ESM.zip › Source data, excel/original image summary_Supp fig 3.jpg]

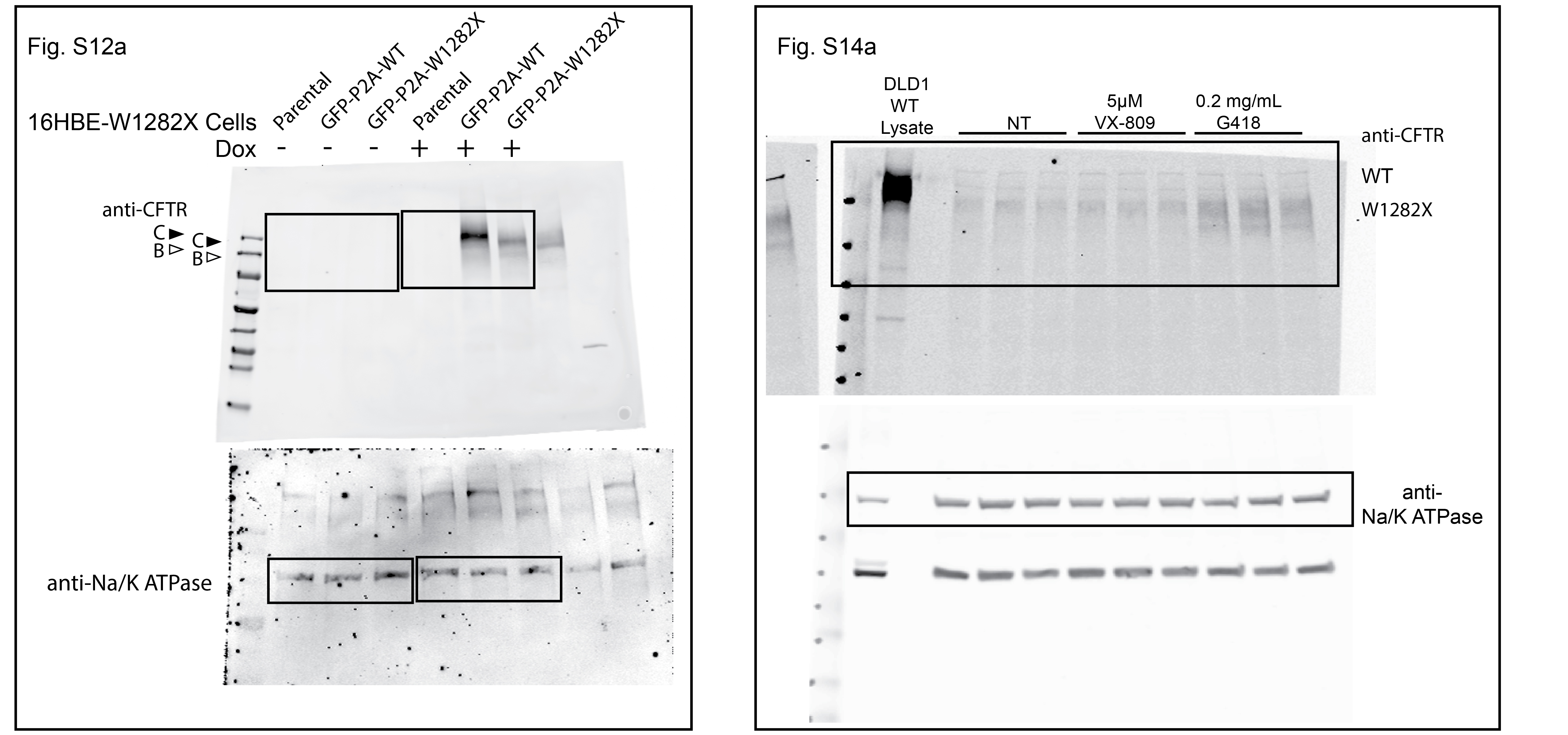

Supplement: Supplementary file 6 — Source Data [file 41467_2022_30668_MOESM6_ESM.zip › Source data, excel/original image summary_Supp fig 4.jpg]
